# Evidence-based comparative severity assessment in young and adult mice

**DOI:** 10.1371/journal.pone.0285429

**Published:** 2023-10-20

**Authors:** Maria Reiber, Lara von Schumann, Verena Buchecker, Lena Boldt, Peter Gass, Andre Bleich, Steven Roger Talbot, Heidrun Potschka

**Affiliations:** 1 Institute of Pharmacology, Toxicology, and Pharmacy, Ludwig-Maximilians-University (LMU), Munich, Germany; 2 RG Animal Models in Psychiatry, Department of Psychiatry and Psychotherapy, Central Institute of Mental Health, Medical Faculty Mannheim, Heidelberg University, Mannheim, Germany; 3 Institute for Laboratory Animal Science and Central Animal Facility, Hannover Medical School, Hannover, Germany; University of Modena and Reggio Emilia, ITALY

## Abstract

In animal-based research, welfare assessments are essential for ethical and legal reasons. However, accurate assessment of suffering in laboratory animals is often complicated by the multidimensional character of distress and pain and the associated affective states. The present study aimed to design and validate multidimensional composite measure schemes comprising behavioral and biochemical parameters based on a bioinformatics approach. Published data sets from induced and genetic mouse models of neurological and psychiatric disorders were subjected to a bioinformatics workflow for cross-model analyses. ROC analyses pointed to a model-specific discriminatory power of selected behavioral parameters. Principal component analyses confirmed that the composite measure schemes developed for adult or young mice provided relevant information with the level of group separation reflecting the expected severity levels. Finally, the validity of the composite measure schemes developed for adult and young mice was further confirmed by *k*-means-based clustering as a basis for severity classification. The classification systems allowed the allocation of individual animals to different severity levels and a direct comparison of animal groups and other models. In conclusion, the bioinformatics approach confirmed the suitability of the composite measure schemes for evidence-based comparative severity assessment in adult and young mice. In particular, we demonstrated that the composite measure schemes provide a basis for an individualized severity classification in control and experimental groups allowing direct comparison of severity levels across different induced or genetic models. An online tool (R package) is provided, allowing the application of the bioinformatics approach to severity assessment data sets regardless of the parameters or models used. This tool can also be used to validate refinement measures.

## Introduction

Animal welfare assessments are an ethical imperative in laboratory animal science. Besides, assessing the extent of prospective, actual, and retrospective severity of animal experiment procedures is a legal requirement in all European Union member states, as stipulated in Directive 2010/63/EU. As stated by Russel and Burch (1959) [1], alleviating animal suffering by eliminating distress- and harm-provoking factors in conjunction with proper pain management can improve the robustness and validity of preclinical data. Thus, animal experiments should be designed to avoid or largely mitigate pain, suffering, distress, and lasting harm experienced by laboratory animals [2]. In implementing the 3R principles, as defined by Russel and Burch (1959) [1], efforts have been made to measure, grade, and reduce the extent of suffering in laboratory animals [3, 4].

Given the multiple facets of emotional consequences associated with impaired well-being, suffering, pain, and distress in laboratory animals, previous findings pointed to the need for multidimensional composite schemes for evidence-based severity assessment [3, 5–9]. Respective schemes are of particular informative value regarding the severity classification of new models, the comparison of different animal models, and the validation of refinement measures [3]. Recently, we have successfully applied a bioinformatics approach to develop composite measure schemes for adult rats [5]. In that study, we could confirm that bioinformatic analyses of comprehensive data sets containing behavioral and biochemical data from various chronic models can guide the design and validation of multidimensional composite schemes allowing sensitive and comparative severity classification and assessment [5, 10–12]. In this context, it should be considered that viable routine scoring systems require a reduction in the number of parameters. Therefore, the suitability of selected behavioral and biochemical parameters has been assessed in different studies focusing on severity assessment in laboratory rodents [10–18]. In this context, it is particularly relevant to determine the losses in informative value by direct comparison with multidimensional composite measurement systems.

This study aimed to further develop and validate composite measure schemes based on comprehensive data sets from different mouse models of neurologic and neuropsychiatric relevance. The first part of our bioinformatics approach focused on models in adult mice comprising the intrahippocampal kainate model [15], the amygdala-kindling model [14], and the hippocampal-kindling model [14]. With the second part of our bioinformatics approach, we focused on severity assessment in young mice from genetic models. The assessment of severity in genetic mouse models presents particular challenges associated with the analysis of cumulative severity and dynamic changes in severity that may occur during early development in young mice. Special care is necessary to avoid overlooking potential severity peaks in susceptible developmental stages of young mice that may, for example, characterize models of neurodevelopmental disorders [19, 20]. To develop composite measure schemes for evidence-based severity assessment in young mice, we have previously assessed various behavioral and biochemical parameters in young C57BL/6J wild-type mice [16], designed a draft composite measure scheme and applied this scheme in two genetic loss-of-function mouse models [17, 18]. These models comprised *Scn1a+/-* mice recapitulating features of a developmental and epileptic encephalopathy, termed Dravet syndrome, and young *Gria1-/-* mice recapitulating features of neuropsychiatric disorders. The models have been selected to validate the draft composite measure scheme based on the fact that different levels of distress are expected in these models.

The present study aimed to design and validate multidimensional measure schemes based on a bioinformatics approach applied to a combination of data sets from various studies. In particular, we tested whether the data sets from adult and young mice provide a basis for categorizing severity levels in individual animals and comparative severity assessment of induced and genetic models. Moreover, we examined whether it is possible to reduce the number of combined parameters to develop simplified schemes without relevant loss of informative value.

## Results

**Fig 1:**
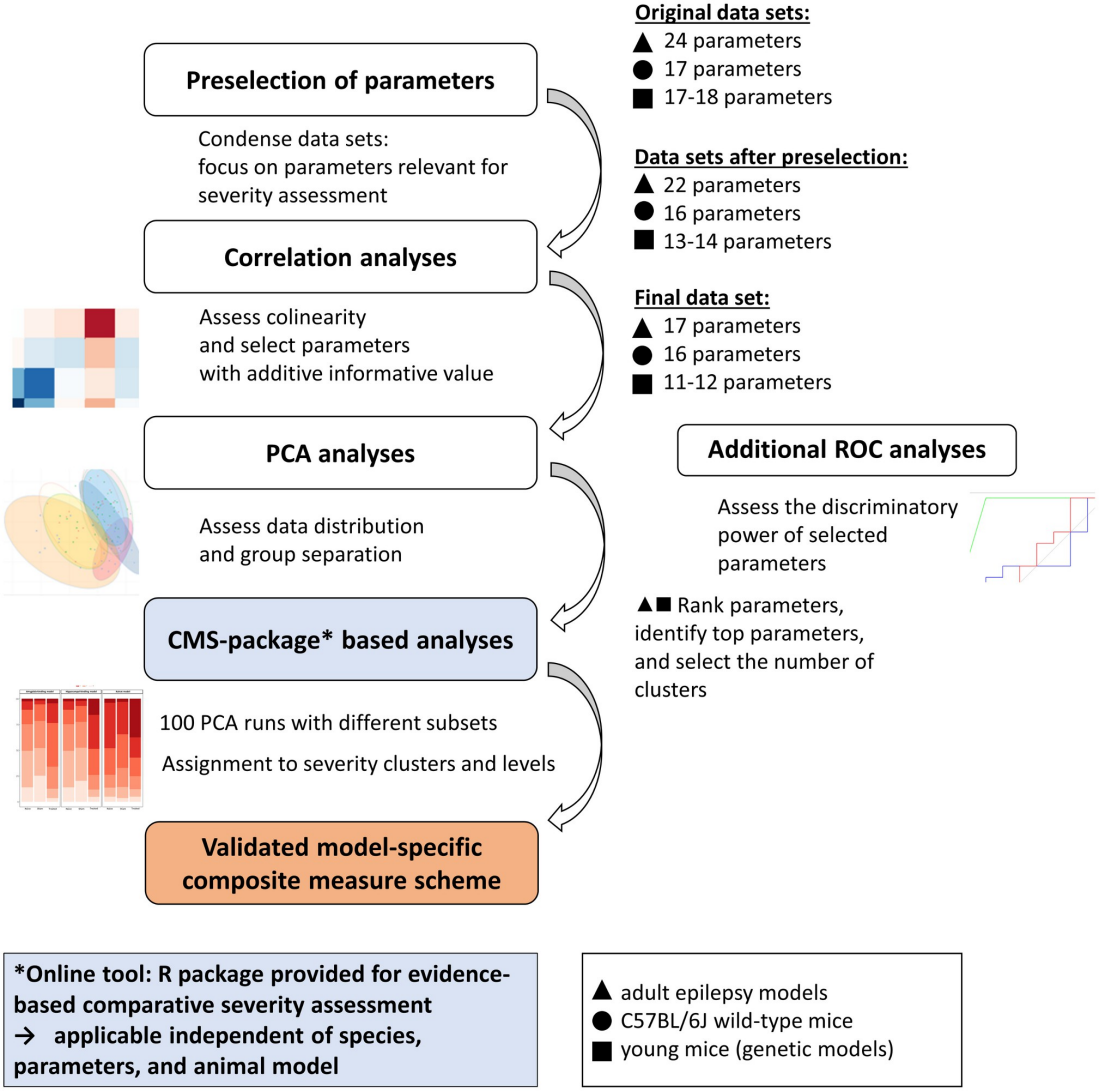
Overview of the bioinformatics workflow. Combined data sets are subjected to a bioinformatics workflow comprising a preselection of parameters, correlation, and principal component analyses. The basic principles of the step-by-step bioinformatics workflow are illustrated on the left side. The right side of the graph describes their practical application to data sets considered in this publication. The approach allowed a reduction of parameters, which contributed to condensation of information on a distress-related model- and laboratory-specific severity. Further analyses, as displayed in the blue boxes, were computed using a newly designed R package: composite-measure-scheme-(CMS)-package, available at: https://github.com/mytalbot/cms. CMS-package-based analyses allow the ranking and selection of parameters, definition of clusters, and the assignment of individual animal data to model-specific severity clusters. The approach can be applied for a comparison of different models and the assessment of the impact of potential refinement measures. In this context, it should always be noted that laboratory-specific factors can influence severity clustering for a given model. In addition, model-specific receiver operating curve (ROC) analyses allow testing for adequate discriminatory power. This study applied ROC analyses to data sets with three selected behavioral paradigms (Irwin test, open field test, sweetness preference).

Combined data sets were subjected to a statistical validation procedure to identify parameters of high informative value for distress-related severity. An overview of the statistical workflow is provided in Fig 1. First, data from three adult induced epilepsy models were combined, comprising project-specific data from the intrahippocampal kainate model (n = 28), the hippocampal-kindling model (n = 41), and the amygdala-kindling model (n = 44). Second, data from C57BL/6 wild-type mice of different ages (n = 200), which can provide information about the variance of baseline behavioral patterns during development, were combined. Third, a combined data set from young mice with genetic deficiencies (i.e., the *Scn1a* model (n = 40) and the *Gria1* model (n = 38)) was divided into two age-specific subsets representing the two time slots of data collection during the development of the mice. One data set consisted of data collected during early adolescence of the mice (postnatal day (P) 23 to P34), while the other one contained data from sexually mature mice aged 48 to 58 days, corresponding to a late adolescent age.

### Adult mice

#### Preselection of parameters

A preselection of parameters was implemented to ensure standardized analyses across all models. First, the data set was tested for data integrity: all parameters with data showing > 20% missing values were removed from the combined data set. The mouse grimace scale (MGS) was removed from the combined data set due to > 20% missing values. Second, absolute body weight in g and % change in body weight were excluded since some of the models used are associated with weight gain, which is likely related to changes in neurotransmission that affect appetite regulation and, therefore, unlikely to be related to an influence of distress [7]. After preselection, the combined data set of adult epilepsy models contained a total number of 22 parameters. A detailed overview of the project-specific parameters in- and excluded for further analyses is provided in Table 1. In implementation of Directive 2010/63/EU, all animals used for the projects were additionally subjected to daily evaluations of the clinical phenotype.

**Table 1:** Overview of the parameters analyzed in the three adult induced epilepsy models, the C57BL/6J wild-type mice, and the two genetic models.

*Model*		*Intrahippo-campal kainate model*	*Kindling models*^2^ *(Hip/ AM)*	*Scn1a model*	*Gria1 model*	*C57BL/6J wild-type mice*
***Subproject No*.**		1	2, 3	4	5	6
***Publication***		Buchecker et al., [15]	Boldt et al., [14]	Reiber et al., [17]	Reiber et al., [18]	Reiber et al., [16]
***Age at observation***		Adult: P80-P140	Adult: P80-P140	Adolescent: P27-P34 P48-P58	Adolescent: P23-P34 P48-P58	Adolescent: P25-P31, P36-P41, P50-P56 Adult: P65-P71, P120-P126
***Groups***		Naïve: n = 8 Sham: n = 8 Treated: n = 12	Naïve ^1^: n = 15 Sham^2^: n = 15/15 Treated^2^: n = 11/14	*Scn1a +/+* n = 20 *Scn1a +/-* n = 20	*Gria1 +/+* n = 20 *Gria1 -/-* n = 18	Wild-type mice of different age groups(n = 40 per group)
***Parameter***						
Weight gain		**E**	**E**	**E**	**E**	**E**
*Nest building*		**X**	**X**	**X**	**X**	**X**
*Level of soiling*		**E**	n.a.	n.a.	n.a.	n.a.
*Burrowing*	*2h Day*	**X**	**X**	**E**_**⌀**_	**E**_**⌀**_	**X**_**1**_ ^4^ **, X**_**2**_ ^4^
	*Night*	**E**	**E**	**X**_**⌀**_ ^3^	**X**_**⌀**_ ^3^	**X**_**1**_ ^4^ **, X**_**2**_ ^4^
Irwin Score		**X**	**X**	**X**	**X**	**X**
Temperature		n.a.	n.a.	**X**	**X**	**X**
MGS		**E**	**E**	n.a.	n.a.	n.a.
Open field	Distance moved	**X**	**X**	**X**	**X**	**X**
	Rearing	**X**	**X**	**X**	**X**	**X**
	Immobile	**E**	**E**	**R**	**R**	**X**
	Center time	**X**	**X**	**R**	**R**	**X**
	Wall time	**E**	**E**	**X**	**X**	**X**
	Jumps	n.a.	n.a.	**X**	**X**	**X**
	Velocity	**E**	**E**	n.a.	n.a.	n.a.
Social interaction	Active	**X**	**X**	n.a.	n.a.	n.a.
	Passive	**X**	**X**	n.a.	n.a.	n.a.
*Saccharin preference*		**X**	**X**	**X**	**X**	**X**
Black-white box	Time in WB	**X**	**X**	n.a.	n.a.	n.a.
	Stretching	**X**	**X**	n.a.	n.a.	n.a.
	Latency	**X**	**X**	n.a.	n.a.	n.a.
Elevated-plus maze	Closed arms	**E**	**E**	n.a.	n.a.	n.a.
	Open arms	**X**	**X**	n.a.	n.a.	n.a.
	Open 1.3	**X**	**X**	n.a.	n.a.	n.a.
	Stretching	**X**	**X**	n.a.	n.a.	n.a.
	Head dip	**X**	**X**	n.a.	n.a.	n.a.
*Voluntary wheel running*		n.a.	n.a.	n.a.	**E**	**X**
Corticosteronemetabolites	Samples: Feces	X	**X**	**X**	**X**	**X**
	Samples: Serum	n.a	**E**	n.a.	n.a.	n.a.
*Telemetric recording*		**E**	n.a.	n.a.	n.a.	n.a.
*Spontaneous seizures (n)*		**E**	n.a.	n.a.	n.a.	n.a.
Induced seizures		n.a	**E**	n.a.	n.a.	n.a.
*PhenoTyper cage*	*Distance*	n.a.	n.a.	**E**	**E**	n.a.
	*Velocity*	n.a.	n.a.	**E**	**E**	n.a.
	*Feeding*	n.a.	n.a.	**X**	**X**	n.a.
	*Drinking*	n.a.	n.a.	**X**	**X**	n.a.
	*Center*	n.a.	n.a.	**E**	**E**	n.a.
**Total amount after selection**		**17**		**11/12**		**16**

Analyses in the three induced epilepsy models (subprojects 1, 2, 3) were conducted in adult mice. Analyses in the two genetic models (subprojects 4, 5) were conducted during early and late adolescence. Analyses in C57BL/6J wild-type mice (subproject 6) were conducted in mice of three adolescent cohorts (prepubescent cohort: P25-P31, pubescent cohort: P36-P41, sexually mature cohort: P50-P56) and two adult cohorts (young adult cohort: P65-P71, mature adult cohort: P120-P126). Parameters assessed in the animals’ home cages are printed in *italic*. All animals were additionally subjected to daily evaluations of the clinical phenotype to implement Directive 2010/63/EU.

**X**: Parameter selected for further analyses; **E**: Parameter was assessed but excluded for further analyses; **n.a.**: Parameter was not assessed.

^**1**^ Naïve animals from subprojects 2 and 3 are identical;

^**2**^ Two subprojects: hippocampal-kindling (Hip) model and amygdala-kindling model (AM);

^**3**^ Burrowing was only assessed in late adolescence and the mean of the two measurements was calculated;

^**4**^ In C57BL/6J wild-type mice, the two burrowing measurements from two consecutive test days were analyzed separately.

#### Analyses of correlation

Spearman correlation was calculated for each of the combined data sets. A significant correlation was defined as follows: the Spearman correlation coefficient was set at < -0.5 or > 0.5 in combination with a p-value < 0.05. This procedure aimed to sort out highly correlated, redundant parameters.

The results of the correlation analysis demonstrated a significant positive correlation between open field distance moved and open field velocity (r = 0,79, p <0.0001), which is due to the calculation of the velocity (average distance per total test duration). On the other hand, a significant negative correlation was detected for several parameters, since the variables represent (partly) mutually exclusive events: center duration with wall duration and distance with immobility, assessed in the open field test, as well as the duration mice spent in the open arm with the duration mice spent in the closed arm, and the duration mice spent in the closed arm with the head dips of the elevated plus maze (range: r = -0.74 to -0.8, p (all measurements) < 0.0001). Based on the correlation analysis, the following highly correlated parameters (cut-off: r > 0.7 or r < -0.7 in combination with a p-value < 0.05) were excluded: duration mice spent in the wall zone of the open field, immobility and velocity displayed in the open field test, and the duration mice spent in the closed arms of the elevated plus maze. In addition, overnight burrowing performance (r = 0.68, p < 0.0001) was excluded since burrowing during the 2-hours light phase is considered a parameter of higher sensitivity. Seventeen parameters were considered for further analysis of combined data sets (Table 1).

Considering the correlation of daily clinical scoring that was applied following Directive 2010/63/EU, significant positive correlations of clinical sum scores with Irwin scores (r = 0.55, p < 0.0001) and passive social interaction time (r = 0.51, p < 0.0001) were detected. A significant negative correlation was detected for clinical sum scores with open-field velocity (r = -0.54, p < 0.0001).

Results from correlation analyses are presented as a heatmap illustrations in S1 Fig. Corresponding abbreviations, p- and r-values are provided in S1, S2a and S2b Tables.

#### Receiver operating curve (ROC) analyses

ROC analyses computing the area under the curve (AUC) represent a conventional statistical method to test for the discriminatory power, based on the specificity and sensitivity of selected tests [8]. Here, we have analyzed a selection of three different parameters for the different models, as follows: first, the saccharin preference test representing a home-cage based observation; second, the Irwin test as a classical neurobehavioral assay applied here as a comprehensive clinical scoring system; third, the open field test, in particular, the total distance moved, as a behavioral test on locomotion and exploratory behavior conducted in an unfamiliar experimental setting.

For the binary classifier, implanted and epileptic mice were defined as ‘with burden’ (given a corresponding value of 1). In contrast, the sham animals (implanted, non-epileptic) were defined as the control group ‘without burden’ (given a corresponding value of 0).

**Fig 2:**
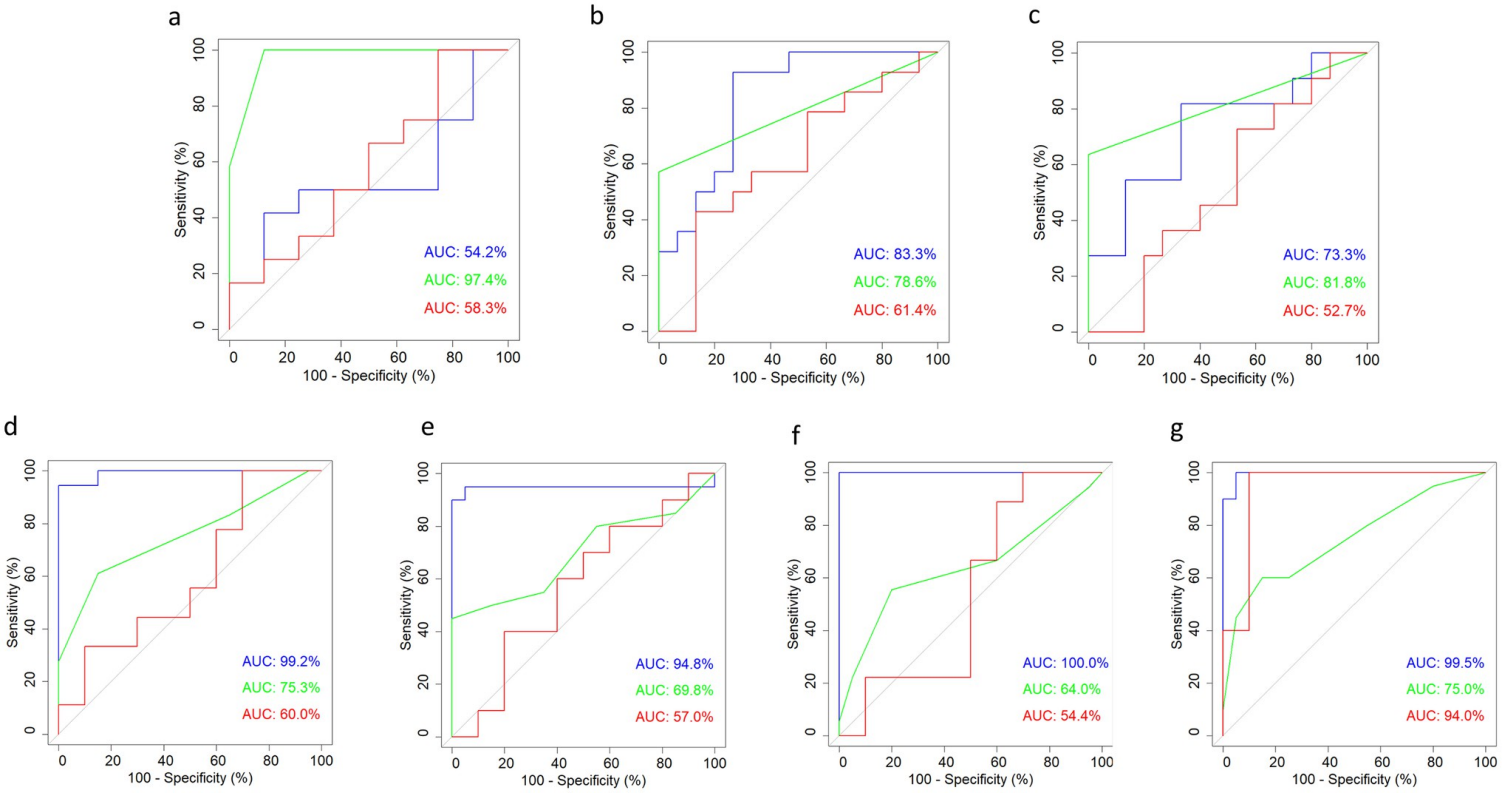
Receiver operating curve (ROC) analyses. The area under the curve (AUC) was computed for each parameter. The following parameters—illustrated by different colors as indicated below—were subjected to ROC analyses across all projects: Distance moved in the open field test (blue), Irwin sum score (green), and saccharin preference (red). (a) intrahippocampal kainate model, (b) amygdala-kindling model, (c) hippocampal-kindling model, (d) *Gria1* model: early adolescence, (e) *Scn1a* model: early adolescence; (f) *Gria1* model: late adolescence, (g) *Scn1a* model: late adolescence. The raw data underlying this figure are available in the Figshare repository https://doi.org/10.6084/m9.figshare.22759148.v1.

ROC analyses identified the Irwin test as the most relevant parameter among the three parameters shown, with an AUC of 97.4% for the intrahippocampal kainate model. In contrast, the readouts from the saccharin preference test and the open field test showed no clear separation between experimental and sham groups (Fig 2a). Concerning respective analyses in the two kindling models, the Irwin and open field tests reached the highest test-specific selectivity. In the amygdala-kindling model, the AUC for the Irwin and open field tests amounted to 78.6% and 83.3%, respectively (Fig 2b). In the hippocampal-kindling model, the AUC of the Irwin test and the open field test amounted to 81.8% and 73.3%, respectively (Fig 2c). While the Irwin test and the open field test reached enough discriminatory power (at least 70%) in both kindling models, the results from the saccharin preference test indicated a relatively poor selectivity (Fig 2b and 2c).

#### Principal component analyses

**Fig 3:**
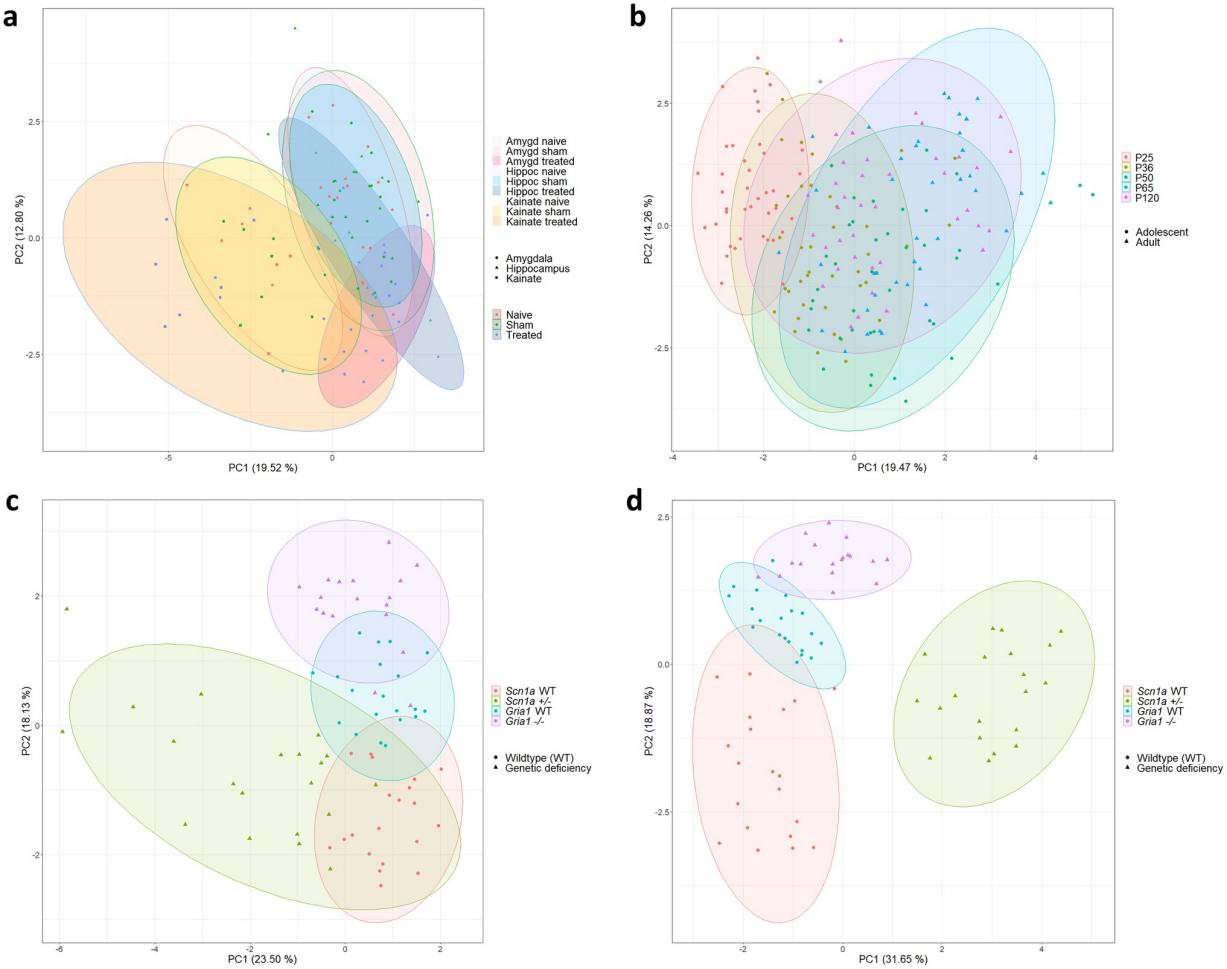
Principal component analyses (PCA). One PCA run is illustrated for all subgroups of the three adult epilepsy models (intrahippocampal kainate model, amygdala-kindling model, and hippocampal-kindling model) (a), the C57BL/6J wild-type mice (b), the *Scn1a* and *Gria1* models assessed during early adolescence (c) and late adolescence (d). The raw data underlying this figure are available in the Figshare repository https://doi.org/10.6084/m9.figshare.22759148.v1.

Data from adult epilepsy models were subjected to principal component analyses. Individual data for PC1 and PC2 are illustrated in Fig 3a with PC1 and PC2 accounting for 19.5% (standard deviation (SD): 1.82) and 12.8% (SD: 1.48) of the total variance, respectively. Illustrations of PC1 and PC2 failed to clearly separate subgroups (experimental vs. sham; experimental vs. naïve; sham vs. naïve) (Fig 3a). For the intrahippocampal kainate model, data from animals exhibiting one or more spontaneous, generalized seizure(s) during the monitoring phase are highlighted in S2 Fig: in total, 7 out of 12 animals in the treatment group of the intrahippocampal kainate model were identified to show one or more spontaneous, generalized seizure(s). Interestingly, data from individual animals clearly separated along PC1 originate from animals with one or multiple spontaneous, generalized seizure(s) during the monitoring phase (S2 Fig). However, for the complete subset of data from individual animals with detected seizure(s), a clear separation along one of the two main principal components could not be confirmed (S2 Fig).

#### Identification of top-ranking parameters

The selection of principal components was based on the principal components PC1 to PC5, each showing an SD > 1. The modified PCA procedure, comprising the 100-fold resampling of a subset of 80% of the data (‘training data set’) along PC1 to PC5, was conducted to identify top-ranking parameters based on their Eigenvalues. These analyses identified the following parameters as top-ranking parameters, accounting for most of the total variance across the data from adult epilepsy models: the number of stretching postures displayed by the animal in the black-white box test and the time the animals spent in the white compartment, the amount of pellets burrowed by the animals in the burrowing test during the light phase session, the time mice spent in social interaction during the social interaction test and nest-building activity. However, individual parameters accounted for a small proportion of the total variance. This finding argues against reducing the number of parameters considered for a composite assessment scheme, as this would mean losing too much informative value. This conclusion is consistent with the general view that the assessment of animal well-being requires multidimensional approaches. Detailed information on ranks of top parameters for the first 30 positions is provided in S3 Table.

#### *k*-means-based cluster analyses

The number of clusters was determined based on a scree plot illustrating the dimensions of explained variances within the combined data set of the adult epilepsy models (S3 Fig). Six clusters were identified as relevant for further analyses. While cluster 6 was defined as the dimension representing the highest severity group, clusters 5, 4, 3, 2, and 1 defined dimensions of a descending, lower severity grade. After that, the *k*-means-based algorithm determined cluster thresholds by 100-fold resampling of the ‘training data’.

**Fig 4:**
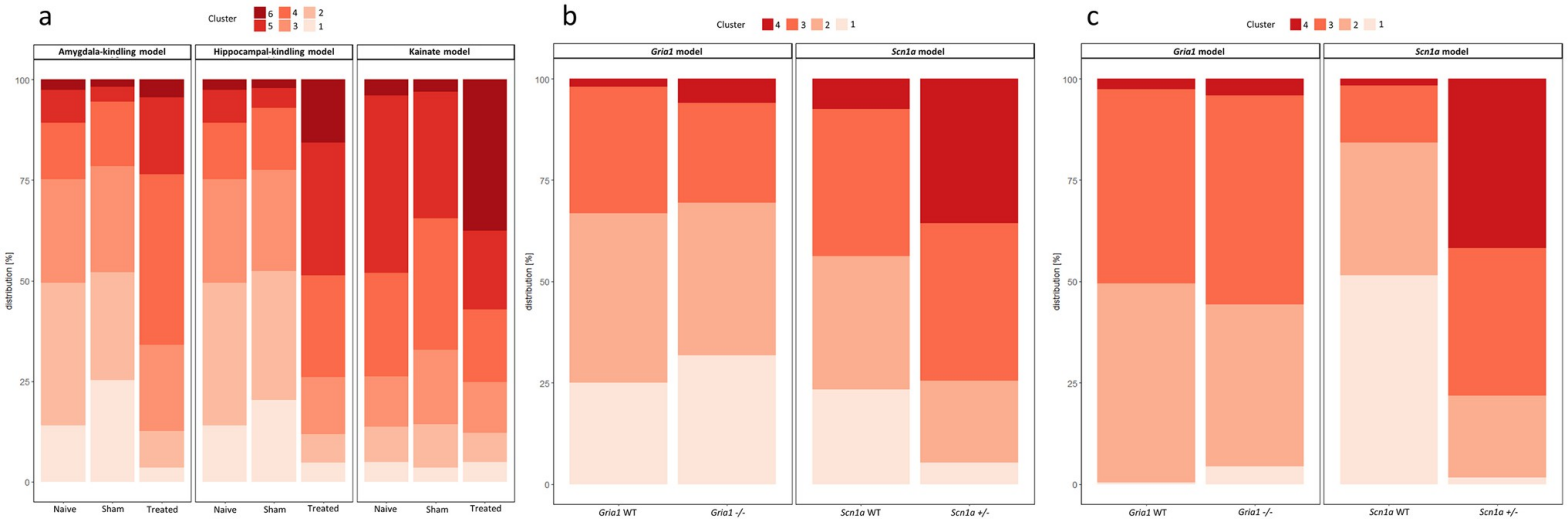
*k*-means-based cluster analyses. The assignment of individual animals to predefined clusters is illustrated for the data from adult epilepsy models (a) and respective data from the *Scn1a* and *Gria1* models in early adolescence (b) and late adolescence (c). Cluster 4 corresponds to the highest severity level, while clusters 3, 2, and 1 each correspond to a lower severity level in gradually descending order (a). Cluster 6 corresponds to the highest severity level, while clusters 5, 4, 3, 2, and 1 each correspond to a lower severity level in gradually descending order (b and c). The raw data underlying this figure are available in the Figshare repository https://doi.org/10.6084/m9.figshare.22759148.v1.

In the intrahippocampal kainate model, 37.5% of the mice from the treatment group were assigned to cluster 6. In comparison, only 3.1% and 3.9% of the mice from the sham and naïve groups were assigned to cluster 6, respectively (Fig 4a). In the two kindling models, the sums from clusters 6 and 5 obtained from experimental mice exceeded the corresponding sums obtained from sham animals as well as naïve animals: in the amygdala-kindling model, 23.5% of the experimental animals were allocated to clusters 6 and 5, whereas only 5.5% and 10.8% of the sham and naïve animals were allocated to clusters 6 and 5, respectively (Fig 4a). In the hippocampal-kindling model, 48.8% of the experimental animals could be assigned to clusters 6 and 5, and only 7.3% and 10.8% of the sham and naïve animals were allocated to clusters 6 and 5, respectively (Fig 4a). Detailed results on cluster allocations are provided in S4 Table.

The determination of severity clusters was computed over the entire array of parameters, since cluster determination reduced to the set of top-ranking parameters did not result in robust clusters. Please note that the decision to include all parameters was also based on the findings from the PCA and general considerations concerning the multidimensional nature of animal well-being.

### Young mice

#### Preselection of parameters

Considering the integrity of data sets from early adolescent and late adolescent mice, data on voluntary wheel running activity (VWR) were removed due to a lack of > 20% of the data. VWR was not assessed in the *Scn1a* model due to the risk of injury, related to spontaneous seizures while running in the wheel. In addition, the following parameters were excluded, since the respective data sets were not considered robust and reliable for animal welfare assessment: home-cage activity, in particular, 1) distance moved, 2) velocity, and 3) the time mice spent in the center of the PhenoTyper cages. This decision was related to the fact that data on home-cage activity had been acquired as pilot data in the PhenoTyper cages, measured only during a time slot of 60 minutes, and therefore do not allow for conclusions about general circadian activity patterns. Moreover, PhenoTyper cages were closed with a top unit rather than a conventional grid on the upper side and therefore did not provide an ‘unprotected’ center. Moreover, the following parameters were excluded due to relevant background strain differences of the genetic models: body weight in g and change of body weight in %.

None of the behavioral parameters were excluded in the data set of the C57BL/6J wild-type mice.

After preselection, the data sets of the genetic models contained 13 parameters for the analysis in early adolescent mice and 14 parameters for the respective analysis in late adolescent mice. The different number of parameters for the two time slots is due to the in-vivo design of the behavioral test battery, which included the burrowing paradigm only for the investigations in late adolescent mice, considering that a relevant burrowing activity is only shown later during development [16]. A detailed overview of the project-specific parameters in- and excluded for further analyses is provided in Table 1.

In implementation of Directive 2010/63/EU, all animals used for the projects were additionally subjected to daily evaluations of the clinical phenotype.

#### Analyses of correlation

Correlation analysis from C57BL/6J wild-type mice was performed across all adolescent age groups, followed by a correlation analysis of data from mature adult mice at an age of approximately 120 days. Correlation analyses across adolescent and mature adult mice showed a significant positive correlation between the two consecutively applied overnight burrowing tests (r = 0.81 and r = 0.78, respectively, p (all measurements) < 0.0001). Therefore, the mean from both measurements was calculated to obtain one informative parameter on burrowing for further analyses. In mature adult mice, a significant negative correlation was identified between saccharin preference and voluntary wheel running performance (r = -0.8, p < 0.0001). Detailed results from Spearman correlation analyses are presented as heatmaps (S4 and S5 Figs) with the corresponding r- and p-values (S5a, S5b, S6a and S6b Tables).

Spearman correlation was calculated for the data set from the two genetic mouse models in early and late adolescence. Analyses of data from early- and late-adolescent mice revealed a significant negative correlation between the duration mice spent in the wall zone and the center zone of the open field arena (r = -0.77 and r = -0.89, respectively, p (all measurements) < 0.0001). Analyses from both time points of data collection also demonstrated a significant negative correlation between the distance moved and the time of immobility in the open field test (r = -0.91 and r = -0.93, respectively, p (all measurements) < 0.0001). Further results from the correlation analyses are presented as heatmap illustrations in S6 and S7 Figs. Corresponding p- and r-values are provided in the S7a, S7b, S8a, and S8b Tables. Based on a strong correlation during early and late adolescence (cut-off: r > 0.7 or r < -0.7 in combination with a p value < 0.05 in both analyses), the following parameters were excluded from principal component and cluster analyses to avoid a predominance of highly correlated parameters: first, the duration mice spent in the center zone of the open field arena, and second, the duration mice displayed immobility during the open field test. The duration mice spent in the wall zone was included for further analyses instead of the duration in the center zone, since data analysis in the original publications showed transient thigmotaxis of young mice. In contrast, center durations did not differ in a significant manner [17, 18].

Considering the correlation of daily clinical scoring that was applied following Directive 2010/63/EU, no significant correlation with other variables was detected (S6 and S7 Figs).

#### ROC analyses

ROC analyses calculating the AUC were computed to assess the model- and age-specific discriminatory power of the three preselected parameters saccharin preference, Irwin score, and distance moved in the open field test. Therefore, individual data from mice carrying the genetic deficiency (*Scn1a+/-* or *Gria1-/-*) were defined as ‘with burden’ (given the corresponding value 1), while data from *Scn1a* and *Gria1* wild-type mice were defined as control groups ‘without burden’ (given the corresponding value 0).

Model-specific ROC analyses (Fig 2) demonstrated that the open field parameter ‘distance’ could distinguish best between the groups across both models and time points (AUC range 94.8%–> 99%). With respect to the Irwin score as a standard neurobehavioral assay, we could show a relatively low test-specific discriminatory power for each model and every developmental stage (AUC range 64.0–75.3%). Notably, the saccharin preference test could differentiate well between *Scn1a* deficient mice and *Scn1a* wild-type mice for the measurements in the late-adolescent age phase (AUC 94.0%). Respective analyses in the *Scn1a* model at an early-adolescent age and analyses in the *Gria1* model at both age stages indicated a relatively poor sensitivity of the saccharin preference test (AUC range 54.4–60%).

#### Principal component analyses

Principal component analyses of the data from wild-type mice at different age phases can provide insight into age-specific variations in behavioral patterns unaffected by a surgically or genetically induced burden. Principal component analyses from the C57BL/6J wild-type mice indicated a clear separation along PC1 only for the group of prepubescent mice (Fig 3b). Interestingly, individual data from mice were more evenly distributed when they had reached sexual maturity (Fig 3b). While the illustration may suggest a slight separation of the pubescent group from the late adolescent group and the two mature adult groups, statistical analysis did not confirm an inter-group difference.

Data from the two genetic models collected during two different time slots in adolescence were subjected to a PCA with individual data plotted for PC1 and PC2, as illustrated in Fig 3c and 3d. Individual data from early adolescent mice, as illustrated in Fig 3c, showed that PC1 and PC2 accounted for 23.5% (SD: 1.61) and 18.1% (SD: 1.41) of the total variance, respectively. Individual data from late adolescent mice, as illustrated in Fig 3d, indicated that PC1 and PC2 accounted for 31.7% (SD: 1.95) and 18.9% (SD: 1.50) of the total variance, respectively. The two-dimensional plot illustrating principal component one and two showed a clear separation of all subgroups for both time points of data collection (Fig 3c and 3d). In the *Scn1a* model, a relevant separation of the genetically modified and wild-type groups became visible, especially for the measurements in late adolescent mice. Interestingly, the two wild-type background strains from the *Scn1a* and *Gria1* model were also segregated in a relevant manner at both time points (Fig 3c and 3d).

#### Identification of top-ranking parameters

One hundred re-samplings of the test data comprising 80% of the total data collected in the genetic mouse models during early adolescence identified the following four parameters accounting for most of the variance along the principal components PC1 to PC4: fecal corticosterone metabolites, the duration mice spent in the feeding and drinking zone of the home cage, and rectal body temperature. Respective analysis of the data acquired from sexually mature mice during late adolescence identified the following four top-ranking parameters: fecal corticosterone metabolites, Irwin score, the duration mice spent in the drinking zone of the home cage, and burrowing performance overnight. However, in the data analyses for both time points, individual parameters did not account for large proportions of the total variance. This finding argues against reducing the number of parameters considered for a composite assessment scheme, as this would mean losing too much informative value. An overview of the ranking of parameters is provided in S9 Table.

#### *k*-means-based cluster analyses

The number of clusters was determined by scree plots illustrating the dimensions of explained variances within the combined data set of the genetic models in early and late adolescence (S8 and S9 Figs). Four dimensions had been selected, accounting for relevant proportions of the variances in the PCA. Cluster 4 was defined as the dimension representing the highest severity group, whereas clusters 3, 2, and 1 defined clusters of lower severity grades.

With respect to cluster analyses of early adolescent mice, 35.7% of the *Scn1a+/-* group were allocated to cluster 4 (cluster of ‘highest severity’), while only 7.5% of the *Scn1a* wild-type group were assigned to this cluster (Fig 4b). Respective analyses of data from early adolescent *Gria1-/-* mice indicated a trend with *Gria1-/-* mice being allocated to clusters of higher severity more frequently (Fig 4b).

Cluster analyses of late adolescent mice revealed relevant differences in cluster distribution for the data from the *Scn1a* model: while cluster 4 comprised 41.7% of the individual data from *Scn1a+/-* mice, only 1.6% of the *Scn1a* wild-type group were allocated to this cluster (Fig 4c). Conversely, cluster 1 comprised 51.5% of the individual data from *Scn1a* wild-type mice and only 1.6% of the individual data from the *Scn1a+/-* group (Fig 4c). Cluster analysis of individual data from the *Gria1* mouse model collected during late adolescence failed to confirm relevant differences in cluster distribution between *Gria1-/-* mice and *Gria1* wild-type mice (Fig 4c). A detailed overview of the cluster distributions is provided in S10 Table.

## Discussion

In this study, we have designed and validated multidimensional composite measure schemes for severity assessment in young and adult mice. The study focused on the question whether the application of the bioinformatics workflow for the design of composite measure schemes to data sets from different mouse models provides a basis for severity classification and comparative severity assessment. We also assessed whether a reduction in the number of parameters is possible without relevant loss of informative value.

Previous studies have applied ROC analysis to determine the sensitivity and specificity of potential severity assessment parameters. High AUC values indicating a solid discriminatory power have, for instance, been reported for burrowing, fecal corticosterone metabolites, and voluntary wheel running in a mouse model of pancreatic cancer, as well as for a combination of burrowing and a clinical distress score in mouse models of liver damage [8, 21, 22]. Our study has exemplarily completed a ROC analysis for selected parameters. This analysis pointed to the fact that the discriminatory power of severity assessment parameters largely depends on the animal model. For example, in the intrahippocampal kainate model, the highest AUC was determined for the Irwin score.

In contrast, in the genetic models, AUCs for the distance moved in the open field reached the highest levels. Saccharin preference only stood out with high discriminatory power in *Gria1-/-* mice during late adolescence. These findings support the use of multidimensional composite measure schemes in particular if the severity of new animal models is explored for the first time or if the aim is to compare the severity of different interventions and models. Applying ROC analysis to assess the discriminative ability of severity assessment requires considering that compromised well-being, suffering, and distress in laboratory animals are not all-or-none phenomena. Thus, a binary classification system does not reflect that severity levels occur on a continuous scale. In addition, we must remember that we do not yet have, and probably never will have, a gold standard combination of severity assessment parameters that reflects the actual truth about severity. Finally, the binary classification in ‘control groups = without burden’ and ‘experimental groups = with burden’ that serves as the basis for ROC analysis is an oversimplification neglecting inter-individual differences in both groups. Pronounced inter-individual differences have been evident in previous studies focused on evidence-based severity assessment in laboratory rodents (e. g., [5, 8, 23, 24]). Considering these limitations, data from ROC analysis should be interpreted with caution in the context of severity assessment. Moreover, ROC analysis can only complement other bioinformatics approaches for validating severity assessment parameters and their combinations.

Cross-correlation analyses across comprehensive data sets from various models can guide the selection of parameters, particularly by identifying parameters that consistently show a high correlation level and might not provide added value. Based on a strong correlation, one can sort out those parameters requiring more elaborate approaches. Previously, we reported that cross-correlation analysis indicated that telemetric parameters requiring invasive procedures to chronically implant transmitters did not appear to have added value over behavioral paradigms (e.g., saccharin preference, nest building, and burrowing activity) in rat epilepsy models [10–12]. Interestingly, high correlation levels in the present study’s mouse data sets were somewhat limited to parameters analyzed in the same behavioral paradigm. Thus, we could only exclude respective parameters for the subsequent PCA and cluster analysis. The finding further supports the need for multidimensional composite measure schemes for severity assessment in mice. In the context of multidimensionality, it should be emphasized that body weight reduction has proven as a valid but not fully comprehensive criterion to define the humane endpoint in animal experimentation [3, 7]. In this study, potential effects of body weight on several behavioral readouts (e. g., burrowing, Irwin test, open-field activity) should be considered. In rodent epilepsy models, excessive weight gain may be observed due to neurotransmitter imbalances [25]. In general, the bidirectionality of body weight and its model-specific relevance to severity assessment should be considered.

In a recent study, we applied PCA as guidance for parameter selection to design a composite measure scheme for rats [5, 10–12]. Identification of top parameters best distinguishing between the groups revealed those parameters which predominantly contributed to the variation in the data sets and differences between groups. Based on these findings, we could develop and validate a composite measure scheme comprising burrowed weight in the burrowing test, distance moved in the open field, time spent in social interaction, and saccharin preference [5]. Furthermore, the combination of these parameters received further confirmation by subsequent identification of severity clusters and a meaningful severity categorization based on them in naïve, sham implanted, and experimental groups from three studies with different rat epilepsy models.

In apparent contrast, the PCA of our comprehensive mouse data sets from both adult and young mice did not identify top parameters that predominantly contributed to the variation in the data sets. These findings suggest an added value of all parameters combined in the comprehensive analysis. The comparison with the composite measure scheme that we have recently developed for rats indicates that there may be pronounced species differences in robust and sensitive evidence-based severity assessment with mice requiring a more comprehensive set of behavioral and biochemical parameters than respective approaches in rats. However, in this context, the influence of the models included in each analysis must be considered.

As previously discussed [3, 26, 27], the application of comprehensive composite schemes is, of course, feasible and worthwhile when it comes to evidence-based 1. assessment and classification of new models, 2. comparative analysis of different models as a basis for animal-welfare based recommendations for selection of an animal model, and 3. evidence-based validation of refinement measures by comparison between groups with and without the potential refinement measure.

On the other hand, the elaborate and time-consuming nature of such schemes would pose a major challenge regarding routine daily monitoring of individual severity levels in ongoing studies [3, 23, 28]. In this context, it is of interest that our data sets point to individual parameters, which may be of particular model-specific value. Thereby, our data from young mice confirmed that it is of utmost relevance to consider the developmental stage of the animals for selecting such parameters. Selected individual parameters might then be added to traditional clinical scores to increase sensitivity concerning more nuanced and reasonable alterations in the animals’ affective state. In this context, parameters that can be assessed in the home cage, e.g., saccharin preference, are particularly interesting. This fact is also reflected by intense efforts to develop approaches for continuous home-cage based monitoring of activity and resting patterns, food and water intake, and physiological parameters (e.g., respiratory rate, heart rate), generating data that will complement information from daily clinical scoring and can also help to implement early humane endpoints (e.g., [29–33]).

To our knowledge, unsupervised *k*-means algorithm-based cluster analysis has first been applied by Häger and colleagues (2018) [23] to define individual severity levels based on body weight and voluntary wheel running data from a mouse model of chemically induced colitis, a mouse model of pancreatic cancer, and mice with serial blood sampling [21, 34, 35]. Subsequently, this approach has been successfully applied to other rodent models, including various neuroscientific rat models [24], a mouse model of pancreatic cancer subjected to chemotherapies [22], and a mouse model of chemically induced colitis [28].

Moreover, we have already successfully integrated *k*-means clustering in a bioinformatics workflow for designing, validating, and assessing a composite measure scheme for severity assessment, previously applied to data sets from rat epilepsy models [5]. Subjecting the comprehensive data sets from adult and young mice to *k*-means clustering resulted in identifying six clusters for adult mice and four clusters for young mice. Along the line of previous findings in rats [5], individual categorization of severity levels confirmed relevant inter-individual differences in all groups of naïve, sham-implanted, and experimental mice regardless of the disease model used. Moreover, the categorization provided a meaningful basis for comparative severity assessment across models.

When comparing the three epilepsy models in adult mice, the largest proportion of animals allocated to the highest severity levels is evident for experimental animals from the kainate model. A direct comparison of experimental animals from both kindling paradigms suggests that hippocampal kindling is associated with a higher burden and distress level for the animals than amygdala-kindling. Therefore, an animal welfare-based preference for amygdala-kindling over hippocampal-kindling and for kindling over the intrahippocampal kainate model is recommended if the working hypothesis can be addressed in the same way with each of these models. Interestingly, a trend for a higher proportion of animals allocated to higher severity levels was evident when comparing naïve animals versus sham-implanted animals. A comparable phenomenon was previously observed in rats [5]. Again, this observation is likely related to the fact that naïve animals were not subjected to a habituation phase with handling and were only subjected to transport to a laboratory or a stay in a laboratory environment during the behavioral paradigms. This bias must be considered along with batch-specific intra-laboratory influencing factors such as experimenter and time of the year.

As already emphasized in the introduction, dynamic changes in distress occurring during the developmental trajectory need to be carefully considered when classifying severity in new genetic mouse models. In this context, a major limitation is related to the fact that efforts to identify and select parameters for evidence-based severity assessment in mice have so far focused on young adult and adult mice [4, 8, 9, 14, 15, 22, 35, 36–39]. Thus, it is of utmost relevance to determine the cross-age generalizability of severity assessment parameters that have already been successfully applied in adult mice. To develop a composite measure scheme that can be used during highly sensitive phases of early mouse developmental stages between weaning and an adult stage, we have preselected candidate parameters based on consortium data obtained from models in adult mice [7, 8, 14, 15, 23, 34, 40]. While the analysis in various age ranges in young C57BL/6J mice revealed several relevant age- and sex-related differences, it confirmed the suitability of all parameters except for burrowing behavior, which could only be applied during a later developmental stage corresponding to late adolescence in humans [16]. Based on these findings, we have designed a composite measure scheme, which was further validated by application in two genetic mouse models, for which we expected a moderate severity in one model with *Scn1a* deficiency [17, 41–43] and a lack of severity or mild severity in the other model expressing *Gria1* deficiency [18, 44–47]. PCA of data sets from young C57BL/6J mice demonstrated age-dependent changes in the intra-group variance observed in the different age groups, with the lowest variance evident in prepubescent mice and increase once the animals reach sexual maturity. These findings confirm an increase in inter-individual differences and variance along the developmental trajectory in young mice and highlight the relevance of individualized behavioral patterns developing in laboratory mice over time despite the highly standardized environment in an animal facility. The data support previous studies describing peaks of distress that can occur over the sensitive developmental phase of mice [19, 20, 48–50]. As expected, PCA of the data sets from the genetic models confirmed that the separation from the corresponding wild-type group was more pronounced in *Scn1a+/-* mice than in *Gria1-/-* mice. It is of additional interest that the separation from wild-type mice became more extreme during late adolescence, thereby recapitulating the ‘so-called’ worsening phase observed in pediatric patients [51–54]. This finding provides first evidence that the composite measure scheme applied in both models allows distinguishing between models with different severity grades. The data sets also demonstrated that the age-dependent development of the intragroup variance could be significantly affected in a disease model, implying that this fact needs to be carefully considered for sample size calculations during study planning.

The validity of the composite scheme developed for young mice was further confirmed by *k*-means-based clustering and allocation of individual animals with or without genetic variants to the different severity levels. More animals from the *Scn1a+/-* model were allocated to the highest severity level compared to the *Gria1-/-* model. In contrast, only minor differences in the distribution to severity levels were evident when comparing *Gria1-/-* and the corresponding wild-type mice. In line with our previous conclusions [18], these data suggest a very low severity level for this genetic model.

Regarding the severity levels applied to comparative severity assessment in this study, it is essential to note that these cannot be directly translated to severity levels defined by the ‘EU Directive 2010/63 on the protection of animals used for scientific purposes’. Of course, the highest severity level defined by *k*-means clustering in our study is limited to the severity in the model with the highest level of distress and suffering used for the analysis. Therefore, it must be considered that there is no direct transferability of the bioinformatics readouts to the severity levels as defined by the EU Directive 2010/63, which should be regarded as a fundamental limitation of the study. Following Directive 2010/63/EU, the actual severity of pain, suffering, distress or lasting harm need to be considered for severity classification. Further data from animal models of different pathophysiologic background and disease phenotype are required to increase diversity and enable transferability to the regulatory level. Thus, it will be of utmost relevance to apply the bioinformatics approach to a range of models or interventions spanning the entire range of expected severity grades, including a high severity according to the EU Directive. In this context, it is emphasized that we provide, along with this publication, a user-friendly online tool that allows an analysis of all kinds of respective data sets regardless of the parameters analyzed and irrespective of the animal models or intervention used (https://github.com/mytalbot/cms). Please note that this tool has been significantly optimized compared to the first version provided in 2020.

In conclusion, the bioinformatics approach confirmed the suitability of the composite measure schemes for evidence-based comparative severity assessment in adult and young mice. In particular, we demonstrated that the composite measure schemes provide a basis for an individualized severity classification in control and experimental groups as a basis for the direct comparison of severity levels across different induced or genetic models. In addition, an online tool is provided, allowing application of the bioinformatics approach to severity assessment data sets regardless of the parameters or models used. This tool can also help to validate refinement measures by comparing groups with or without applying a potential refinement measure.

## Material and methods

### Animal models

The published data sets used and re-analyzed in this study originated from three different mouse models of epilepsy investigated at an adult age [14, 15] and two genetically modified mouse models of neuropsychiatric relevance investigated at an age that corresponds to an infant and adolescent age in humans [17, 18]. Analysis was complemented by data from an additional study in young and adult wild-type mice focusing on developing home-cage based behavioral patterns [16]. This manuscript will refer to the developmental age brackets of prepubescence (P23-P34) and sexual maturity (P48-P60) as ‘early adolescence’ and ‘late adolescence’, respectively, as described previously by Brust and colleagues (2015) [19].

#### Adult mice: Kainate model and kindling models

The data used for this bioinformatics analysis was initially published in two individual studies, using three different mouse epilepsy models (intrahippocampal kainate model, hippocampal-kindling model, and amygdala-kindling model) [14, 15]. Severity was assessed using a comprehensive set of behavioral and biochemical parameters in all three epilepsy models. For the intrahippocampal kainate model, severity was additionally assessed using telemetric recording. Telemetric electrocardiogram (ECG) parameters and activity data were assessed as potential measures of distress and a possible refinement measure compared to the method of continuous monitoring using a tethered connection. The impact of two different localizations of electrode implementation was tested in the kindling models. The aim was to identify and validate the chosen parameters for severity assessment and compare the two models’ burden. An overview of the two different originally published studies and their specific aims and methods is provided in Table 1. Data from 98 female mice (HsdWin:NMRI, Envigo, Horst, Netherlands) were included in a combined data set. All studies were carried out after an acclimatization period of at least ten days with daily handling by experimenters. The body weight of the animals was recorded weekly and a day-to-day control of the mice was performed according to severity assessment schemes, including the Mouse Grimace Scale [55] and a modified Irwin Score [56]. Further information about husbandry conditions is provided in the S1 Appendix.

#### Surgery

The respective original publications (Table 1) contain a detailed description of all procedures performed. In short:

Implantation of a bipolar, Teflon-isolated stainless-steel electrode was performed under general anesthesia (chloral hydrate 440–500 mg/kg, i.p., solved in saline, Merck KGaA, Darmstadt, Germany) in all animals. Meloxicam (Metacam^®^ 5 mg/ml, Boehringer Ingelheim, Germany; 1–5 mg/kg, s.c. 30 min prior and 24 h following surgery) was administered for perioperative analgesia. Local anesthesia was performed by subcutaneous infiltration of the incision area and by direct administration on the skull’s periost (Bupivacaine 0.25% with epinephrine 0.0005% Jenapharm^®^; Mibe GmbH, Brehna, Germany). Depth electrodes were implanted in the right hippocampus (intrahippocampal kainate model & hippocampal-kindling model; coordinates relative to bregma: AP-1.8 mm, L+1.6 mm, DV+1.7 mm) or the right basolateral amygdala (amygdala-kindling model; coordinates relative to bregma: AP-1.0 mm, L+3.2 mm, DV+5.3 mm).

In the intrahippocampal kainate model, a telemetric transmitter was implanted subcutaneously in the dorsocaudal part of the scapula region. Animals received a subcutaneous infiltration of bupivacaine (0.25%, s.c.; Jenapharm^®^, Mibe GmbH, Brehna, Germany) in the incision area in addition to the drugs used for general anesthesia and analgesia. The ECG leads were intramuscularly fixed (negative lead at the right pectoral muscle, positive lead next to the xiphoid).

#### Electrical kindling model [14]

Electrical kindling was performed as described previously [57, 58]. Following a postsurgical recovery period of two weeks, kindling stimulations were initiated in amygdala-implanted (amygdala-kindling model) and hippocampus-implanted (hippocampus-kindling model) mice. On the first day of stimulation, the initial afterdischarge duration (ADT) was determined for each animal. Following initial ADT determination, animals received daily suprathreshold stimulations (five days a week, between 1 and 3 p.m.) with 700 μA (1 ms, monophasic square-wave pulses, 50 Hz for 1 s) for five weeks.

#### Chemical post-status epilepticus model [15]

Kainate injections were performed according to Groticke, Hoffmann [59]. Kainate (50 nl of a 20 mM solution, i.e., 0.21 μg in 50 nl saline, i.e., 1 nmol, Sigma-Aldrich, Steinheim, Germany) was injected into the right CA1 area of the dorsal hippocampus (coordinates relative to bregma; AP- 1.8 mm, L+ 1.6 mm, DV+ 1.7 mm) using a 0.5 μl microsyringe.

#### Young mice: C57BL/6J wild-type mice and genetic models

In the present study, data from three original, separately published in-vivo studies were used (Table 1): the design of the test battery was based on the readouts obtained from behavioral investigations in C57BL/6J wild-type mice [16]. In that study, the general suitability of the different paradigms for implementation in young mice had been assessed [16]. Subsequently, mice of the *Scn1a* model [17] and the *Gria1* model [18], which both represent genetic *loss-of-function* models of neurologic and neuropsychiatric relevance, respectively, were subjected to a modified version of the behavioral test battery. Due to ethical and legal reasons, mice were not housed individually but were kept in cages per experimental unit (n = 2) [16–18].

#### C57BL/6J wild-type mice [16]

As described previously [16], experimental animals (n = 200, ratio female:male 1:1) were obtained from time-mated, pregnant C57BL/6JRj mice (n = 51), generated by Janvier Labs (Le Genest-Saint-Isle, France). Experimental animals (n = 200) were born and raised at the Institute of Pharmacology, Toxicology, and Pharmacy, LMU Munich. Details on husbandry conditions of pregnant mice and experimental offspring mice are provided in the S1 Appendix. Following weaning at P21, offspring mice were housed per experimental unit (n = 2). One experimental unit consisted of same-sex siblings. In cases with more than two same-sex siblings per litter available, group allocation was determined randomly (www.randomizer.org). Group allocation represented three different age brackets in murine adolescence [19]: 1) prepubescent mice (n = 40), experiments starting at P25; 2) pubescent mice (n = 40), experiments starting at P36; and 3) sexually mature mice (n = 40), experiments starting at P50. Data from adolescent mouse cohorts were compared with those obtained from young adult mice (n = 40), experiments starting at P65, and mature adult mice (n = 40), experiments starting at P120. The five different cohorts of mice were housed in our Institute and, having reached the respective age, were subjected to the test battery. An overview of the test battery is provided in S10 Fig.

#### *Scn1a* model [17]

Mice harboring a heterozygous *loss-of-function* mutation in the *Scn1a* gene encoding the alpha 1 subunit of the sodium channel Na_v_1.1 model behavioral and pathophysiologic features of Dravet syndrome, a severe epileptic encephalopathy with a typical onset in infancy. In 70–80% of patients with Dravet syndrome, a heterozygous *loss-of-function* mutation in the *SCN1A* gene is identified [51, 60]. The occurrence of comorbidities follows seizure onset in infants during the progression of the disease, among others, psychomotor regression, ataxia, autistic-like behavior, cognitive and attention deficits, and circadian rhythm defects [51, 61, 62]. In addition, Dravet syndrome is characterized by a high rate of drug resistance [62, 63]. As described previously [17], experimental animals (n = 40) were bred and raised at the Institute of Pharmacology, Toxicology, and Pharmacy, LMU Munich. The in-house breeding colony comprised the parental lines B6(Cg)-*Scn1a*^*tm1*.*1Dsf*^ /J [64, 65] (JAX stock #026133) and 129S1/Sv-*Hprt*^*tm1(CAG-cre)Mnn*^ /J [66] (JAX stock #004302). Further details on breeding, husbandry, dietary nutrition, and additional intense care measures are provided in the S1 Appendix. Group allocation was determined according to the *Scn1a* genotype with an experimental group (n female = 10, n male = 10) expressing the heterozygous A1783V-*Scn1a* mutation and a control group (n female = 10, n male = 10) carrying the A1783V-*Scn1a* wild-type variant. The genotype of the mice for *Cre* recombinase was not considered to have a relevant impact on the behavioral phenotype [43]. The onset of seizures was detected in offspring mice aged 16 days as described previously [17, 43].

#### *Gria1* model [18]

Mice harboring a homozygous *loss-of-function* mutation in the *Gria1* gene model features of neuropsychiatric diseases related to glutamatergic dysfunction [45, 47, 67–70]. Evidence exists that GluA1 depletion can induce behavioral impairments in adult mice, among others, spatial working memory deficits with an intact reference memory [44, 71–73], novelty-induced hyperexcitability [68], hyperlocomotion [68, 72, 74], and depression-associated phenotypical traits [75, 76]. As described previously [18], experimental animals (n = 38) were bred at the Institute of Pharmacology, Toxicology, and Pharmacy, LMU Munich. Breeding animals were obtained from the animal facility of Heidelberg University (IBF, Heidelberg). *Gria1* knockout (*Gria1-/-)* mice (line B6N.129-*Gria1*^*tm1Rsp*^/J, available at The Jackson Laboratory: Strain #019011), Mouse Genome Informatics ID: MGI:2178057) had been generated as described previously [44]. Further details on breeding and husbandry conditions are provided in the S1 Appendix. Group allocation was determined according to the *Gria1* genotype, with the experimental group expressing the *Gria1-/-* variant (n female = 10, n male = 8) and the control group harboring the *Gria1* wild-type variant (n female = 10, n male = 10).

### Analysis of behavioral and biochemical parameters

**Fig 5:**
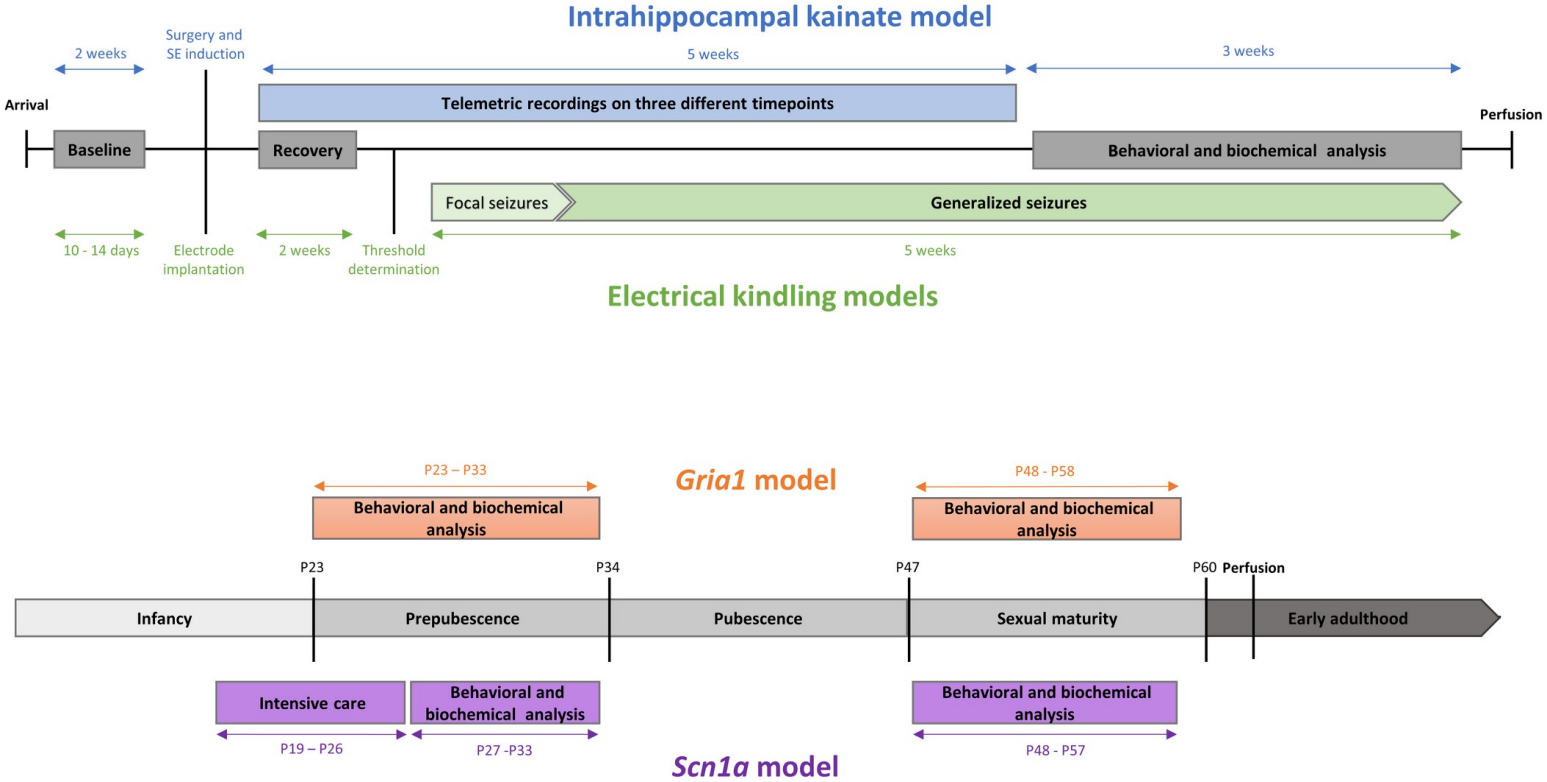
Overview of the experimental timelines. a adult epilepsy models, b young genetic models.

**Fig 6:**
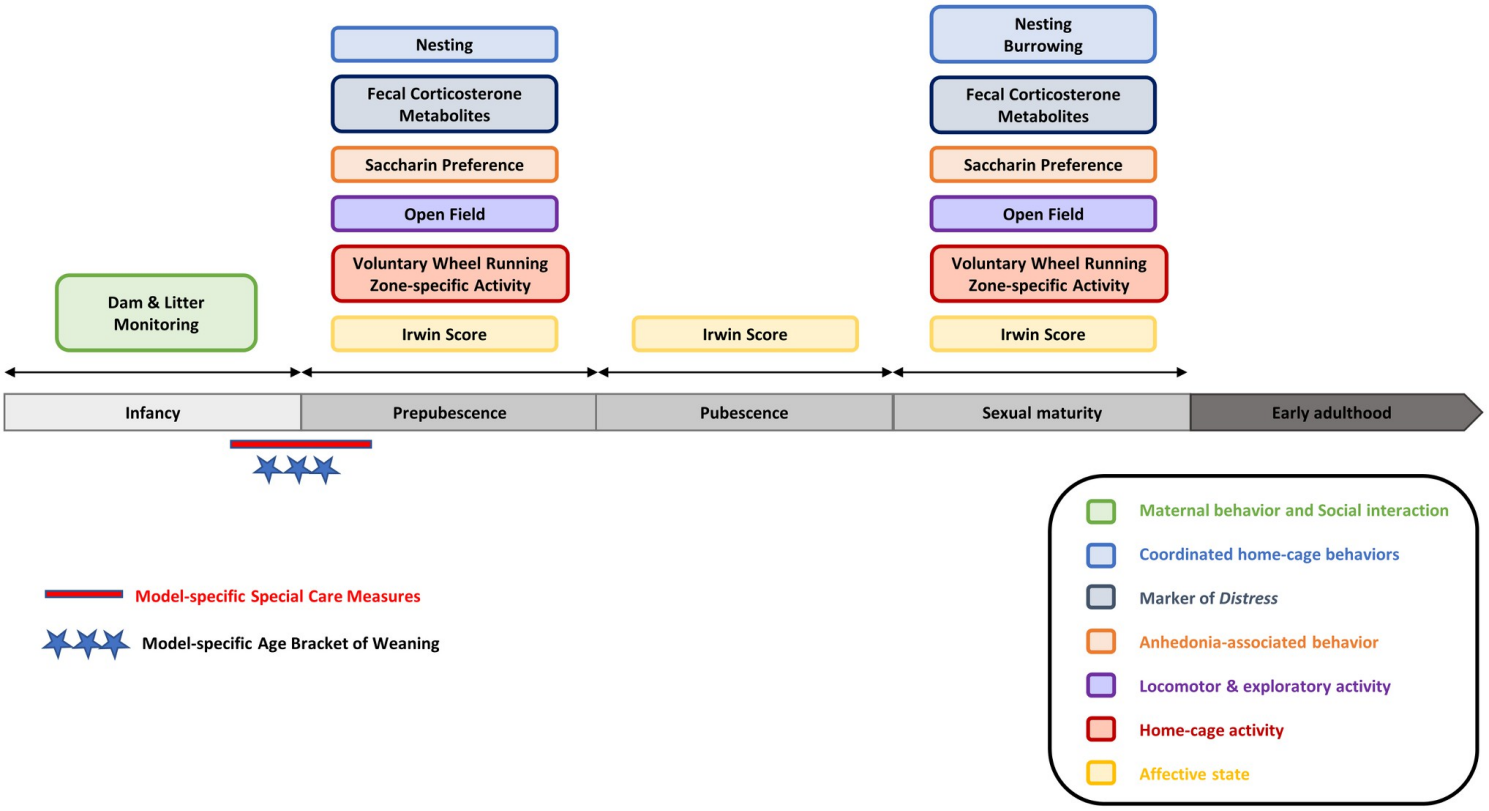
Overview of the test battery for young mice. The assembly of the test battery and the selected age brackets of observation need to be adjusted according to model-specific impairments. Please note that for the purpose of severity assessment, wheel running performance should not be assessed in epilepsy models and models with impairment of sense perception and/or disturbance of locomotor muscle coordination due to the risk of injury.

In the original studies, mice were subjected to a behavioral test battery, complemented with the non-invasive determination of fecal corticosterone metabolites (FCM). The test assembly was based on an extensive set of formerly validated parameters in adult rodent epilepsy models [5, 10–12]. For the projects in the adult epilepsy models (intrahippocampal kainate model, hippocampal-kindling model, and amygdala-kindling model), we used data from behavioral and biochemical assessments in the chronic phase (intrahippocampal kainate model) and phase of generalized seizures (amygdala-kindling and hippocampal-kindling model). A respective timeline for the investigations in the three models during the late phase is provided in Fig 5a. For the projects conducted in the genetic models, behavioral tests and FCM analyses were applied twice during two developmental stages in adolescence: first, during early adolescence in prepubescent mice, and second, during late adolescence, when mice had reached sexual maturity. An overview of the study design is illustrated in Fig 5b. For the project conducted in C57BL/6J wild-type mice, five different cohorts of mice (P25, P36, P50, P65, P120) were subjected to the test battery only once at a given developmental stage (S10 Fig). The test battery comprised a selection of home-cage based assessments, among others: nesting behavior, burrowing behavior, and the preference for a sweet saccharin solution. Moreover, voluntary wheel running performance and home-cage activity were assessed in C57BL/6J wild-type mice and the *Gria1* mouse model. An overview of the validated composite measure scheme for young mice [16–18] is provided in Fig 6. In addition to the set of parameters collected with the composite measure scheme, animals were subjected to daily clinical evaluations as requested by Directive 2010/63/EU. Clinical sum scores were calculated for the three adult epilepsy models and the genetic models (early adolescence and late adolescence, respectively). A detailed description of the clinical evaluation protocol is provided in the S1 Appendix.

Nesting behavior was assessed via nest pictures taken on a daily basis following a protocol by Jirkof and colleagues (2013) [77]. In addition, the mice’ ability and motivation to burrow food pellets were assessed at distinct time points following a slightly modified protocol by Deacon and colleagues (2006) [78]. The assessment of burrowing behavior included measurements during a two-hour time window in the light phase, followed by an analysis of overnight performance. The preference for a sweet solution can provide information on anhedonia-associated behavior [79], and we have previously validated the saccharin preference test as a behavioral assay for animal welfare assessments in three rat models of epilepsy [10–12]. In C57BL/6J wild-type mice and the genetic *Gria1* model, home-cage activity was assessed using freely accessible running wheels. Moreover, activity of the mice and the time mice spent in different zones of the cage (feeding zone, drinking zone, center zone) were analyzed for a preselected time window of 90 minutes, shortly after the beginning of the dark phase, when mice normally show highest levels of activity.

Locomotor and exploratory behaviors were investigated using the open field test. In addition, the elevated plus maze test and the black-white box test were applied to analyze anxiety-associated behavior in the adult models. In the adult epilepsy models, social interaction of the mice was tested in accordance with a protocol stated by File and Hyde (1978) [80]. Investigations in the adult models also included the mouse grimace scale, a well-validated assay for pain assessment in laboratory rodents [55]. As a classical neurobehavioral assay [56], Irwin scoring was applied in all models. The Irwin test can provide information on the general condition of the mice as well as on changes related to the vegetative, peripheral, and central nervous system [56]. The test represents a standard procedure in preclinical drug development phases and rodent safety pharmacology studies [81]. In C57BL/6J wild-type mice and in the genetic models, Irwin scoring was split into three consecutive parts with additional measurement of body temperature. Details on the Irwin scoring schemes are provided in the S1 Appendix. Concentrations of corticosterone metabolites measured in fecal samples of the mice can provide information on levels of distress [82] and metabolic needs [83]. Detailed information on the experimental procedures and settings as well as on the applied standardized protocols for data analyses, are provided in the S1 Appendix.

### Statistics

Statistical analyses and graphical illustrations were conducted using R version 4.1.2 [84]. To avoid their predominance, highly correlated parameters were excluded. In addition, parameters with more than 20% missing data were excluded. Spearman correlation was calculated, and the correlation matrix was visualized with the R package ‘corrplot’ [85]. All other graphical illustrations, including plots for principal component analyses (PCA), were created with ggplot2 [86]. To analyze the data from adult mouse models, data sets from the intrahippocampal kainate model, hippocampal-kindling model, and amygdala-kindling model were combined. Similarly, for the analysis of data from young mice, data sets from the *Scn1a* and the *Gria1* mouse models were combined. Data from young C57BL/6 wild-type mice were analyzed separately. Receiver operating characteristic (ROC) analyses were performed using the pROC package [87], testing for discriminatory power and predictive validity of selected behavioral tests. As described previously [5], data sets were subjected to a modified PCA procedure to identify top-ranking parameters and to cluster analyses based on a *k*-means algorithm. To identify top-ranking parameters, combined data sets from adult mice and combined data sets from young mice underwent a modified PCA procedure using the R package available at https://github.com/mytalbot/cms. First, data were subdivided into a ‘training set‘, comprising a random 80% of the data, and a ‘test set‘, comprising a random 20% of the data. In the following step, each parameter in the 80% ‘training set’ was centered and scaled by subtracting by its mean and dividing by its standard deviations, resulting in a mean of zero and a standard deviation of one, followed by a Box-Cox transformation. Finally, these data were used to perform the PCA. A composite score was calculated based on the ranked sums of preselected principal components, conducted by 100 runs of the ‘training data’. The number of principal components used for the composite score was based on the standard deviation in the PCA. Thereby, components with an SD > 1 were selected. Information on the ranked Eigenvalues calculated in each of the 100 iterations was used to identify top ranking parameters for preselected principal components. This modified PCA procedure allows the assessment of highly preserved features from any data set, and we have validated this technique previously [5].

For the calculation of robust clusters in the combined data sets from adult and young mice, a *k*-means based algorithm with cluster thresholds was determined by 100-fold resampling of the ‘training data’. Besides robust mean cluster thresholds, 95% confidence borders were calculated to detect potential variance instability due to random sampling.

## Supporting information

S1 Appendix (DOCX)

S1 Fig Correlation analysis (Spearman) for the three adult epilepsy models. The data set comprises the clinical evaluation (clinical score) and the set of behavioral/biochemical variables. The raw data underlying this figure are available in the Figshare repository https://doi.org/10.6084/m9.figshare.22759148.v1.
(PDF)

S2 Fig Results from one PCA run across the three adult epilepsy models. Animals from the intrahippocampal kainate model are highlighted as follows: individual data from animals with only one seizure are circled in grey. In comparison, data from animals that showed more than one seizure are circled in black. The raw data underlying this figure are available in the Figshare repository https://doi.org/10.6084/m9.figshare.22759148.v1.
(PDF)

S3 Fig Scree plot. Three adult epilepsy models. The raw data underlying this figure are available in the Figshare repository https://doi.org/10.6084/m9.figshare.22759148.v1.
(PDF)

S4 Fig Correlation analysis (Spearman). C57BL/6J model: adolescence (P25, P36, P50). The raw data underlying this figure are available in the Figshare repository https://doi.org/10.6084/m9.figshare.22759148.v1.
(PDF)

S5 Fig Correlation analysis (Spearman). C57BL/6J model: mature adult (P120). The raw data underlying this figure are available in the Figshare repository https://doi.org/10.6084/m9.figshare.22759148.v1.
(PDF)

S6 Fig Correlation analysis (Spearman) for the genetic models, early adolescence. The data set comprises the clinical evaluation (clinical score) and the set of behavioral/biochemical variables. The raw data underlying this figure are available in the Figshare repository https://doi.org/10.6084/m9.figshare.22759148.v1.
(PDF)

S7 Fig Correlation analysis (Spearman) for the genetic models, late adolescence. The data set comprises the clinical evaluation (clinical score) and the set of behavioral/biochemical variables. In both genetic models, clinical scores were not elevated during late adolescence (all animals were given a clinical score of zero in the assessment). The raw data underlying this figure are available in the Figshare repository https://doi.org/10.6084/m9.figshare.22759148.v1.
(PDF)

S8 Fig Scree plot. Genetic models: early adolescence. The raw data underlying this figure are available in the Figshare repository https://doi.org/10.6084/m9.figshare.22759148.v1.
(PDF)

S9 Fig Scree plot. Genetic models: late adolescence. The raw data underlying this figure are available in the Figshare repository https://doi.org/10.6084/m9.figshare.22759148.v1.
(PDF)

S10 Fig Overview of the experimental timeline of the C57BL/6J wild-type model. (PDF)

S1 Table Overview of the parameters (abbreviations) used for the correlation analyses. (PDF)

S2 Table a. p-values for correlation analysis (Spearman). Three adult epilepsy models. b. Correlation coefficients (r) for correlation analysis (Spearman). Three adult epilepsy models.
(ZIP)

S3 Table Top 30 parameters after 100 PCA runs of the training set in the three adult epilepsy models. (PDF)

S4 Table Results from the *k*-means cluster allocation in the three adult epilepsy models. (PDF)

S5 Table a. p-values for correlation analysis (Spearman). C57BL/6J model: adolescence (P25, P36, P50). b. Correlation coefficients (r) for correlation analysis (Spearman). C57BL/6J model: adolescence (P25, P36, P50).
(ZIP)

S6 Table. a. p-values for correlation analysis (Spearman). C57BL/6J model: P120. b. Correlation coefficients (r) for correlation analysis (Spearman). C57BL/6J model: P120 (ZIP)

S7 Table a. p-values for correlation analysis (Spearman). Genetic models: early adolescence. b. Correlation coefficients (r) for correlation analysis (Spearman). Genetic models: early adolescence.
(ZIP)

S8 Table a. p-values for correlation analysis (Spearman). Genetic models: late adolescence. b. Correlation coefficients (r) for correlation analysis (Spearman). Genetic models: late adolescence.
(ZIP)

S9 Table Top 30 parameters after 100 PCA runs of the training set. Genetic models.
(PDF)

S10 Table Results from the *k*-means cluster allocation in the genetic models. (PDF)

## References

[1] Russell WMS, Burch RL. The principles of humane experimental technique. Wheathampstead (UK): Universities Federation for Animal Welfare. 1959. (as reprinted 1992).

[2] Smith D, Anderson D, Degryse AD, Bol C, Criado A, Ferrara A, et al. Classification and reporting of severity experienced by animals used in scientific procedures: FELASA/ECLAM/ESLAV Working Group report. Lab Anim. 2018;52(1_suppl):5–57. doi: 10.1177/0023677217744587 29359995 PMC5987990

[3] Keubler LM, Hoppe N, Potschka H, Talbot SR, Vollmar B, Zechner D, et al. Where are we heading? Challenges in evidence-based severity assessment. Lab Anim. 2020;54(1):50–62. doi: 10.1177/0023677219877216 31718424

[4] Zintzsch A, Noe E, Reißmann M, Ullmann K, Krämer S, Jerchow B, et al. Guidelines on severity assessment and classification of genetically altered mouse and rat lines. Lab Anim. 2017;51(6):573–82. doi: 10.1177/0023677217718863 28696160

[5] van Dijk RM, Koska I, Bleich A, Tolba R, Seiffert I, Moller C, et al. Design of composite measure schemes for comparative severity assessment in animal-based neuroscience research: A case study focussed on rat epilepsy models. PLoS One. 2020;15(5):e0230141. doi: 10.1371/journal.pone.0230141 32413036 PMC7228039

[6] Gross D, Tolba RH. Ethics in Animal-Based Research. Eur Surg Res. 2015;55(1–2):43–57. doi: 10.1159/000377721 25871531

[7] Talbot SR, Biernot S, Bleich A, Van Dijk RM, Ernst L, Häger C, et al. Defining body-weight reduction as a humane endpoint: a critical appraisal. Lab Anim. 2020;54(1):99–110. doi: 10.1177/0023677219883319 31665969

[8] Tang G, Seume N, Häger C, Kumstel S, Abshagen K, Bleich A, et al. Comparing distress of mouse models for liver damage. Sci Rep. 2020;10(1):19814. doi: 10.1038/s41598-020-76391-w 33188220 PMC7666197

[9] Abdelrahman A, Kumstel S, Zhang X, Liebig M, Wendt EHU, Eichberg J, et al. A novel multi-parametric analysis of non-invasive methods to assess animal distress during chronic pancreatitis. Sci Rep. 2019;9(1):14084. doi: 10.1038/s41598-019-50682-3 31575986 PMC6773730

[10] Moller C, Wolf F, van Dijk RM, Di Liberto V, Russmann V, Keck M, et al. Toward evidence-based severity assessment in rat models with repeated seizures: I. Electrical kindling. Epilepsia. 2018;59(4):765–77. doi: 10.1111/epi.14028 29479675

[11] Koska I, van Dijk RM, Seiffert I, Di Liberto V, Möller C, Palme R, et al. Toward evidence-based severity assessment in rat models with repeated seizures: II. Chemical post-status epilepticus model. Epilepsia. 2019;60(10):2114–27. doi: 10.1111/epi.16330 31471910

[12] Seiffert I, van Dijk RM, Koska I, Di Liberto V, Moller C, Palme R, et al. Toward evidence-based severity assessment in rat models with repeated seizures: III. Electrical post-status epilepticus model. Epilepsia. 2019;60(8):1539–51. doi: 10.1111/epi.16095 31247135

[13] Moller C, van Dijk RM, Wolf F, Keck M, Schonhoff K, Bierling V, et al. Impact of repeated kindled seizures on heart rate rhythms, heart rate variability, and locomotor activity in rats. Epilepsy Behav. 2019;92:36–44. doi: 10.1016/j.yebeh.2018.11.034 30611006

[14] Boldt L, Koska I, Maarten van Dijk R, Talbot SR, Miljanovic N, Palme R, et al. Toward evidence-based severity assessment in mouse models with repeated seizures: I. Electrical kindling. Epilepsy Behav. 2021;115:107689. doi: 10.1016/j.yebeh.2020.107689 33418481

[15] Buchecker V, Koska I, Pace C, Talbot SR, Palme R, Bleich A, et al. Toward evidence-based severity assessment in mouse models with repeated seizures: (II.) Impact of surgery and intrahippocampal kainate. Eur Surg Res. 2022. doi: 10.1159/000522156 35073547 PMC9808668

[16] Reiber M, Koska I, Pace C, Schönhoff K, von Schumann L, Palme R, et al. Development of behavioral patterns in young C57BL/6J mice: a home cage-based study. Scientific Reports. 2022;12(1):2550. doi: 10.1038/s41598-022-06395-1 35169182 PMC8847349

[17] Reiber M, Miljanovic N, Schönhoff K, Palme R, Potschka H. Behavioral phenotyping of young *Scn1a* haploinsufficient mice. Epilepsy & Behavior. 2022;136:108903. 36240579 10.1016/j.yebeh.2022.108903

[18] Reiber M, Stirling H, Sprengel R, Gass P, Palme R, Potschka H. Phenotyping Young GluA1 Deficient Mice—A Behavioral Characterization in a Genetic Loss-of-Function Model. Frontiers in Behavioral Neuroscience. 2022;16. 10.3389/fnbeh.2022.877094 PMC9204703 35722188

[19] Brust V, Schindler PM, Lewejohann L. Lifetime development of behavioural phenotype in the house mouse (Mus musculus). Frontiers in Zoology. 2015;12(1):S17. doi: 10.1186/1742-9994-12-S1-S17 26816516 PMC4722345

[20] Sukoff Rizzo SJ, Crawley JN. Behavioral Phenotyping Assays for Genetic Mouse Models of Neurodevelopmental, Neurodegenerative, and Psychiatric Disorders. Annu Rev Anim Biosci. 2017;5:371–89. doi: 10.1146/annurev-animal-022516-022754 28199172

[21] Weegh N, Füner J, Janke O, Winter Y, Jung C, Struve B, et al. Wheel running behaviour in group-housed female mice indicates disturbed wellbeing due to DSS colitis. Lab Anim. 2020;54(1):63–72. doi: 10.1177/0023677219879455 31674858

[22] Kumstel S, Wendt EHU, Eichberg J, Talbot SR, Häger C, Zhang X, et al. Grading animal distress and side effects of therapies. Ann N Y Acad Sci. 2020;1473(1):20–34. doi: 10.1111/nyas.14338 32207155

[23] Häger C, Keubler LM, Talbot SR, Biernot S, Weegh N, Buchheister S, et al. Running in the wheel: Defining individual severity levels in mice. PLoS Biol. 2018;16(10):e2006159. doi: 10.1371/journal.pbio.2006159 30335759 PMC6193607

[24] Wassermann L, Helgers SOA, Riedesel AK, Talbot SR, Bleich A, Schwabe K, et al. Monitoring of Heart Rate and Activity Using Telemetry Allows Grading of Experimental Procedures Used in Neuroscientific Rat Models. Front Neurosci. 2020;14:587760. doi: 10.3389/fnins.2020.587760 33424534 PMC7793729

[25] Löscher W, Brandt C, Ebert U. Excessive weight gain in rats over extended kindling of the basolateral amygdala. Neuroreport. 2003;14(14):1829–32. doi: 10.1097/00001756-200310060-00014 14534429

[26] Jirkof P, Abdelrahman A, Bleich A, Durst M, Keubler L, Potschka H, et al. A safe bet? Inter-laboratory variability in behaviour-based severity assessment. Lab Anim. 2020;54(1):73–82. doi: 10.1177/0023677219881481 31696771

[27] Bleich A, Bankstahl M, Jirkof P, Prins JB, Tolba RH. Severity Assessment in animal based research. Lab Anim. 2020;54(1):16. doi: 10.1177/0023677219898105 32013756

[28] Zentrich E, Talbot SR, Bleich A, Häger C. Automated Home-Cage Monitoring During Acute Experimental Colitis in Mice. Front Neurosci. 2021;15:760606. doi: 10.3389/fnins.2021.760606 34744621 PMC8570043

[29] Baran SW, Bratcher N, Dennis J, Gaburro S, Karlsson EM, Maguire S, et al. Emerging Role of Translational Digital Biomarkers Within Home Cage Monitoring Technologies in Preclinical Drug Discovery and Development. Frontiers in Behavioral Neuroscience. 2022;15. doi: 10.3389/fnbeh.2021.758274 35242017 PMC8885444

[30] Voikar V, Gaburro S. Three Pillars of Automated Home-Cage Phenotyping of Mice: Novel Findings, Refinement, and Reproducibility Based on Literature and Experience. Front Behav Neurosci. 2020;14:575434. doi: 10.3389/fnbeh.2020.575434 33192366 PMC7662686

[31] Schollemann F, Barbosa Pereira C, Rosenhain S, Follmann A, Gremse F, Kiessling F, et al. An Anatomical Thermal 3D Model in Preclinical Research: Combining CT and Thermal Images. Sensors (Basel). 2021;21(4). doi: 10.3390/s21041200 33572091 PMC7915503

[32] Kunczik J, Barbosa Pereira C, Zieglowski L, Tolba R, Wassermann L, Häger C, et al. Remote vitals monitoring in rodents using video recordings. Biomed Opt Express. 2019;10(9):4422–36. doi: 10.1364/BOE.10.004422 31565499 PMC6757452

[33] Ahloy-Dallaire J, Klein JD, Davis JK, Garner JP. Automated monitoring of mouse feeding and body weight for continuous health assessment. Lab Anim. 2019;53(4):342–51. doi: 10.1177/0023677218797974 30286683

[34] Mallien AS, Häger C, Palme R, Talbot SR, Vogt MA, Pfeiffer N, et al. Systematic analysis of severity in a widely used cognitive depression model for mice. Lab Anim. 2020;54(1):40–9. doi: 10.1177/0023677219874831 31575329

[35] Weegh N, Zentrich E, Zechner D, Struve B, Wassermann L, Talbot SR, et al. Voluntary wheel running behaviour as a tool to assess the severity in a mouse pancreatic cancer model. PLoS One. 2021;16(12):e0261662. doi: 10.1371/journal.pone.0261662 34941923 PMC8699632

[36] Mallien AS, Pfeiffer N, Brandwein C, Inta D, Sprengel R, Palme R, et al. Comparative Severity Assessment of Genetic, Stress-Based, and Pharmacological Mouse Models of Depression. Front Behav Neurosci. 2022;16:908366. doi: 10.3389/fnbeh.2022.908366 35783227 PMC9245036

[37] Hohlbaum K, Bert B, Dietze S, Palme R, Fink H, Thöne-Reineke C. Impact of repeated anesthesia with ketamine and xylazine on the well-being of C57BL/6JRj mice. PLoS One. 2018;13(9):e0203559. doi: 10.1371/journal.pone.0203559 30231081 PMC6145541

[38] Hohlbaum K, Bert B, Dietze S, Palme R, Fink H, Thöne-Reineke C. Severity classification of repeated isoflurane anesthesia in C57BL/6JRj mice-Assessing the degree of distress. PLoS One. 2017;12(6):e0179588. doi: 10.1371/journal.pone.0179588 28617851 PMC5472303

[39] Hohlbaum K, Bert B, Dietze S, Palme R, Fink H, Thöne-Reineke C. Systematic Assessment of Well-Being in Mice for Procedures Using General Anesthesia. J Vis Exp. 2018(133). doi: 10.3791/57046 29630060 PMC5933230

[40] Bleich A, Tolba RH. How can we assess their suffering? German research consortium aims at defining a severity assessment framework for laboratory animals. Lab Anim. 2017;51(6):667. doi: 10.1177/0023677217733010 29160175

[41] Miljanovic N, Potschka H. The impact of Scn1a deficiency and ketogenic diet on the intestinal microbiome: A study in a genetic Dravet mouse model. Epilepsy Res. 2021;178:106826. doi: 10.1016/j.eplepsyres.2021.106826 34839144

[42] Miljanovic N, van Dijk RM, Buchecker V, Potschka H. Metabolomic signature of the Dravet syndrome: A genetic mouse model study. Epilepsia. 2021. doi: 10.1111/epi.16976 34223647

[43] Miljanovic N, Hauck SM, van Dijk RM, Di Liberto V, Rezaei A, Potschka H. Proteomic signature of the Dravet syndrome in the genetic Scn1a-A1783V mouse model. Neurobiol Dis. 2021;157:105423. doi: 10.1016/j.nbd.2021.105423 34144125

[44] Zamanillo D, Sprengel R, Hvalby O, Jensen V, Burnashev N, Rozov A, et al. Importance of AMPA receptors for hippocampal synaptic plasticity but not for spatial learning. Science. 1999;284(5421):1805–11. doi: 10.1126/science.284.5421.1805 10364547

[45] Vogt MA, Elkin H, Pfeiffer N, Sprengel R, Gass P, Inta D. Impact of adolescent GluA1 AMPA receptor ablation in forebrain excitatory neurons on behavioural correlates of mood disorders. Eur Arch Psychiatry Clin Neurosci. 2014;264(7):625–9. doi: 10.1007/s00406-014-0509-5 24895223

[46] Inta D, Vogt MA, Elkin H, Weber T, Lima-Ojeda JM, Schneider M, et al. Phenotype of mice with inducible ablation of GluA1 AMPA receptors during late adolescence: relevance for mental disorders. Hippocampus. 2014;24(4):424–35. doi: 10.1002/hipo.22236 24339333

[47] Ben Abdallah NM, Fuss J, Trusel M, Galsworthy MJ, Bobsin K, Colacicco G, et al. The puzzle box as a simple and efficient behavioral test for exploring impairments of general cognition and executive functions in mouse models of schizophrenia. Exp Neurol. 2011;227(1):42–52. doi: 10.1016/j.expneurol.2010.09.008 20851119

[48] Bailoo JD, Voelkl B, Varholick J, Novak J, Murphy E, Rosso M, et al. Effects of weaning age and housing conditions on phenotypic differences in mice. Sci Rep. 2020;10(1):11684. doi: 10.1038/s41598-020-68549-3 32669633 PMC7363894

[49] Eltokhi A, Kurpiers B, Pitzer C. Baseline Depression-Like Behaviors in Wild-Type Adolescent Mice Are Strain and Age but Not Sex Dependent. Front Behav Neurosci. 2021;15:759574. doi: 10.3389/fnbeh.2021.759574 34690714 PMC8529326

[50] Eltokhi A, Kurpiers B, Pitzer C. Behavioral tests assessing neuropsychiatric phenotypes in adolescent mice reveal strain- and sex-specific effects. Sci Rep. 2020;10(1):11263. doi: 10.1038/s41598-020-67758-0 32647155 PMC7347854

[51] Dravet C. The core Dravet syndrome phenotype. Epilepsia. 2011;52 Suppl 2:3–9. doi: 10.1111/j.1528-1167.2011.02994.x 21463272

[52] Brunklaus A, Dorris L, Zuberi SM. Comorbidities and predictors of health-related quality of life in Dravet syndrome. Epilepsia. 2011;52(8):1476–82. doi: 10.1111/j.1528-1167.2011.03129.x 21668444

[53] Villas N, Meskis MA, Goodliffe S. Dravet syndrome: Characteristics, comorbidities, and caregiver concerns. Epilepsy Behav. 2017;74:81–6. doi: 10.1016/j.yebeh.2017.06.031 28732259

[54] Lagae L, Brambilla I, Mingorance A, Gibson E, Battersby A. Quality of life and comorbidities associated with Dravet syndrome severity: a multinational cohort survey. Dev Med Child Neurol. 2018;60(1):63–72. doi: 10.1111/dmcn.13591 28984349

[55] Langford DJ, Bailey AL, Chanda ML, Clarke SE, Drummond TE, Echols S, et al. Coding of facial expressions of pain in the laboratory mouse. Nat Methods. 2010;7(6):447–9. doi: 10.1038/nmeth.1455 20453868

[56] Irwin S. Comprehensive observational assessment: Ia. A systematic, quantitative procedure for assessing the behavioral and physiologic state of the mouse. Psychopharmacologia. 1968;13(3):222–57. doi: 10.1007/BF00401402 5679627

[57] Jafari M, Soerensen J, Bogdanović RM, Dimou L, Götz M, Potschka H. Long-term genetic fate mapping of adult generated neurons in a mouse temporal lobe epilepsy model. Neurobiol Dis. 2012;48(3):454–63. doi: 10.1016/j.nbd.2012.06.014 22750527

[58] von Rüden EL, Wolf F, Gualtieri F, Keck M, Hunt CR, Pandita TK, et al. Genetic and Pharmacological Targeting of Heat Shock Protein 70 in the Mouse Amygdala-Kindling Model. ACS Chem Neurosci. 2019;10(3):1434–44. doi: 10.1021/acschemneuro.8b00475 30396268

[59] Groticke I, Hoffmann K, Loscher W. Behavioral alterations in a mouse model of temporal lobe epilepsy induced by intrahippocampal injection of kainate. Exp Neurol. 2008;213(1):71–83. doi: 10.1016/j.expneurol.2008.04.036 18585709

[60] Miller AR, Hawkins NA, McCollom CE, Kearney JA. Mapping genetic modifiers of survival in a mouse model of Dravet syndrome. Genes Brain Behav. 2014;13(2):163–72. doi: 10.1111/gbb.12099 24152123 PMC3930200

[61] Shmuely S, Sisodiya SM, Gunning WB, Sander JW, Thijs RD. Mortality in Dravet syndrome: A review. Epilepsy Behav. 2016;64(Pt A):69–74. doi: 10.1016/j.yebeh.2016.09.007 27732919

[62] Wallace A, Wirrell E, Kenney-Jung DL. Pharmacotherapy for Dravet Syndrome. Paediatr Drugs. 2016;18(3):197–208. doi: 10.1007/s40272-016-0171-7 26966048

[63] Chiron C, Dulac O. The pharmacologic treatment of Dravet syndrome. Epilepsia. 2011;52 Suppl 2:72–5. doi: 10.1111/j.1528-1167.2011.03007.x 21463285

[64] Kuo FS, Cleary CM, LoTurco JJ, Chen X, Mulkey DK. Disordered breathing in a mouse model of Dravet syndrome. Elife. 2019;8. doi: 10.7554/eLife.43387 31025941 PMC6506208

[65] Ricobaraza A, Mora-Jimenez L, Puerta E, Sanchez-Carpintero R, Mingorance A, Artieda J, et al. Epilepsy and neuropsychiatric comorbidities in mice carrying a recurrent Dravet syndrome SCN1A missense mutation. Sci Rep. 2019;9(1):14172. doi: 10.1038/s41598-019-50627-w 31578435 PMC6775062

[66] Tang SH, Silva FJ, Tsark WM, Mann JR. A Cre/loxP-deleter transgenic line in mouse strain 129S1/SvImJ. Genesis. 2002;32(3):199–202. doi: 10.1002/gene.10030 11892008

[67] Fitzgerald PJ, Barkus C, Feyder M, Wiedholz LM, Chen YC, Karlsson RM, et al. Does gene deletion of AMPA GluA1 phenocopy features of schizoaffective disorder? Neurobiol Dis. 2010;40(3):608–21. doi: 10.1016/j.nbd.2010.08.005 20699120 PMC2955784

[68] Wiedholz LM, Owens WA, Horton RE, Feyder M, Karlsson RM, Hefner K, et al. Mice lacking the AMPA GluR1 receptor exhibit striatal hyperdopaminergia and ’schizophrenia-related’ behaviors. Mol Psychiatry. 2008;13(6):631–40. doi: 10.1038/sj.mp.4002056 17684498

[69] Barkus C, Feyder M, Graybeal C, Wright T, Wiedholz L, Izquierdo A, et al. Do GluA1 knockout mice exhibit behavioral abnormalities relevant to the negative or cognitive symptoms of schizophrenia and schizoaffective disorder? Neuropharmacology. 2012;62(3):1263–72. doi: 10.1016/j.neuropharm.2011.06.005 21693126 PMC3208051

[70] Sanacora G, Treccani G, Popoli M. Towards a glutamate hypothesis of depression: an emerging frontier of neuropsychopharmacology for mood disorders. Neuropharmacology. 2012;62(1):63–77. doi: 10.1016/j.neuropharm.2011.07.036 21827775 PMC3205453

[71] Reisel D, Bannerman DM, Schmitt WB, Deacon RM, Flint J, Borchardt T, et al. Spatial memory dissociations in mice lacking GluR1. Nat Neurosci. 2002;5(9):868–73. doi: 10.1038/nn910 12195431

[72] Schmitt WB, Deacon RM, Seeburg PH, Rawlins JN, Bannerman DM. A within-subjects, within-task demonstration of intact spatial reference memory and impaired spatial working memory in glutamate receptor-A-deficient mice. J Neurosci. 2003;23(9):3953–9. doi: 10.1523/JNEUROSCI.23-09-03953.2003 12736365 PMC6742186

[73] Bannerman DM, Deacon RM, Brady S, Bruce A, Sprengel R, Seeburg PH, et al. A comparison of GluR-A-deficient and wild-type mice on a test battery assessing sensorimotor, affective, and cognitive behaviors. Behav Neurosci. 2004;118(3):643–7. doi: 10.1037/0735-7044.118.3.643 15174943

[74] Vekovischeva OY, Zamanillo D, Echenko O, Seppälä T, Uusi-Oukari M, Honkanen A, et al. Morphine-induced dependence and sensitization are altered in mice deficient in AMPA-type glutamate receptor-A subunits. J Neurosci. 2001;21(12):4451–9. doi: 10.1523/JNEUROSCI.21-12-04451.2001 11404432 PMC6762742

[75] Chourbaji S, Vogt MA, Fumagalli F, Sohr R, Frasca A, Brandwein C, et al. AMPA receptor subunit 1 (GluR-A) knockout mice model the glutamate hypothesis of depression. Faseb j. 2008;22(9):3129–34. doi: 10.1096/fj.08-106450 18492725

[76] Austen JM, Sprengel R, Sanderson DJ. GluA1 AMPAR subunit deletion reduces the hedonic response to sucrose but leaves satiety and conditioned responses intact. Sci Rep. 2017;7(1):7424. doi: 10.1038/s41598-017-07542-9 28785046 PMC5547105

[77] Jirkof P, Fleischmann T, Cesarovic N, Rettich A, Vogel J, Arras M. Assessment of postsurgical distress and pain in laboratory mice by nest complexity scoring. Lab Anim. 2013;47(3):153–61. doi: 10.1177/0023677213475603 23563122

[78] Deacon RM. Burrowing in rodents: a sensitive method for detecting behavioral dysfunction. Nat Protoc. 2006;1(1):118–21. doi: 10.1038/nprot.2006.19 17406222

[79] Klein S, Bankstahl JP, Loscher W, Bankstahl M. Sucrose consumption test reveals pharmacoresistant depression-associated behavior in two mouse models of temporal lobe epilepsy. Exp Neurol. 2015;263:263–71. doi: 10.1016/j.expneurol.2014.09.004 25220610

[80] File SE, Hyde JR. Can social interaction be used to measure anxiety? Br J Pharmacol. 1978;62(1):19–24. doi: 10.1111/j.1476-5381.1978.tb07001.x 563752 PMC1667770

[81] Lynch JJ 3rd, Castagné V, Moser PC, Mittelstadt SW. Comparison of methods for the assessment of locomotor activity in rodent safety pharmacology studies. J Pharmacol Toxicol Methods. 2011;64(1):74–80. doi: 10.1016/j.vascn.2011.03.003 21406241

[82] Touma C, Palme R, Sachser N. Analyzing corticosterone metabolites in fecal samples of mice: a noninvasive technique to monitor stress hormones. Horm Behav. 2004;45(1):10–22. doi: 10.1016/j.yhbeh.2003.07.002 14733887

[83] Koolhaas JM, Bartolomucci A, Buwalda B, de Boer SF, Flügge G, Korte SM, et al. Stress revisited: a critical evaluation of the stress concept. Neurosci Biobehav Rev. 2011;35(5):1291–301. doi: 10.1016/j.neubiorev.2011.02.003 21316391

[84] R Core Team. R: A language and environment for statistical computing. R Foundation for Statistical Computing, Vienne, Austria. 2020.

[85] Wei T, Simko V. R package "corrplot": Visualization of a Correlation Matrix. (Version 0.9.2), https://github.com/taiyun/corrplot. 2021.

[86] Wickham H. ggplot2: Elegant Graphics for Data Analysis. Springer-Verlag New York. ISBN 978-3-319-24277-4, https://ggplot2.tidyverse.org. 2016.

[87] Robin X, Turck N, Hainard A, Tiberti N, Lisacek F, Sanchez JC, et al. pROC: an open-source package for R and S+ to analyze and compare ROC curves. BMC Bioinformatics. 2011;12:77. doi: 10.1186/1471-2105-12-77 21414208 PMC3068975

