# Supplementary material for: Evidence-based comparative severity assessment in young and adult mice
Source: PLoS One. 2023 Oct 20;18(10):e0285429. doi: 10.1371/journal.pone.0285429 (PMC10588901; doi:10.1371/journal.pone.0285429)
Supplement: S1 Appendix — (DOCX) [file pone.0285429.s001.docx]

**Evidence-based comparative severity assessment in young and adult mice**

Maria Reiber, Lara von Schumann, Verena Buchecker, Lena Boldt, Peter Gass, Andre Bleich, Steven Roger Talbot and Heidrun Potschka

**^Supplementary Methods^**

1. **^Animal models^**
2. **^Behavioral and biochemical analysis^**
3. **^Clinical evaluation protocol^**

**^Supplementary References^**

***Supplementary Methods***

1. ***Animal models***
   1. *Adult mice*

*Housing*

Animals were single-housed with freely available tap water and food (ssniff® R/M-H, Sniff, Soest, Germany). The animal facility's environmental conditions were kept at standard settings (temperature 20-24°C, humidity 45-65%, regular 12 h light/dark cycle with lights on during the day). Mice were kept in open standard Makrolon type III cages (Zoonlab, Castrop-Rauxel, Germany), which were supplied with fresh wood chip bedding material (Lignocel Select, J. Rettenmaier & Söhne GmbH & Co. KG, Rosenberg, Germany), two nestlets (Ancare, Bellmore, New York, USA) and one mouse house (Zoonlab, Castrop – Rauxel, Germany) per cage weekly.

*Randomization and Blinding*

In both projects, animals were randomly assigned to the cages, and cages were placed in random order. The allocation to the experimental groups was randomized based on the results of the burrowing baseline test. The order of animals was randomized for each of the behavioral tests.

Experimenters and animal caregivers were blinded. However, blinding was impossible for the naïve animals because they did not have implanted electrodes. In the kindling model, the experimenter was blinded to the location of the electrode (amygdala or hippocampus). No blinding was applied for the mouse grimace scale (MGS) and the Irwin test. Image-based analysis of nesting behavior was conducted by a person blinded for group allocation. Manual scorings of parameters from the open field test, the black-and-white box test, the elevated plus maze test, and the social interaction test were also executed by a person blinded for group allocation. However, blinding was not applicable for the manual scoring of naïve animals.

*Intrahippocampal-kainate model (for the original publication, please see Ref (1))*

Female mice (n =36) of the strain HsdWin:NMRI (Envigo, Horst, Netherlands) were used for the study at the age of four to five weeks. The weight of the mice was 20 to 22 g at arrival. They were allocated to four treatment groups: one naïve group (n = 8) without any surgical intervention, one group receiving only a telemetric transmitter but no electrode implantation (n = 8), a sham group with transmitter and electrode implantation (n = 8) and a treatment group with transmitter and electrode implants and kainate administration (n = 12). For better comparability, the eight animals from the telemetric-only group were not included in the analyses. Therefore, in total, data from 28 animals were used.

*Kindling model (for the original publication, please see Ref (2))*

In total, 80 female mice (HsdWin:NMRI, Envigo, Horst, Netherlands) were used. At arrival, mice were ten weeks old and had a body weight of 25-30 g. Animals were randomly allocated to five groups: one naïve without any surgical intervention (n = 15), two sham groups, receiving electrodes either in the hippocampus or the amygdala (n = 15 each), and two experimental groups, receiving electrodes either in the hippocampus or the amygdala (n = 15 each). Electrode-implanted mice from the experimental groups were kindled on five days a week. The project was divided into two sub-projects to analyze both implantation sites individually, and the naïve group was used as a control group for both experimental groups. Therefore, ten animals had to be excluded from the experiment. Details on exclusion criteria are provided in the original publication.

*1.2 Young mice*

*1.2.1 C57BL/6J wild-type mice (for the original publication, please see Ref (3))*

*Breeding and housing*

As described previously (3), pregnant, time-mated C57BL/6JRj mice (n=51) were obtained at embryonic stages (E) 10, E11, and E13 from Janvier Labs (Le Genest-Saint-Isle, France). Pregnant mice were single-housed under controlled environmental conditions (22-24 °C, 45-60% humidity) in individually ventilated cages (Tecniplast Deutschland GmbH, Hohenpeißenberg, Germany) in a 12-h dark-light cycle with ad libitum access to food (Ssniff Spezialdiäten GmbH, Soest, Germany) and tap water at the Institute of Pharmacology, LMU Munich. Cages were supplemented with nesting material (Enviro Dri, Claus GmbH, Limburgerhof, Germany), a wood brick (Labodia AG, Niederglatt, Switzerland), and a triangular mouse house (**Zoonlab GmbH,** Castrop-Rauxel, Germany). Each mother with litter obtained a fresh cage on postnatal day (P) 6 and 14. Offspring animals were weaned at P21. From weaning onwards, offspring mice (n=200, female:male = 1:1) were housed per experimental unit (n=2). One experimental unit consisted of same-sex siblings. In cases with more than two same-sex siblings available, group allocation was determined randomly (www.randomizer.org). Experimental units (n=100) were housed in Makrolon cages type III (Ehret GmbH & Co. KG, Emmendingen, Germany), provided with bedding material (Lignocel Select, J. Rettenmaier & Söhne GmbH & Co. KG, Rosenberg, Germany), two nestlets (Ancare, Bellmore, New York, USA), and one square animal house (**Zoonlab GmbH,** Castrop-Rauxel, Germany). The order of cages was determined randomly (www.randomizer.org). Mice received fresh cages once a week on Tuesdays.

During the experimental phase, mice were housed as experimental units (n=2) in PhenoTyper cages (Noldus), supplemented with 200 g bedding material (Lignocel Select)**,** two nestlets (Ancare, Bellmore, New York, USA), an infrared translucent shelter (Noldus) and two drinking bottles (Noldus). The home cage system comprised a removable wall for inserting a running wheel (PhenoWheel, Noldus).

*1.2.2 Scn1a mouse model (for the original publication, please see the Ref (4))*

*Breeding and housing*

As described previously (4), experimental animals were bred at the Institute of Pharmacology, Toxicology, and Pharmacy, LMU Munich: The breeding colony, comprising the parental lines B6(Cg)-*Scn1a^tm1.1Dsf^* /J (5, 6) (JAX stock #026133) and 129S1/Sv-*Hprt^tm1(CAG-cre)Mnn^* /J (7) (JAX stock #004302), was maintained at our Institute. Experimental animals (n=40) were bred by mating 12 female mice of the strain 129S1/Sv-*Hprt^tm1(CAG-cre)Mnn^* /J, heterozygous for Cre recombinase, with six male mice of the strain B6(Cg)-*Scn1a^tm1.1Dsf^* /J, which represent a conditional knock-in model with a floxed *Scn1a* gene, expressing the mutation A1783V in exon 26.

Pregnant mice with litters were housed individually under controlled environmental conditions (22-24 °C, 45-60% humidity) in individually ventilated cages (Tecniplast Deutschland GmbH, Hohenpeißenberg, Germany) in a 12-hour dark-light cycle with ad libitum access to food (Ssniff Spezialdiäten GmbH, Soest, Germany) and tap water. Cages were enriched with bedding material (Lignocel Select, J. Rettenmaier & Söhne GmbH & Co. KG, Rosenberg, Germany), nesting material (Enviro Dri, Claus GmbH, Limburgerhof, Germany), a wood brick (Labodia AG, Niederglatt, Switzerland), and a triangular mouse house (**Zoonlab GmbH,** Castrop-Rauxel, Germany). Genotyping was conducted with weaning at P19 between 5:00 and 7:00 p.m. Sampling for a second PCR to confirm the genotype of the mice was carried out after completion of the experiments.

Following weaning, mice were housed in sex-matched groups of four to six animals per cage in Makrolon type III cages (Ehret GmbH & Co. KG, Emmendingen, Germany), supplemented with bedding material (Lignocel Select, J. Rettenmaier & Söhne GmbH & Co. KG, Rosenberg, Germany), Enviro Dri nesting material (Enviro Dri, Claus GmbH, Limburgerhof, Germany), two nestlets (Ancare, Bellmore, New York, USA), and one square animal house (**Zoonlab GmbH,** Castrop-Rauxel, Germany).

From P26 onward, mice were housed in groups of two per experimental unit, according to their *Scn1a*-genotype and sex. Whenever possible, littermates were used for the same experimental unit. In cases when this was not possible, the group allocation of experimental units was determined randomly (www.randomizer.org).

During four days each, in the developmental stages of prepubescence and sexual maturity, experimental units (n=20) were housed in PhenoTyper cages (Noldus, Wageningen, the Netherlands). Data analysis was done using the video analysis tracking software EthoVision XT 15 (EthoVision XT, RRID:SCR_000441). Each PhenoTyper cage was supplemented with 200 g bedding material (Lignocel Select, J. Rettenmaier & Söhne GmbH & Co. KG, Rosenberg, Germany), two nestlets (Ancare, Bellmore, New York, USA), an infrared translucent shelter (Noldus, Wageningen, the Netherlands) and two drinking bottles (Noldus, Wageningen, the Netherlands).

*Intense care measures*

As described previously (4), offspring mice aged 14 to 26 days received a cup of Dietgel76A (Ssniff Spezialdiäten GmbH, Soest, Germany) per cage on a daily basis, stirred up with 5-7 ml of 10 % glucose in tap water. The days following weaning at P19 are referred to a ‘period of intense care’. Since we have previously observed high mortality rates during this time slot (8), offspring mice were offered special nutrition and critical care, in particular: from P19 to P26, offspring mice received wet, sweetened food pellets on the cage ground and a cup of Dietgel76A with 5-7 ml of glucose 10 % in tap water twice a day. Weak mice and mice which seemed to face difficulties with spatial orientation were additionally fed by hand with glucose 10% and Dietgel76A, mixed and drawn up in a syringe. Feeding intervals of approximately three hours were prolonged as soon as the mice continuously fed on their own. Noises, fixation of the animals, and all other disturbing factors were strictly avoided.

*1.2.3 Gria1 mouse model (for the original publication, please see Ref (9))*

*Breeding and housing*

As described previously (9), experimental animals were bred at the Institute of Pharmacology, Toxicology and Pharmacy, LMU Munich, and breeding animals were obtained from the animal facility of Heidelberg University (IBF, Heidelberg). *Gria1* knockout (*Gria1-/-)* mice (line B6N.129-*Gria1^tm1Rsp^*/J, available at The Jackson Laboratory: Strain #019011), Mouse Genome Informatics ID: MGI:2178057) had originally been generated as described previously (10). For colony breeding at the IBF Heidelberg, *Gria1-/-* mice had been backcrossed into C57BL/6N for more than 10 generations. For experimental breeding at our Institute, 19 female *Gria1+/-* and 7 male *Gria1+/-*, obtained from the IBF Heidelberg, were mated (heterozygous (HET) x HET breeding scheme). The resulting offspring mice harboring the *Gria1+/+*, *Gria1+/-,* and *Gria1-/-* gene, were genotyped before weaning at P21 by ear-punch biopsy as described previously (10). Additional PCRs confirmed *Gria1* genotypes after completion of the experiments shortly before euthanasia.

Breeding animals were housed in Makrolon type III cages (Ehret GmbH & Co. KG, Emmendingen, Germany), enriched with bedding material (Lignocel Select, J. Rettenmaier & Söhne GmbH & Co. KG, Rosenberg, Germany), nesting material (Enviro-Dri, Claus GmbH, Limburgerhof, Germany), two nestlets (Ancare, Bellmore, New York, USA), and one square animal house (**Zoonlab GmbH,** Castrop-Rauxel, Germany).

All offspring animals were weaned at P21. From weaning onwards, experimental mice (n=38) harboring the *Gria1+/+* wild-type gene (referred to as ‘*Gria1* WT’ in the following) or the *Gria1* knockout variant (referred to as ‘Gria1-/-’ in the following) were housed in same-genotype-same-sex groups of two as one experimental unit (n=19). Whenever possible, littermates were used for the same experimental unit. In cases when this was not possible, the group allocation of experimental units was determined randomly (www.randomizer.org). Experimental units, comprising 19 units with 2 mice each, were housed under controlled environmental conditions (22 – 24°C, 45 – 60% humidity) in a 12-hour dark-light cycle with *ad libitum* access to food (Ssniff Spezialdiäten GmbH, Soest, Germany) and tap water, in Makrolon type III cages (Ehret GmbH & Co. KG, Emmendingen, Germany), provided with bedding material (Lignocel Select, J. Rettenmaier & Söhne GmbH & Co. KG, Rosenberg, Germany), two nestlets (Ancare, Bellmore, New York, USA) and one square animal house (**Zoonlab GmbH,** Castrop-Rauxel, Germany) per cage. The order of cages was randomized (www.randomizer.org).

During four days each, at a prepubescent and a sexually mature age in adolescence, experimental units (n=19) were housed in home-cage systems with continuous video recording (PhenoTyper cage, Noldus, Wageningen, the Netherlands), combined with the software EthoVision XT 15 (EthoVision XT, RRID:SCR_000441) for video-based tracking analysis. In addition, each PhenoTyper cage was supplemented with 200 g bedding material (Lignocel Select, J. Rettenmaier & Söhne GmbH & Co. KG, Rosenberg, Germany), two nestlets (Ancare, Bellmore, New York, USA), an infrared translucent shelter (Noldus, Wageningen, the Netherlands), and two drinking bottles (Noldus, Wageningen, the Netherlands).

1. ***Behavioral and biochemical analysis***

*2.1 Nest building*

Nest building behavior was assessed as described previously in a protocol by Jirkof and colleagues (2013) (11). In brief: nest pictures were taken between 5:00 a.m. and 8:00 a.m. (CEST) and 6:00 a.m. and 9:00 a.m. (CEWT) on a daily basis. Based on these pictures, nest complexity was scored by a person blinded for group allocation. The scoring system was based on a standard operating protocol of the DFG research group FOR 2591:

Score 1 - the nestlet is almost untouched, > 90 % are intact;

Score 2 - the nestlet is partially ruptured, 50 – 90 % are still intact;

Score 3 - the nestlet is mostly destroyed, 50 – 90 % of the nestlet is destroyed, < 50 % of the nestlet is intact, < 90 % of the nestlet is in one-quarter of the cage area; the cotton is not formed to a nest but distributed in the cage;

Score 4 - an identifiable, flat nest, > 90 % of the nestlet used for the nest, the material has the shape of a nest and is located in one-quarter of the cage area, the nest is flat i.e. less than 50 % of the circumference of the nest wall is higher than one-third of the height of the mouse house; Score 5 - an almost perfect nest, > 90 % of the nestlet used for the nest, the nest resembles a crater with more than 50 % of the circumference of the nest wall higher than one-third of the height of the mouse house;

Score 6 - perfect nest, > 90 % of the nestlet used for the nest, the nest resembles a crater with more than 90 % of the circumference of the nest wall higher than one-third of the height of the mouse house.

For the analyses of data from the three adult epilepsy models, readouts obtained from one day in the chronic phase (intrahippocampal kainate model) and from one day in the phase of generalized seizures (kindling models) were used. For the analyses of data from the two genetic models and in the C57BL/6J wild-type mice, sum scores created from the sum of four consecutive days (genetic models) and six consecutive days (C57BL/6J wild-type mice) were used.

*2.2 Saccharin preference*

The saccharin preference test was performed as described in a protocol by Klein and colleagues (2015) (12). 200 g of liquid was provided in each bottle with a watering nipple with a diameter of 1 mm. To avoid side preference effects, two water bottles were attached to the cage during the entire study. The test was carried out on four consecutive days. On the first day, the water intake was evaluated. For that purpose, both bottles were filled with 200 g of tap water each. On the following day, one of the two bottles was filled with 200 g of a 0.1 % saccharin solution, while the other bottle contained 200 g of tap water (Aldrich Saccharin ≥ 98%, Sigma-Aldrich Chemie GmbH, Germany) to assess the preference for the sweet solution. On day three of the experiment, both bottles were filled again with tap water. On the fourth day, one of the two bottles was filled with a 0.1 % saccharin solution, while the other contained tap water. The position of the bottle containing the saccharin solution was alternated on day 2 and day 4 to avoid side effects. The consumed amounts of liquid were assessed after 24 hours for each testing day.

*2.3 Burrowing*

The assessment of burrowing performance was based on a protocol published by Deacon and colleagues (13). The procedure had been slightly adjusted for the development of a protocol used by the research group FOR 2591.

As described previously (1-4, 9), bottles (Zoonlab, Germany) with a length of 20 cm and an opening of 3.5 cm diameter were filled with 200 +/- 1 g food pellets (Ssniff, Spezialdiäten GmbH, Soest, Germany). Two hours before the dark phase, a filled bottle was placed in the corner of each animal's home cage, with the closed end facing the cage wall. After two hours, the bottle with the remaining pellets was removed from the cage and weighed. Afterward, the bottle with the remaining pellets was returned to the cage. At the end of the dark phase, the bottle was weighed again to evaluate the burrowed amount of pellets during the dark phase.

For the investigations in adult mice, the burrowing test was performed at four time points throughout the studies. For the analysis in the present study, only the data from the fourth timepoint in the chronic phase (intrahippocampal kainate model) and phase of generalized seizures (kindling models) of the projects were considered.

For the investigations conducted in the two genetic *loss-of-function* mouse models (4, 9), the burrowing test was applied twice on two consecutive days during late adolescence. For the data generated in C57BL/6J wild-type mice (3), the burrowing test was applied twice on two consecutive days for each age group separately. For the analyses in the present study, the mean was calculated from the data of the first and the second day of burrowing assessment. Values from the two consecutive days were combined since no relevant difference was observed when comparing the respective performance between the two days. Combined values were created for burrowing performance in the dark and light phases, respectively.

*2.4 Voluntary wheel running*

Voluntary wheel running performance was assessed over four days in the PhenoTyper cages (Noldus) using the tracking software Ethovision XT15 (Noldus, RRID:SCR_000441). Running wheels (diameter: 15 cm, width 7 cm) were freely accessible for the mice 24 hours per day. The analyses were based on the rotations of the wheel automatically assessed per minute.

*2.5 Home-cage activity*

Activity patterns were assessed by bench-top assessment (14) in the PhenoTyper cages (Noldus), which were utilized as home cages for the observation periods of at least four days. As described previously (9), tracking-based analyses (Ethovision XT 15) were applied on the third day after the mice had been introduced to the PhenoTyper cages. Among the parameters analyzed, we assessed the distance and velocity of the mice during a time slot of precisely 60 minutes shortly after the beginning of the dark phase. In addition, the time mice spent in three zones of the PhenoTyper cages was analyzed: 1) the area surrounding the feeder rack (zone ‘feeding’), 2) the area surrounding the drinking bottles (zone ‘drinking’), and 3) the center of the cage (zone ‘center’).

*2.6 Open field test*

The open field test was used to examine exploratory behavior and locomotor activity. The light intensity was set at approximately 12-20 lux. Two cylinders (60 cm diameter, 40 cm height) were used simultaneously to perform two runs in parallel. After each trial, open fields were cleaned with 0.1% acetic acid. To obtain ideal detection settings for tracking-based analyses (color contrast between the animal and the environmental setting), the color of the cylinders depended on the fur color of the mice: For all investigations performed in adult mice, black arenas were used, for the investigations in young mice, white arenas were used. Mice were placed individually into the open field arena, facing the wall at a distance of 10 cm. Animals were video recorded (CCTV Camera, Panasonic, Suzhou, China) for ten minutes (investigations in adult mice) or 15 minutes (investigations in young mice) using EthoVision XT software v. 8.5 Software (Noldus, RRID:SCR_000441). The software tracked the total distance moved by the mice, the time of immobility, and the time mice spent in the different zones of the arena (wall, middle, center). In addition, rearing behavior and ‘jumps’ from the mice against the arena wall were scored manually by a person unaware of group allocation.

*2.7 Irwin Score*

Classical Irwin Scoring is a neurobehavioral assay that can provide additional information on the general condition of the mice, and on behavioral changes related to the mice's vegetative, peripheral and central nervous system (15). In the Irwin test, a total number of 33 parameters were assessed individually. In addition, for the investigations in young mice, the occurrence of seizures was assessed as an additional parameter. Based on the results from the single parameters, a sum score was calculated. For the investigations in young mice, as described previously (3, 4, 9), we applied a slightly modified Irwin scoring system divided into three consecutive parts. Irwin scoring was carried out from the least invasive parameters assessed in the home cage to the more interfering, handling-associated parameters: first, observation in the PhenoTyper cages, second, observation during the open field test, and third, observation and handling in fresh Makrolon type II open cages (Ehret GmbH & Co. KG, Emmendingen, Germany) enriched with bedding material (Lignocel Select, J. Rettenmaier & Söhne GmbH & Co. KG, Rosenberg, Germany).

An overview of the parameters assessed in the Irwin test is provided below.

| 1 Body position  2 Ptosis  3 Locomotor activity  4 Touch-response  5 Grasp-irritability  6 Curiosity (showing object)  7 Provoked freezing  8 Vocalization  9 Urination (while handling)  10 Defecation (while handling)  11 Lid reflex  12 Corneal reflex  13 Startle  14 Pelvic elevation  15 Tail elevation  16 Limb rotation  17 Body tone (while handling) | 18 Abdominal tone  19 Righting reflex  20 Ataxia  21 Exophthalmos  22 Hypersalivation  23 Lacrimation  24 Feces  25 Piloerection  26 Skin perfusion  27 Respiratory rate  28 Tremors  29 Twitches  30 Convulsions  31 Area of implant  32 Stereotypies  33 Body temperature  34 Seizures |
| --- | --- |

Details on the applied scoring schemes are provided in the original publications.

*2.8 Mouse grimace scale (MGS)*

For the MGS assessments, we applied a protocol developed by Langford and colleagues (2016). Five parameters were assessed, and a sum score was calculated (16). Individual parameters assessed are listed below.

1. Orbital tightening
2. Nose bulge
3. Cheek bulge
4. Ear position
5. Whisker change

*2.9 Social interaction test*

The social interaction test was conducted following a protocol by File and Hyde (17). Mice habituated to the experimental settings on two consecutive days. Therefore, mice were placed into single cages (Makrolon Type III) in the behavioral room for ten minutes each. The light intensity was set to 12 lux. Animals were placed in the test arena with a weight-matched (+/- 5 gram) partner for ten minutes. Only mice from the same experimental group were used as test partners. Test partners had no direct social contact before the test since mice were singly housed. For each experimental unit (n=2), a combined score was calculated. The time mice spent in active social interaction (parameters: sniffing, grooming, playing, following, and walking on each other) and in passive social interaction (sitting or walking next to each other but not interacting) was assessed manually.

*2.10 Black-white box*

The black-white box test is conducted with an apparatus consisting of a white compartment (40 x 40 cm), connected to a black compartment (40 x 20 cm) by a 10 x 10 cm passage area. The black compartment was covered with a top lid. The light intensity was set at 50 lux for the white compartment. Each trial lasted five minutes and was monitored by video recording (CCTV Camera, Panasonic, Suzhou, China). For test conduction, animals were placed individually in the white box with its head facing the black compartment. A person blinded for group allocation scored the following parameters: latency to enter the black box, time spent in the black and white box, and stretching postures.

*2.11 Elevated-plus maze*

The elevated-plus maze is conducted with an apparatus consisting of two open arm areas and two closed arm areas with sidewalls of 12.5 cm height each. A central platform is arranged in the form of a plus sign. Light intensity was set at 40 lux for the open arms and 20 lux for the closed arms. A central platform is arranged in the form of a plus sign. Animals were placed individually on the central platform with the head facing the open arm, which was the same for all animals. Each trial lasted five minutes, and the following parameters were analyzed based on the tracking of the mice: (EthoVision, version 8.5, Noldus, Wageningen, The Nederlands): total distance moved, velocity, and time spent in closed and open arms. In addition, the number of head dips (looking down from the open arms) was scored manually by a person unaware of group allocation.

*2.12 Analysis of fecal corticosterone metabolites (FCM)*

Baseline values were examined for the analyses in the three adult epilepsy models. Therefore, fecal samples were collected the week before the surgical intervention. Further samples were collected at project-specific time points throughout the study. For the analyses in the present study, only the data from the last timepoint, shortly before the perfusion of the mice in the chronic phase (intrahippocampal kainate model) and the phase of generalized seizures (kindling models), were considered. Following a protocol developed by the research group FOR 2591, animals were placed in a fresh cage in the morning (7:00 to 9:00 a.m.), supplemented with bedding material (Lignocel Select), and all feces were collected.

For the investigations in the two genetic models and in C57BL/6J wild-type mice, samples were collected from open field arenas in the morning (7:00 a.m. to 1:00 p.m.), following the open field test. In addition, samples were collected from Makrolon type II open cages (Ehret GmbH & Co. KG), supplemented with bedding material (Lignocel Select), where the animals had been placed individually after the open field test.

Feces were stored frozen at - 20 °C, dried, and homogenized. Afterward, a 0.05 g portion of each sample was extracted with 1 ml 80% methanol. The analysis of fecal corticosterone metabolites was carried out with a 5α-pregnane-3β,11β,21-triol-20-one enzyme immunoassay, which has been established and fully validated for the measurement of fecal corticosterone metabolites in mice (18, 19).

1. ***Clinical evaluation protocol***

| **Breeder:** |  | **Strain:** |  | **Weight:** | **Responsible person:** |  |
| --- | --- | --- | --- | --- | --- | --- |
| **Arrival / Age:** |  | **Animal-ID.** |  | **Genotype:** | **Signature:** |  |
| **Species:** |  | **Sex:** |  |  |  |  |

| Parameter | Description of scoring | Score | Date: | | | Date: | | | Date: | | | Date: | | | Date: | | | Date: | | |
| --- | --- | --- | --- | --- | --- | --- | --- | --- | --- | --- | --- | --- | --- | --- | --- | --- | --- | --- | --- | --- |
|  |  |  |  | Mark abnormality with X | |  | Mark abnormality with X | |  | Mark abnormality with X | |  | Mark abnormality with X | |  | Mark abnormality with X | |  | Mark abnormality with X | |
|  |  |  | Score | Eye/  Posture/  Body aperture | Fur/ Hair coat | Score | Eye/  Posture/  Body aperture | Fur/ Hair coat | Score | Eye/  Posture/  Body aperture | Fur/ Hair coat | Score | Eye/  Posture/  Body aperture | Fur/ Hair coat | Score | Eye/  Posture/  Body aperture | Fur/ Hair coat | Score | Eye/  Posture/  Body aperture | Fur/ Hair coat |
| Appearance | Smooth, shiny coat, coat care, eye shiny | 0 |  |  |  |  |  |  |  |  |  |  |  |  |  |  |  |  |  |  |
|  | Dull coat, grooming limited or absent, eyes dull | 1 |  |  |  |  |  |  |  |  |  |  |  |  |  |  |  |  |  |  |
|  | Partially shaggym slightly ruffled coat, eye discharge, nasal discharge, eyes sunken, stuck orifices | 2 |  |  |  |  |  |  |  |  |  |  |  |  |  |  |  |  |  |  |
|  | Completely shaggy, dull coat, abnormal body posture, bent back, eyes closed | 3 |  |  |  |  |  |  |  |  |  |  |  |  |  |  |  |  |  |  |
| Body weight | In adult animals constant, in growing animals with regard to growth curve constant | 0 |  | | |  | | |  | | |  | | |  | | |  | | |
|  | 5-9 % Weight loss | 1 |  |  |  |  |  |  |  |  |  |  |  |  |  |  |  |  |  |  |
|  | 10-20% Weight loss | 2 |  |  |  |  |  |  |  |  |  |  |  |  |  |  |  |  |  |  |
|  | > 20% Weight loss | 3 |  |  |  |  |  |  |  |  |  |  |  |  |  |  |  |  |  |  |
| Seizure activity | Constant  Pharmacoresistant seizure activity without interruption (no response to benzodiazepines) | 3 |  | | |  | | |  | | |  | | |  | | |  | | |
| Behavior | Attentive, active, social contacts, curious | 0 |  | | |  | | |  | | |  | | |  | | |  | | |
|  | Decreased reactions, restricted or excessize activity | 1 |  |  |  |  |  |  |  |  |  |  |  |  |  |  |  |  |  |  |
|  | Crouching posture, partial separation from the group | 2 |  |  |  |  |  |  |  |  |  |  |  |  |  |  |  |  |  |  |
|  | Apathetic, no reaction or aggressiveness when handled, isolation, stereotypes | 3 |  |  |  |  |  |  |  |  |  |  |  |  |  |  |  |  |  |  |
| Motor functions | Movement normal | 0 |  | | |  | | |  | | |  | | |  | | |  | | |
|  | Movement reduced or excessive,  presence of abnormal moving patterns | 1 |  |  |  |  |  |  |  |  |  |  |  |  |  |  |  |  |  |  |
|  | Severly restricted movement, presence of paralysis in individual extremities | 2 |  |  |  |  |  |  |  |  |  |  |  |  |  |  |  |  |  |  |
|  | Complete immobility | 3 |  |  |  |  |  |  |  |  |  |  |  |  |  |  |  |  |  |  |
| Other | Rectum prolapse | 3 |  | | |  | | |  | | |  | | |  | | |  | | |
|  | Vocalization (continuous) | 3 |  | | |  | | |  | | |  | | |  | | |  | | |
|  | Tumors | 3 |  | | |  | | |  | | |  | | |  | | |  | | |
|  | Automutilation | 3 |  | | |  | | |  | | |  | | |  | | |  | | |

| Wound infection | Inconspicuous wound area, no infection detectable | 0 |  |  |  |  |  |  |
| --- | --- | --- | --- | --- | --- | --- | --- | --- |
|  | Moderate inflammation in the wound area, defence behavior (if applicable) | 2 |  |  |  |  |  |  |
|  | High inflammation in the wound area, defence behavior (if applicable) | 3 |  |  |  |  |  |  |
| DAILY SUM SCORE | |  |  |  |  |  |  |  |
| Cumulative sum score | |  |  |  |  |  |  |  |
| Assessor (signature) | |  |  |  |  |  |  |  |

**Frequency of inspection:**

Animals are inspected on a daily basis. The body condition score is taken into account. In case of changes in the body condition score, the body weight is recorded.

Entries are made in the individual score sheet if there are any deviations.

If there are signs of dehydration (degree of dehydration is checked by skinfold measurement), fluid substitution (10 ml / kg s.c.) is given.

If feed intake is reduced, animals are fed baby food.

**Termination criteria:**

If a single parameter reaches a score of 3, this corresponds to the humane endpoint; the animal is killed immediately in accordance with animal welfare.

General Condition: Dirty coat, stuck/wet body openings, abnormal posture, arched back, diarrhea, cloudy and sunken eyes.

Body weight: Rapid weight loss (15% to 20% within a few days); prolonged weight loss.

Spontaneous behavior: Apathy, behavioral stereotypies, hyperlocomotion, incoordination, persistent vocalization, automutilation.

Clinical findings: Lung sounds, nasal discharge, marked icterus, anemia, cyanosis, paralysis, convulsions, markedly discolored urine, polyuria or anuria, bleeding from a body orifice, rectal prolapse, tumors, high-grade inflammation in the wound area possibly defensive behavior.

To calculate the sum score, the score values of the individual parameters are added up.

Sum score 2-3: low severity, control frequency is increased; possibly consultation with the responsible veterinarian regarding measures to be taken

(e.g. adaptation of the diet or housing conditions, euthanasia); if this sum score persists for another day, sum score 4 is reached.

Sum score 4: increased (moderate) severity, immediate consultation with the responsible veterinarian regarding measures to be taken

(e.g. isolation, adaptation of the diet or housing conditions, possibly euthanasia);

if this sum score remains for another day, the animal is killed in accordance with animal welfare requirements.

Sum score ≥ 5: termination criterion for the experiment is reached, the animal is killed immediately in a manner appropriate to animal welfare.

Individual score 1: low stress; control frequency is increased; if score 1 persists, upgrade to score 2.

Individual score 2: increased (moderate) stress, immediate consultation with the responsible veterinarian regarding measures to be taken

(e.g. isolation, adaptation of diet or housing conditions, possibly euthanasia); if this individual score persists for another day, sum score 4 is reached.

**Animals with spontaneous seizures:**

Note that in phases with spontaneous seizures (e.g., after status epilepticus) or in genetic spontaneous seizures, the condition of the coat/fur is not considered in the scoring because animals with repeated spontaneous seizures characteristically neglect grooming. Similarly, as stated in the applications, in phases with spontaneous seizures, behavioral changes such as altered reactions especially increased aggression, hypo- or hyperactivity are recorded but not included in the scoring, as this may characteristically occur due to seizures (e.g., in postictal phases).

**Days after status epilepticus:**

In the first days after induction of status epilepticus, deviations will be accepted as specified in the animal study permit applications. Termination will occur - as indicated in the applications - in the first 7 days after status epilepticus if inactivity, significant incoordination, acute respiratory distress, or excessive loss of body weight (>20%) persist for more than three days.

***Supplementary References***

1. Buchecker V, Koska I, Pace C, Talbot SR, Palme R, Bleich A, et al. Toward evidence-based severity assessment in mouse models with repeated seizures: (II.) Impact of surgery and intrahippocampal kainate. Eur Surg Res. 2022.

2. Boldt L, Koska I, Maarten van Dijk R, Talbot SR, Miljanovic N, Palme R, et al. Toward evidence-based severity assessment in mouse models with repeated seizures: I. Electrical kindling. Epilepsy Behav. 2021;115:107689.

3. Reiber M, Koska I, Pace C, Schönhoff K, von Schumann L, Palme R, et al. Development of behavioral patterns in young C57BL/6J mice: a home cage-based study. Scientific Reports. 2022;12(1):2550.

4. Reiber M, Miljanovic N, Schönhoff K, Palme R, Potschka H. Behavioral phenotyping of young *Scn1a* haploinsufficient mice. Epilepsy Behav. 2022;136:108903.

5. Kuo FS, Cleary CM, LoTurco JJ, Chen X, Mulkey DK. Disordered breathing in a mouse model of Dravet syndrome. Elife. 2019;8.

6. Ricobaraza A, Mora-Jimenez L, Puerta E, Sanchez-Carpintero R, Mingorance A, Artieda J, et al. Epilepsy and neuropsychiatric comorbidities in mice carrying a recurrent Dravet syndrome SCN1A missense mutation. Sci Rep. 2019;9(1):14172.

7. Tang SH, Silva FJ, Tsark WM, Mann JR. A Cre/loxP-deleter transgenic line in mouse strain 129S1/SvImJ. Genesis. 2002;32(3):199-202.

8. Miljanovic N, Hauck SM, van Dijk RM, Di Liberto V, Rezaei A, Potschka H. Proteomic signature of the Dravet syndrome in the genetic Scn1a-A1783V mouse model. Neurobiol Dis. 2021;157:105423.

9. Reiber M, Stirling H, Sprengel R, Gass P, Palme R, Potschka H. Phenotyping Young GluA1 Deficient Mice – A Behavioral Characterization in a Genetic Loss-of-Function Model. Frontiers in Behavioral Neuroscience. 2022;16.

10. Zamanillo D, Sprengel R, Hvalby O, Jensen V, Burnashev N, Rozov A, et al. Importance of AMPA receptors for hippocampal synaptic plasticity but not for spatial learning. Science. 1999;284(5421):1805-11.

11. Jirkof P, Fleischmann T, Cesarovic N, Rettich A, Vogel J, Arras M. Assessment of postsurgical distress and pain in laboratory mice by nest complexity scoring. Lab Anim. 2013;47(3):153-61.

12. Klein S, Bankstahl JP, Löscher W, Bankstahl M. Sucrose consumption test reveals pharmacoresistant depression-associated behavior in two mouse models of temporal lobe epilepsy. Exp Neurol. 2015;263:263-71.

13. Deacon RM. Burrowing in rodents: a sensitive method for detecting behavioral dysfunction. Nat Protoc. 2006;1(1):118-21.

14. Baran SW, Bratcher N, Dennis J, Gaburro S, Karlsson EM, Maguire S, et al. Emerging Role of Translational Digital Biomarkers Within Home Cage Monitoring Technologies in Preclinical Drug Discovery and Development. Frontiers in Behavioral Neuroscience. 2022;15.

15. Irwin S. Comprehensive observational assessment: Ia. A systematic, quantitative procedure for assessing the behavioral and physiologic state of the mouse. Psychopharmacologia. 1968;13(3):222-57.

16. Langford DJ, Bailey AL, Chanda ML, Clarke SE, Drummond TE, Echols S, et al. Coding of facial expressions of pain in the laboratory mouse. Nat Methods. 2010;7(6):447-9.

17. File SE, Hyde JR. Can social interaction be used to measure anxiety? Br J Pharmacol. 1978;62(1):19-24.

18. Touma C, Sachser N, Möstl E, Palme R. Effects of sex and time of day on metabolism and excretion of corticosterone in urine and feces of mice. Gen Comp Endocrinol. 2003;130(3):267-78.

19. Touma C, Palme R, Sachser N. Analyzing corticosterone metabolites in fecal samples of mice: a noninvasive technique to monitor stress hormones. Horm Behav. 2004;45(1):10-22.
