# Supplementary material for: Evidence-based comparative severity assessment in young and adult mice
Source: PLoS One. 2023 Oct 20;18(10):e0285429. doi: 10.1371/journal.pone.0285429 (PMC10588901; doi:10.1371/journal.pone.0285429)
Supplement: S2 Fig — Animals from the intrahippocampal kainate model are highlighted as follows: individual data from animals with only one seizure are circled in grey. In comparison, data from animals that showed more than one seizure are circled in black. The raw data underlying this figure are available in the Figshare repository https://doi.org/10.6084/m9.figshare.22759148.v1. (PDF) [file pone.0285429.s003.pdf]

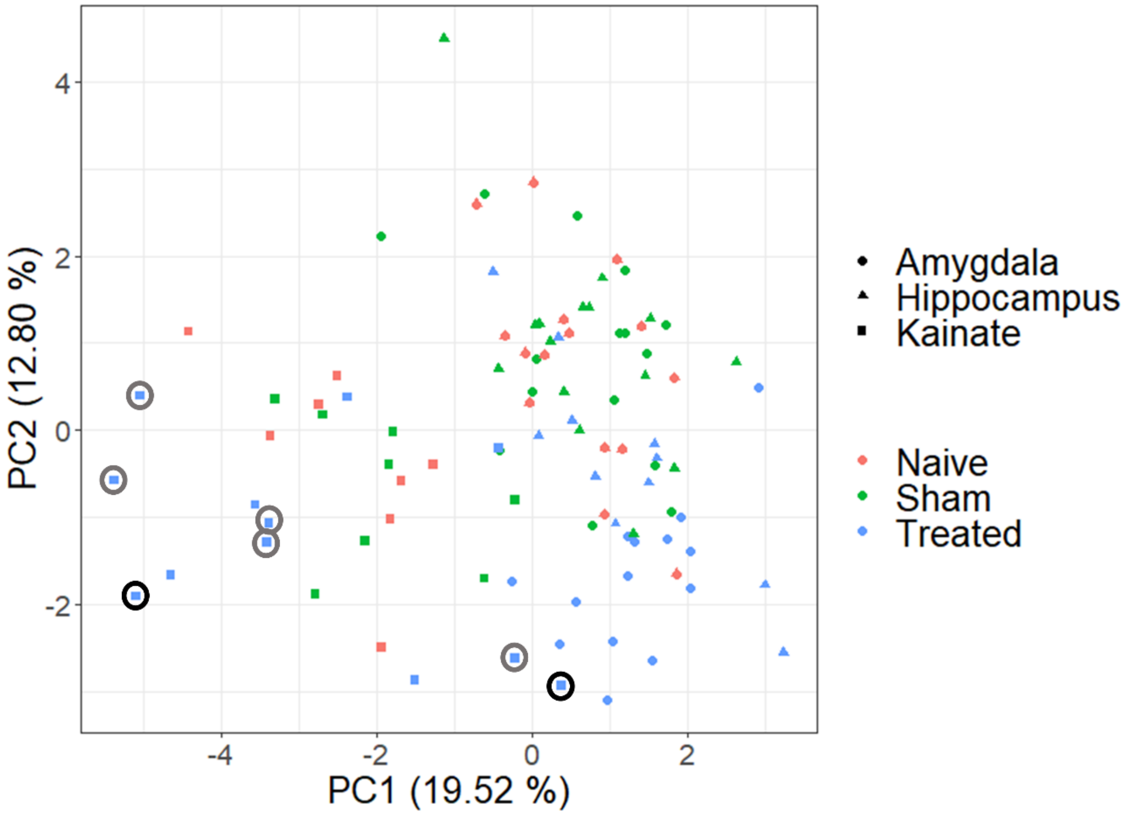

Fig S2. Results from one PCA run across the three adult epilepsy models. Animals from the intrahippocampal kainate model are highlighted as follows: individual data from animals with only one seizure are circled in grey. In comparison, data from animals that showed more than one seizure are circled in black.
