# Supplementary figures and images for: Evidence-based comparative severity assessment in young and adult mice
Source: PLoS One. 2023 Oct 20;18(10):e0285429. doi: 10.1371/journal.pone.0285429 (PMC10588901; doi:10.1371/journal.pone.0285429)

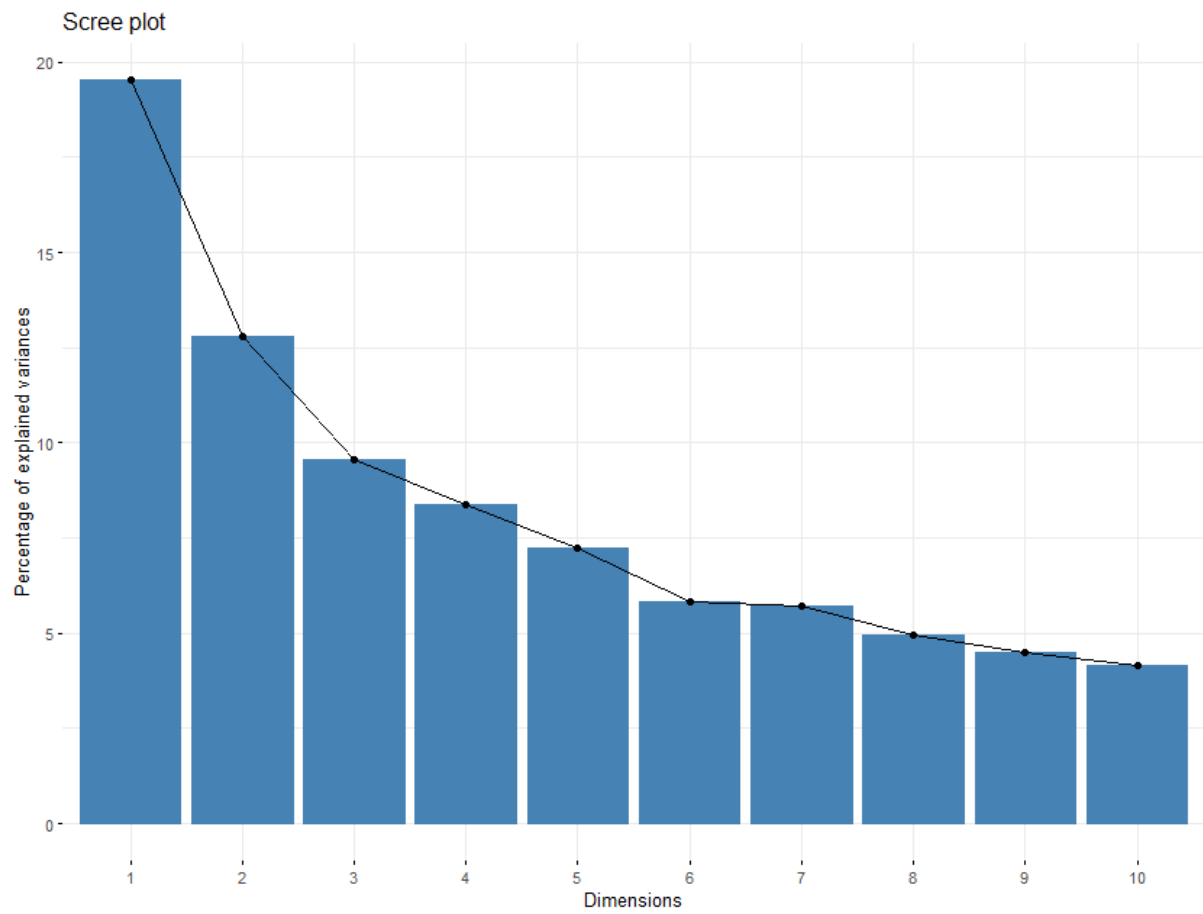

**Fig S3. Scree plot.** Three adult epilepsy models.

Supplement: S3 Fig — Three adult epilepsy models. The raw data underlying this figure are available in the Figshare repository https://doi.org/10.6084/m9.figshare.22759148.v1. (PDF) [file pone.0285429.s004.pdf]

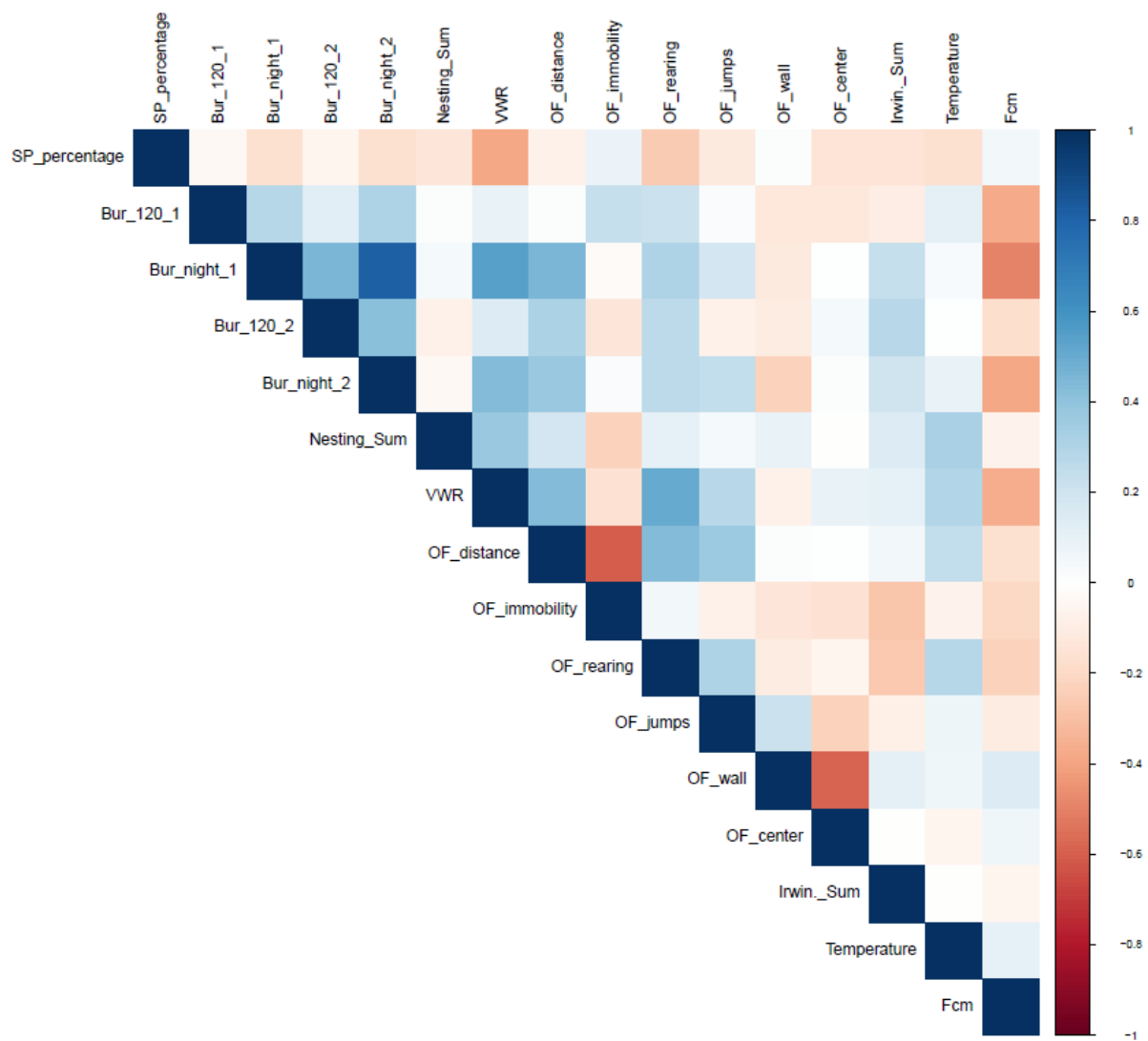

**Fig S4. Correlation analysis (Spearman).** C57BL/6J model: adolescence (P25, P36, P50).

Supplement: S4 Fig — C57BL/6J model: adolescence (P25, P36, P50). The raw data underlying this figure are available in the Figshare repository https://doi.org/10.6084/m9.figshare.22759148.v1. (PDF) [file pone.0285429.s005.pdf]

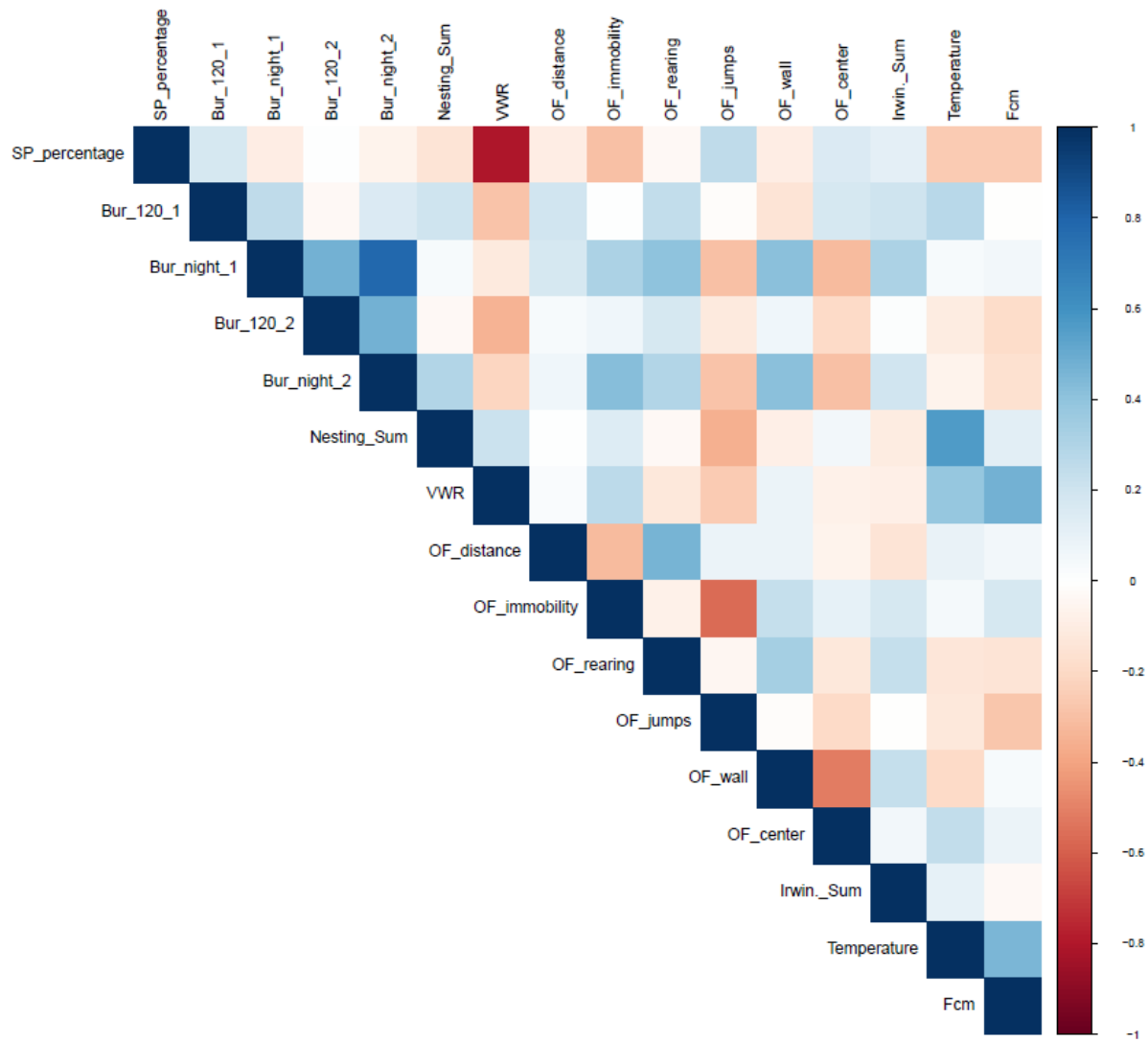

**Fig S5: Correlation analysis (Spearman).** C57BL/6J model: mature adult (P120).

Supplement: S5 Fig — C57BL/6J model: mature adult (P120). The raw data underlying this figure are available in the Figshare repository https://doi.org/10.6084/m9.figshare.22759148.v1. (PDF) [file pone.0285429.s006.pdf]

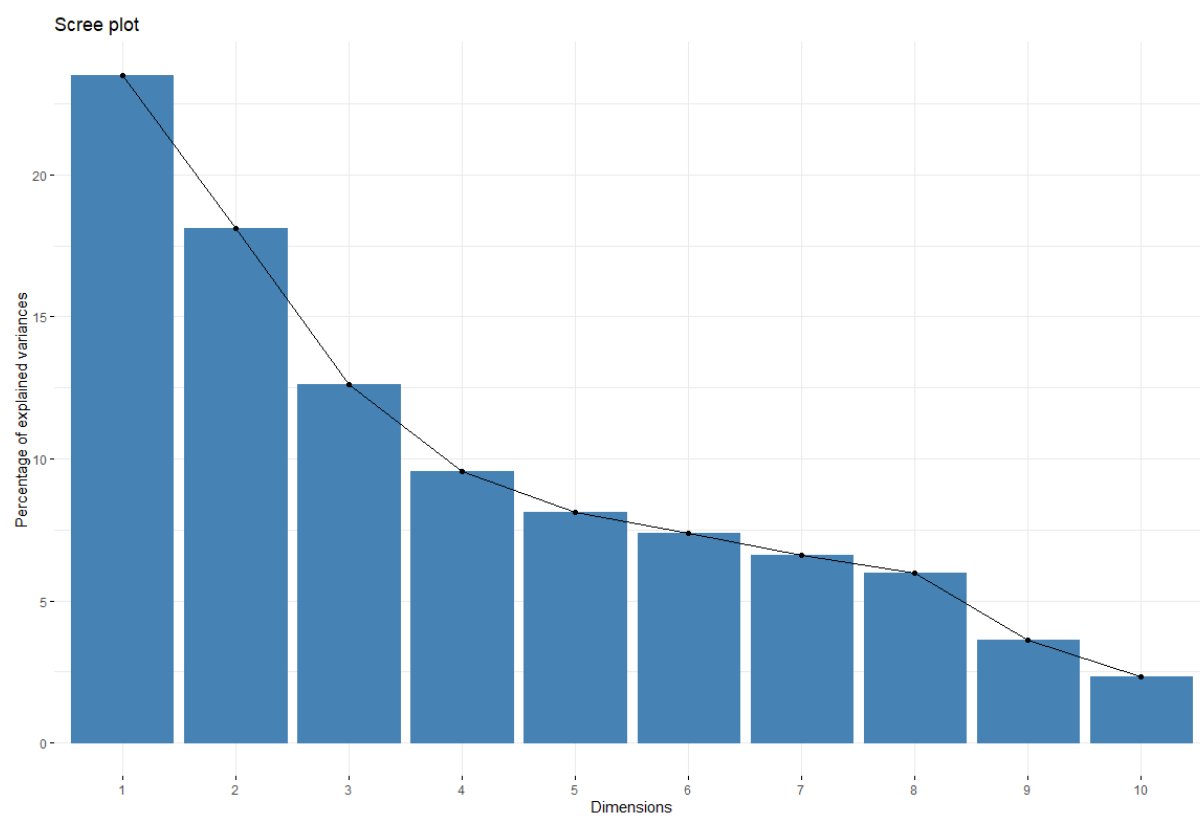

**Fig S8. Scree plot.** Genetic models: early adolescence.

Supplement: S8 Fig — Genetic models: early adolescence. The raw data underlying this figure are available in the Figshare repository https://doi.org/10.6084/m9.figshare.22759148.v1. (PDF) [file pone.0285429.s009.pdf]

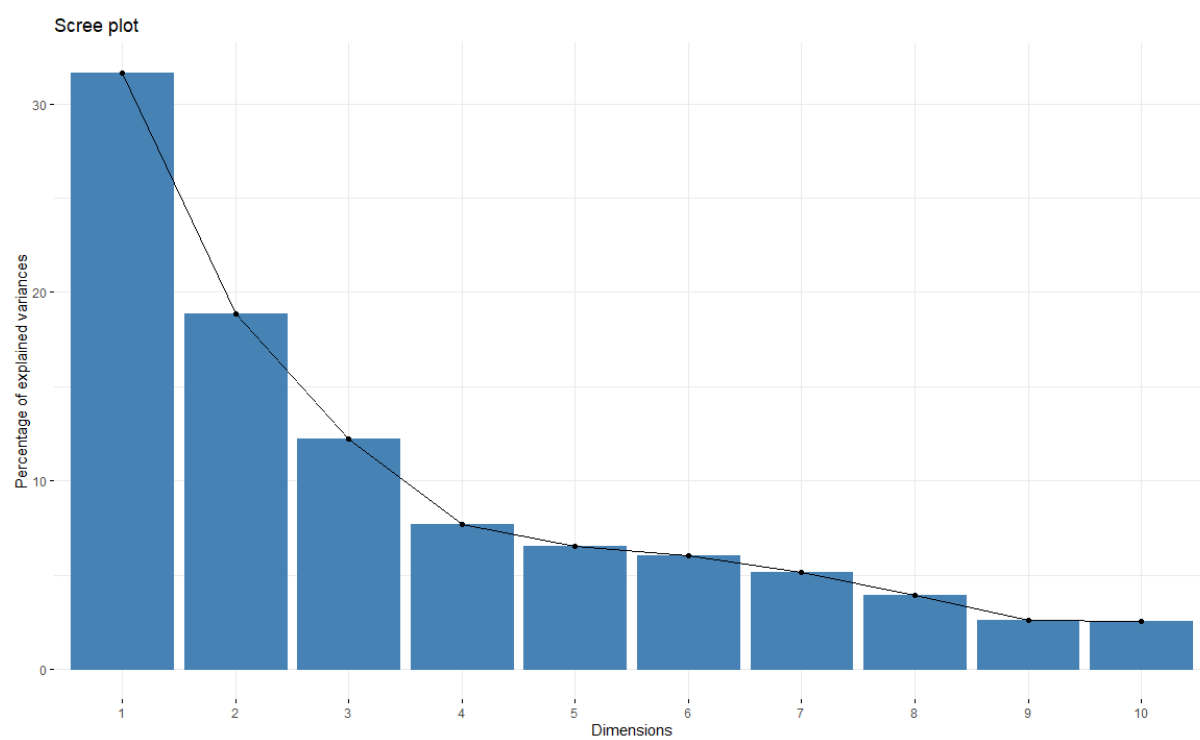

**Fig S9. Scree plot.** Genetic models: late adolescence.

Supplement: S9 Fig — Genetic models: late adolescence. The raw data underlying this figure are available in the Figshare repository https://doi.org/10.6084/m9.figshare.22759148.v1. (PDF) [file pone.0285429.s010.pdf]

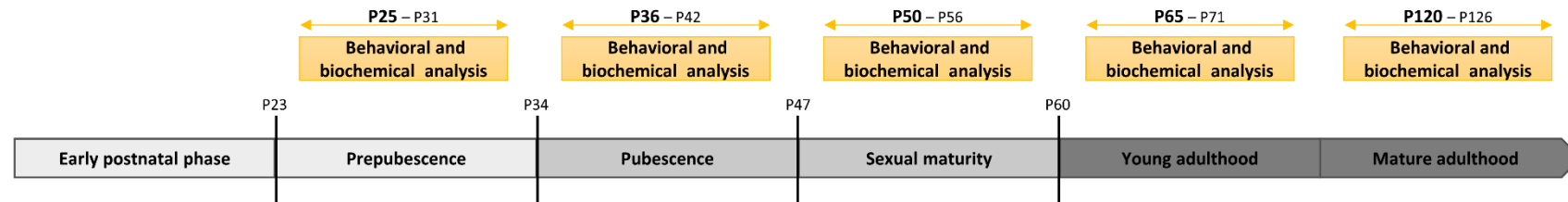

**Fig S10.** Overview of the experimental timeline of the C57BL/6J wild-type model.

Supplement: S10 Fig — (PDF) [file pone.0285429.s011.pdf]
