# Supplementary material for: Evidence-based comparative severity assessment in young and adult mice
Source: PLoS One. 2023 Oct 20;18(10):e0285429. doi: 10.1371/journal.pone.0285429 (PMC10588901; doi:10.1371/journal.pone.0285429)
Supplement: S6 Fig — The data set comprises the clinical evaluation (clinical score) and the set of behavioral/biochemical variables. The raw data underlying this figure are available in the Figshare repository https://doi.org/10.6084/m9.figshare.22759148.v1. (PDF) [file pone.0285429.s007.pdf]

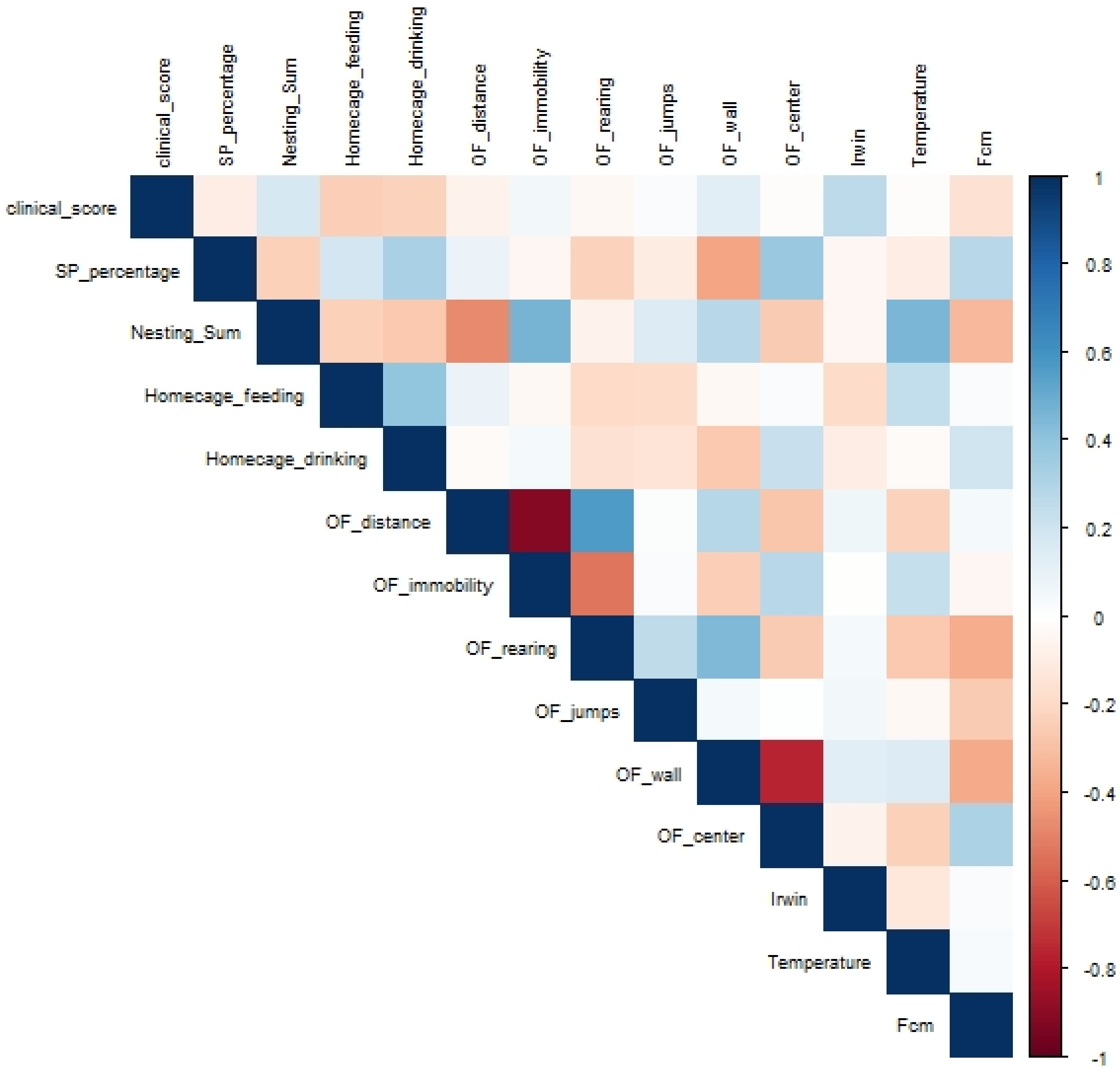

Fig S6. Correlation analysis (Spearman) for the genetic models, early adolescence. The data set comprises the clinical evaluation (clinical score) and the set of behavioral/biochemical variables.
