# Supplementary material for: Evidence-based comparative severity assessment in young and adult mice
Source: PLoS One. 2023 Oct 20;18(10):e0285429. doi: 10.1371/journal.pone.0285429 (PMC10588901; doi:10.1371/journal.pone.0285429)
Supplement: S7 Fig — The data set comprises the clinical evaluation (clinical score) and the set of behavioral/biochemical variables. In both genetic models, clinical scores were not elevated during late adolescence (all animals were given a clinical score of zero in the assessment). The raw data underlying this figure are available in the Figshare repository https://doi.org/10.6084/m9.figshare.22759148.v1. (PDF) [file pone.0285429.s008.pdf]

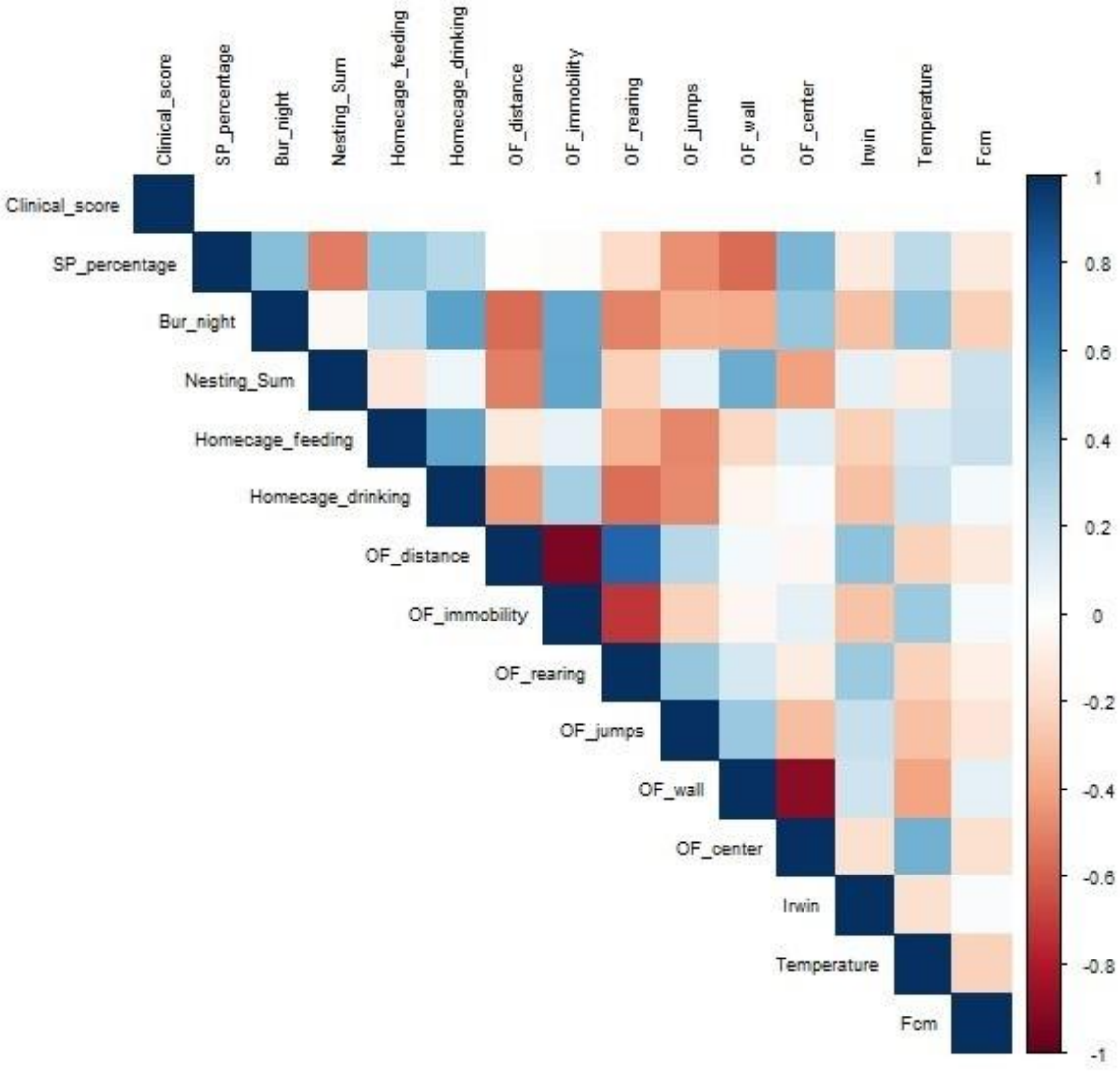

Fig S7. Correlation analysis (Spearman) for the genetic models, late adolescence. The data set comprises the clinical evaluation (clinical score) and the set of behavioral/biochemical variables. In both genetic models, clinical scores were not elevated during late adolescence (all animals were given a clinical score of zero in the assessment).
