# Supplementary material for: Evidence-based comparative severity assessment in young and adult mice
Source: PLoS One. 2023 Oct 20;18(10):e0285429. doi: 10.1371/journal.pone.0285429 (PMC10588901; doi:10.1371/journal.pone.0285429)
Supplement: S1 Table — (PDF) [file pone.0285429.s012.pdf]

Parameter	Test	Meaning
Clinical_score	Clinical evaluation	Clinical assessment following the Directive 2010/63/EU. The clinical condition of the mice was assessed in addition to the set of behavioral and biochemical parameters.
Nesting	Nest building	Nest building activity on the day of the 10th generalized seizure (kindling model); with spontaneous seizure activity (week 11 post SE. post SE-models)
Bur_120/Bur_night	Burrowing	Burrowing behavior in the phase of generalized seizures. The displaced gravel was calculated in Gramm
Social_Int_active<br>Social_Int_passiv	Social interaction<br>Social interaction	The time spent in social interaction was recorded in seconds.
SP_percentage	Saccharin Preference	Saccharin preference was tested on 2 days compared to water. Then, the total consumed amount was calculated, and the percentage of saccharin intake was shown in percentage.
OF_distance	Open Field	Open Field distance moved total in centimeters.
OF_rearing	Open Field	Amount of rearing in the Open Field. Rearing was defined as vertical activity with the animal rising more than 45%
OF_jumps	Open Field	Amount of jumps against the wall in the Open Field.
OF_immobility	Open Field	Time in seconds, the animal was immobile in the Open Field.
OF_center	Open Field	Time in seconds the animals spent in the center region of the Open Field
OF_wall	Open Field	Time in seconds the animals spent in the wall region of the Open Field
OF_velocity	Open Field	The velocity of movement of the mice measured in the Open Field in sec/m.
BWB_WB	Black and White Box	Time in seconds the animals spent in the white box. The time the animals spent in the black box was also measured, but is not shown here.
BWB_stretching	Black and White Box	The number of stretching postures of the animal in the Black and White Box. Stretching was defined as the animal looking out of the black into the white box with its body elongated.
BWB_LT	Black and White Box	Latency to the first entry of the black box from the white box in which the animals were placed.
EPM_streching	Elevated Plus Maze	Number of stretching postures of the animal in the Elevated Plus Maze. Stretching was defined as the animal looking out of one of the closed arms into an open arm.
EPM_headdip	Elevated Plus Maze	Number of the times the animal looked down from one of the open arms.
EPM_closedarms	Elevated Plus Maze	Time the animal spent in both closed arms of the Elevated Plus Maze.
EPM_openarms	Elevated Plus Maze	Time the animal spent in both open arms of the Elevated Plus Maze.
EPM_open1.3	Elevated Plus Maze	Time the animal spent in the outer 1/3 of the both open arms.
Fcm	fecal corticosterone metabolites	Enzymimmonoassay-based analyses of fecal corticosterone metabolites (ng / 0.05 g feces)
Irwin	Irwin test	Observer-dependent Irwin score
Temperature	NA	Rectal body temperature in °C
Homecage_feeding	PhenoTyper assessments	Duration in zone ‘feeding’
Homecage_drinking	PhenoTyper assessments	Duration in zone ‘drinking’
VWR	Voluntary wheel running	Total distance moved in the wheel in meter

Table S1. Overview of the parameters (abbreviations) used for the correlation analyses.
