# Supplementary material for: Evidence-based comparative severity assessment in young and adult mice
Source: PLoS One. 2023 Oct 20;18(10):e0285429. doi: 10.1371/journal.pone.0285429 (PMC10588901; doi:10.1371/journal.pone.0285429)
Supplement: S2 Table — a. p-values for correlation analysis (Spearman). Three adult epilepsy models. b. Correlation coefficients (r) for correlation analysis (Spearman). Three adult epilepsy models. (ZIP) [file pone.0285429.s013.zip › S2a_Table.pdf]

	Clinical _score	Nesting	Bur_120	Bur_ night	Fcm	Irwin	OF_ distance	OF_ center	OF_wall	OF_ immobility	OF_ rearing	OF_ velocity	BWB_ LT	BWB_ streching	BWB_ WB	EPM_ openarms	EPM_ closedarms	EPM_ open1.3	EPM_ headdip	EPM_ streching	Social_ Int_passive	Social_ Int_active	SP_ percentage
**Clinical_score**	NA	0.590	0.011	0.063	0.055	0.000	0.011	0.343	0.070	0.897	0.007	0.000	0.175	0.051	0.098	0.489	0.041	0.000	0.034	0.020	0.000	0.052	0.240
**Nesting**	0.590	NA	0.684	0.885	0.474	0.000	0.381	0.471	0.006	0.299	0.001	0.627	0.472	0.839	0.051	0.063	0.017	0.543	0.000	0.795	0.858	0.131	0.053
**Bur_120**	0.011	0.684	NA	0.000	0.218	0.007	0.646	0.152	0.073	0.570	0.308	0.028	0.688	0.472	0.899	0.297	0.619	0.892	0.405	0.440	0.003	0.859	0.546
**Bur_night**	0.063	0.885	0.000	NA	0.859	0.118	0.666	0.109	0.069	0.526	0.726	0.032	0.510	0.917	0.063	0.014	0.016	0.077	0.342	0.238	0.082	0.688	0.790
**Fcm**	0.055	0.474	0.218	0.859	NA	0.463	0.614	0.131	0.080	0.724	0.091	0.329	0.587	0.892	0.275	0.200	0.592	0.815	0.714	0.882	0.369	0.217	0.977
**Irwin**	0.000	0.000	0.007	0.118	0.463	NA	0.777	0.277	0.008	0.012	0.000	0.001	0.169	0.034	0.631	0.687	0.729	0.002	0.478	0.181	0.000	0.175	0.323
**OF_distance**	0.011	0.381	0.646	0.666	0.614	0.777	NA	0.273	0.925	0.000	0.000	0.000	0.910	0.633	0.101	0.977	0.059	0.234	0.002	0.012	0.231	0.079	0.703
**OF_center**	0.343	0.471	0.152	0.109	0.131	0.277	0.273	NA	0.000	0.421	0.792	0.092	0.146	0.240	0.068	0.718	0.232	0.048	0.416	0.039	0.023	0.340	0.670
**OF_wall**	0.070	0.006	0.073	0.069	0.080	0.008	0.925	0.000	NA	0.474	0.000	0.072	0.044	0.981	0.259	0.907	0.332	0.017	0.950	0.009	0.001	0.056	0.455
**OF_immobility**	0.897	0.299	0.570	0.526	0.724	0.012	0.000	0.421	0.474	NA	0.000	0.000	0.575	0.003	0.097	0.817	0.186	0.072	0.091	0.282	0.260	0.586	0.956
**OF_rearing**	0.007	0.001	0.308	0.726	0.091	0.000	0.000	0.792	0.000	0.000	NA	0.762	0.236	0.001	0.036	0.775	0.510	0.001	0.632	0.094	0.000	0.083	0.667
**OF_velocity**	0.000	0.627	0.028	0.032	0.329	0.001	0.000	0.092	0.072	0.000	0.762	NA	0.285	0.108	0.249	0.355	0.002	0.000	0.000	0.000	0.000	0.001	0.589
**BWB_LT**	0.175	0.472	0.688	0.510	0.587	0.169	0.910	0.146	0.044	0.575	0.236	0.285	NA	0.511	0.612	0.843	0.704	0.078	0.487	0.663	0.973	0.051	0.144
**BWB_streching**	0.051	0.839	0.472	0.917	0.892	0.034	0.633	0.240	0.981	0.003	0.001	0.108	0.511	NA	0.000	0.979	0.417	0.007	0.851	0.065	0.123	0.070	0.599
**BWB_WB**	0.098	0.051	0.899	0.063	0.275	0.631	0.101	0.068	0.259	0.097	0.036	0.249	0.612	0.000	NA	0.001	0.000	0.229	0.001	0.440	0.563	0.176	0.526
**EPM_openarms**	0.489	0.063	0.297	0.014	0.200	0.687	0.977	0.718	0.907	0.817	0.775	0.355	0.843	0.979	0.001	NA	0.000	0.000	0.000	0.000	0.260	0.716	0.076
**EPM_closedarms**	0.041	0.017	0.619	0.016	0.592	0.729	0.059	0.232	0.332	0.186	0.510	0.002	0.704	0.417	0.000	0.000	NA	0.000	0.000	0.000	0.012	0.732	0.034
**EPM_open1.3**	0.000	0.543	0.892	0.077	0.815	0.002	0.234	0.048	0.017	0.072	0.001	0.000	0.078	0.007	0.229	0.000	0.000	NA	0.000	0.002	0.000	0.001	0.039
**EPM_headdip**	0.034	0.000	0.405	0.342	0.714	0.478	0.002	0.416	0.950	0.091	0.632	0.000	0.487	0.851	0.001	0.000	0.000	0.000	NA	0.000	0.000	0.060	0.018
**EPM_streching**	0.020	0.795	0.440	0.238	0.882	0.181	0.012	0.039	0.009	0.282	0.094	0.000	0.663	0.065	0.440	0.000	0.000	0.002	0.000	NA	0.000	0.441	0.985
**Social_Int_passive**	0.000	0.858	0.003	0.082	0.369	0.000	0.231	0.023	0.001	0.260	0.000	0.000	0.973	0.123	0.563	0.260	0.012	0.000	0.000	0.000	NA	0.058	0.681
**Social_Int_active**	0.052	0.131	0.859	0.688	0.217	0.175	0.079	0.340	0.056	0.586	0.083	0.001	0.051	0.070	0.176	0.716	0.732	0.001	0.060	0.441	0.058	NA	0.488
**SP_percentage**	0.240	0.053	0.546	0.790	0.977	0.323	0.703	0.670	0.455	0.956	0.667	0.589	0.144	0.599	0.526	0.076	0.034	0.039	0.018	0.985	0.681	0.488	NA

Table S2a. p-values for correlation analysis (Spearman). Three adult epilepsy models.
