# Supplementary material for: Evidence-based comparative severity assessment in young and adult mice
Source: PLoS One. 2023 Oct 20;18(10):e0285429. doi: 10.1371/journal.pone.0285429 (PMC10588901; doi:10.1371/journal.pone.0285429)
Supplement: S2 Table — a. p-values for correlation analysis (Spearman). Three adult epilepsy models. b. Correlation coefficients (r) for correlation analysis (Spearman). Three adult epilepsy models. (ZIP) [file pone.0285429.s013.zip › S2b_Table.pdf]

| | Clinical _score | Nesting | Bur_120 | Bur_ night | Fcm | Irwin | OF_ distance | OF_ center | OF_wall | OF_ immobility | OF_ rearing | OF_ velocity | BWB_ LT | BWB_ streching | BWB_ WB | EPM_ openarms | EPM_ closedarms | EPM_ open1.3 | EPM_ headdip | EPM_ streching | Social_ Int_passive | Social_ Int_active | SP_ percentage |
|---|---|---|---|---|---|---|---|---|---|---|---|---|---|---|---|---|---|---|---|---|---|---|---|
| **Clinical_score** | 1.000 | -0.050 | -0.250 | -0.180 | -0.180 | 0.550 | -0.240 | -0.090 | 0.170 | -0.010 | 0.250 | -0.540 | -0.130 | -0.180 | -0.160 | -0.070 | 0.190 | -0.350 | -0.200 | 0.220 | 0.510 | -0.180 | 0.110 |
| **Nesting** | -0.050 | 1.000 | 0.040 | -0.010 | 0.070 | -0.340 | -0.080 | 0.070 | -0.260 | 0.100 | -0.310 | -0.050 | -0.070 | -0.020 | -0.180 | -0.180 | 0.230 | 0.060 | -0.330 | 0.020 | -0.020 | 0.140 | 0.180 |
| **Bur_120** | -0.250 | 0.040 | 1.000 | 0.680 | 0.120 | -0.260 | 0.050 | 0.140 | -0.170 | 0.060 | -0.100 | 0.210 | -0.040 | 0.070 | 0.010 | 0.100 | -0.050 | 0.010 | 0.080 | -0.080 | -0.290 | -0.020 | 0.060 |
| **Bur_night** | -0.180 | -0.010 | 0.680 | 1.000 | -0.020 | -0.150 | 0.040 | 0.160 | -0.180 | 0.060 | -0.030 | 0.210 | -0.060 | -0.010 | 0.180 | 0.240 | -0.230 | 0.170 | 0.090 | -0.120 | -0.170 | -0.040 | -0.030 |
| **Fcm** | -0.180 | 0.070 | 0.120 | -0.020 | 1.000 | -0.070 | 0.050 | 0.140 | -0.170 | 0.030 | -0.160 | 0.090 | -0.050 | 0.010 | -0.100 | -0.120 | 0.050 | -0.020 | -0.040 | -0.010 | -0.090 | 0.120 | 0.000 |
| **Irwin** | 0.550 | -0.340 | -0.260 | -0.150 | -0.070 | 1.000 | 0.030 | -0.100 | 0.250 | -0.240 | 0.410 | -0.300 | -0.130 | -0.200 | -0.050 | 0.040 | -0.030 | -0.290 | 0.070 | 0.130 | 0.400 | -0.130 | -0.090 |
| **OF_distance** | -0.240 | -0.080 | 0.050 | 0.040 | 0.050 | 0.030 | 1.000 | 0.100 | -0.010 | -0.800 | 0.420 | 0.790 | 0.010 | -0.050 | 0.160 | 0.000 | -0.180 | 0.110 | 0.290 | -0.240 | -0.110 | 0.170 | -0.040 |
| **OF_center** | -0.090 | 0.070 | 0.140 | 0.160 | 0.140 | -0.100 | 0.100 | 1.000 | -0.760 | -0.080 | -0.030 | 0.160 | -0.140 | -0.110 | 0.170 | 0.030 | -0.110 | 0.190 | 0.080 | -0.190 | -0.210 | 0.090 | 0.040 |
| **OF_wall** | 0.170 | -0.260 | -0.170 | -0.180 | -0.170 | 0.250 | -0.010 | -0.760 | 1.000 | -0.070 | 0.340 | -0.170 | 0.190 | 0.000 | -0.110 | -0.010 | 0.090 | -0.220 | -0.010 | 0.250 | 0.300 | -0.180 | -0.070 |
| **OF_immobility** | -0.010 | 0.100 | 0.060 | 0.060 | 0.030 | -0.240 | -0.800 | -0.080 | -0.070 | 1.000 | -0.580 | -0.460 | 0.050 | 0.270 | -0.160 | 0.020 | 0.130 | 0.170 | -0.160 | 0.100 | -0.110 | 0.050 | -0.010 |
| **OF_rearing** | 0.250 | -0.310 | -0.100 | -0.030 | -0.160 | 0.410 | 0.420 | -0.030 | 0.340 | -0.580 | 1.000 | 0.030 | -0.110 | -0.300 | 0.200 | 0.030 | -0.060 | -0.310 | 0.050 | 0.160 | 0.350 | -0.160 | -0.040 |
| **OF_velocity** | -0.540 | -0.050 | 0.210 | 0.210 | 0.090 | -0.300 | 0.790 | 0.160 | -0.170 | -0.460 | 0.030 | 1.000 | 0.100 | 0.150 | 0.110 | 0.090 | -0.290 | 0.430 | 0.450 | -0.490 | -0.500 | 0.300 | -0.050 |
| **BWB_LT** | -0.130 | -0.070 | -0.040 | -0.060 | -0.050 | -0.130 | 0.010 | -0.140 | 0.190 | 0.050 | -0.110 | 0.100 | 1.000 | 0.060 | 0.050 | -0.020 | -0.040 | 0.170 | 0.070 | -0.040 | 0.000 | -0.180 | -0.140 |
| **BWB_streching** | -0.180 | -0.020 | 0.070 | -0.010 | 0.010 | -0.200 | -0.050 | -0.110 | 0.000 | 0.270 | -0.300 | 0.150 | 0.060 | 1.000 | -0.390 | 0.000 | 0.080 | 0.250 | -0.020 | -0.170 | -0.150 | 0.170 | 0.050 |
| **BWB_WB** | -0.160 | -0.180 | 0.010 | 0.180 | -0.100 | -0.050 | 0.160 | 0.170 | -0.110 | -0.160 | 0.200 | 0.110 | 0.050 | -0.390 | 1.000 | 0.310 | -0.400 | 0.110 | 0.300 | -0.070 | -0.060 | -0.130 | -0.060 |
| **EPM_openarms** | -0.070 | -0.180 | 0.100 | 0.240 | -0.120 | 0.040 | 0.000 | 0.030 | -0.010 | 0.020 | 0.030 | 0.090 | -0.020 | 0.000 | 0.310 | 1.000 | -0.790 | 0.370 | 0.560 | -0.330 | -0.110 | 0.030 | -0.170 |
| **EPM_closedarms** | 0.190 | 0.230 | -0.050 | -0.230 | 0.050 | -0.030 | -0.180 | -0.110 | 0.090 | 0.130 | -0.060 | -0.290 | -0.040 | 0.080 | -0.400 | -0.790 | 1.000 | -0.390 | -0.740 | 0.500 | 0.240 | -0.030 | 0.200 |
| **EPM_open1.3** | -0.350 | 0.060 | 0.010 | 0.170 | -0.020 | -0.290 | 0.110 | 0.190 | -0.220 | 0.170 | -0.310 | 0.430 | 0.170 | 0.250 | 0.110 | 0.370 | -0.390 | 1.000 | 0.340 | -0.280 | -0.330 | 0.320 | -0.200 |
| **EPM_headdip** | -0.200 | -0.330 | 0.080 | 0.090 | -0.040 | 0.070 | 0.290 | 0.080 | -0.010 | -0.160 | 0.050 | 0.450 | 0.070 | -0.020 | 0.300 | 0.560 | -0.740 | 0.340 | 1.000 | -0.500 | -0.380 | 0.180 | -0.220 |
| **EPM_streching** | 0.220 | 0.020 | -0.080 | -0.120 | -0.010 | 0.130 | -0.240 | -0.190 | 0.250 | 0.100 | 0.160 | -0.490 | -0.040 | -0.170 | -0.070 | -0.330 | 0.500 | -0.280 | -0.500 | 1.000 | 0.350 | -0.070 | 0.000 |
| **Social_Int_passive** | 0.510 | -0.020 | -0.290 | -0.170 | -0.090 | 0.400 | -0.110 | -0.210 | 0.300 | -0.110 | 0.350 | -0.500 | 0.000 | -0.150 | -0.060 | -0.110 | 0.240 | -0.330 | -0.380 | 0.350 | 1.000 | -0.180 | 0.040 |
| **Social_Int_active** | -0.180 | 0.140 | -0.020 | -0.040 | 0.120 | -0.130 | 0.170 | 0.090 | -0.180 | 0.050 | -0.160 | 0.300 | -0.180 | 0.170 | -0.130 | 0.030 | -0.030 | 0.320 | 0.180 | -0.070 | -0.180 | 1.000 | -0.070 |
| **SP_percentage** | 0.110 | 0.180 | 0.060 | -0.030 | 0.000 | -0.090 | -0.040 | 0.040 | -0.070 | -0.010 | -0.040 | -0.050 | -0.140 | 0.050 | -0.060 | -0.170 | 0.200 | -0.200 | -0.220 | 0.000 | 0.040 | -0.070 | 1.000 |

**Table S2b. Correlation coefficients (r) for correlation analysis (Spearman).** Three adult epilepsy models.
