# Supplementary material for: Evidence-based comparative severity assessment in young and adult mice
Source: PLoS One. 2023 Oct 20;18(10):e0285429. doi: 10.1371/journal.pone.0285429 (PMC10588901; doi:10.1371/journal.pone.0285429)
Supplement: S3 Table — (PDF) [file pone.0285429.s014.pdf]

Position	Parameter	Frequency
**1**	Bur_120	38
	BWB_LT	2
	BWB_streching	50
	BWB_WB	2
	Fcm	2
	Nesting	1
	OF_center	1
	OF_distance	1
	Social_Int_active	2
	SP_percentage	1
**2**	Bur_120	17
	BWB_LT	2
	BWB_streching	28
	BWB_WB	13
	Fcm	4
	Nesting	11
	OF_center	1
	OF_distance	2
	Social_Int_active	14
	SP_percentage	8
**3**	Bur_120	12
	BWB_LT	1
	BWB_streching	15
	BWB_WB	26
	Fcm	7
	Nesting	11
	OF_center	3
	OF_distance	4
	Social_Int_active	16
	SP_percentage	5

Table S3. Top 30 parameters after 100 PCA runs of the training set in the three adult epilepsy models.
