# Supplementary material for: Evidence-based comparative severity assessment in young and adult mice
Source: PLoS One. 2023 Oct 20;18(10):e0285429. doi: 10.1371/journal.pone.0285429 (PMC10588901; doi:10.1371/journal.pone.0285429)
Supplement: S4 Table — (PDF) [file pone.0285429.s015.pdf]

Group	Cluster	Amygdala n	Amygdala %	Hippocampus n	Hippocampus %	Kainate n	Kainate %
**Naïve**	6	37	2.57	37	2.57	25	3.90
	5	118	8.21	118	8.21	283	44.15
	4	201	13.98	201	13.98	165	25.74
	3	371	25.80	371	25.80	80	12.48
	2	511	35.54	511	35.54	57	8.89
	1	200	13.91	200	13.91	31	4.84
**Sham**	6	21	1.79	26	2.15	20	3.10
	5	44	3.74	59	4.88	202	31.32
	4	188	15.99	186	15.38	211	32.71
	3	311	26.45	305	25.23	120	18.60
	2	316	26.87	388	32.09	69	10.70
	1	296	25.17	245	20.26	23	3.57
**Treated**	6	49	4.43	137	15.64	361	37.53
	5	211	19.08	290	33.11	189	19.65
	4	469	42.41	221	25.23	174	18.09
	3	238	21.52	124	14.16	122	12.68
	2	101	9.13	62	7.08	69	7.17
	1	38	3.44	42	4.79	47	4.89

Table S4. Results from the *k*-means cluster allocation in the three adult epilepsy models.
