# Supplementary material for: Evidence-based comparative severity assessment in young and adult mice
Source: PLoS One. 2023 Oct 20;18(10):e0285429. doi: 10.1371/journal.pone.0285429 (PMC10588901; doi:10.1371/journal.pone.0285429)
Supplement: S5 Table — a. p-values for correlation analysis (Spearman). C57BL/6J model: adolescence (P25, P36, P50). b. Correlation coefficients (r) for correlation analysis (Spearman). C57BL/6J model: adolescence (P25, P36, P50). (ZIP) [file pone.0285429.s016.zip › S5a_Table.pdf]

| | SP_percentage | Bur_120_1 | Bur_night_1 | Bur_120_2 | Bur_night_2 | Nesting_Sum | VWR | OF_distance | OF_immobility | OF_rearing | OF_jumps | OF_wall | OF_center | Irwin_Sum | Temperature | Fcm |
|---|---|---|---|---|---|---|---|---|---|---|---|---|---|---|---|---|
| **SP_percentage** | NA | 0.649 | 0.071 | 0.534 | 0.071 | 0.120 | 0.000 | 0.396 | 0.382 | 0.004 | 0.197 | 0.872 | 0.110 | 0.104 | 0.067 | 0.575 |
| **Bur_120_1** | 0.649 | NA | 0.002 | 0.150 | 0.001 | 0.886 | 0.335 | 0.932 | 0.013 | 0.019 | 0.794 | 0.154 | 0.154 | 0.272 | 0.228 | 0.000 |
| **Bur_night_1** | 0.071 | 0.002 | NA | 0.000 | 0.000 | 0.638 | 0.000 | 0.000 | 0.744 | 0.001 | 0.053 | 0.185 | 0.966 | 0.012 | 0.781 | 0.000 |
| **Bur_120_2** | 0.534 | 0.150 | 0.000 | NA | 0.000 | 0.377 | 0.096 | 0.001 | 0.122 | 0.005 | 0.398 | 0.250 | 0.662 | 0.003 | 0.971 | 0.048 |
| **Bur_night_2** | 0.071 | 0.001 | 0.000 | 0.000 | NA | 0.658 | 0.000 | 0.000 | 0.851 | 0.004 | 0.009 | 0.012 | 0.887 | 0.025 | 0.327 | 0.000 |
| **Nesting_Sum** | 0.120 | 0.886 | 0.638 | 0.377 | 0.658 | NA | 0.000 | 0.053 | 0.012 | 0.258 | 0.694 | 0.348 | 0.913 | 0.110 | 0.000 | 0.475 |
| **VWR** | 0.000 | 0.335 | 0.000 | 0.096 | 0.000 | 0.000 | NA | 0.000 | 0.084 | 0.000 | 0.003 | 0.378 | 0.334 | 0.278 | 0.001 | 0.000 |
| **OF_distance** | 0.396 | 0.932 | 0.000 | 0.001 | 0.000 | 0.053 | 0.000 | NA | 0.000 | 0.000 | 0.000 | 0.949 | 0.985 | 0.583 | 0.010 | 0.060 |
| **OF_immobility** | 0.382 | 0.013 | 0.744 | 0.122 | 0.851 | 0.012 | 0.084 | 0.000 | NA | 0.557 | 0.356 | 0.139 | 0.078 | 0.002 | 0.430 | 0.020 |
| **OF_rearing** | 0.004 | 0.019 | 0.001 | 0.005 | 0.004 | 0.258 | 0.000 | 0.000 | 0.557 | NA | 0.001 | 0.241 | 0.503 | 0.003 | 0.002 | 0.013 |
| **OF_jumps** | 0.197 | 0.794 | 0.053 | 0.398 | 0.009 | 0.694 | 0.003 | 0.000 | 0.356 | 0.001 | NA | 0.019 | 0.012 | 0.335 | 0.429 | 0.214 |
| **OF_wall** | 0.872 | 0.154 | 0.185 | 0.250 | 0.012 | 0.348 | 0.378 | 0.949 | 0.139 | 0.241 | 0.019 | NA | 0.000 | 0.222 | 0.490 | 0.134 |
| **OF_center** | 0.110 | 0.154 | 0.966 | 0.662 | 0.887 | 0.913 | 0.334 | 0.985 | 0.078 | 0.503 | 0.012 | 0.000 | NA | 0.938 | 0.481 | 0.536 |
| **Irwin._Sum** | 0.104 | 0.272 | 0.012 | 0.003 | 0.025 | 0.110 | 0.278 | 0.583 | 0.002 | 0.003 | 0.335 | 0.222 | 0.938 | NA | 0.942 | 0.521 |
| **Temperature** | 0.067 | 0.228 | 0.781 | 0.971 | 0.327 | 0.000 | 0.001 | 0.010 | 0.430 | 0.002 | 0.429 | 0.490 | 0.481 | 0.942 | NA | 0.281 |
| **Fcm** | 0.575 | 0.000 | 0.000 | 0.048 | 0.000 | 0.475 | 0.000 | 0.060 | 0.020 | 0.013 | 0.214 | 0.134 | 0.536 | 0.521 | 0.281 | NA |

**Table S5a. p-values for correlation analysis (Spearman).** C57BL/6J model: adolescence (P25, P36, P50).
