# Supplementary material for: Evidence-based comparative severity assessment in young and adult mice
Source: PLoS One. 2023 Oct 20;18(10):e0285429. doi: 10.1371/journal.pone.0285429 (PMC10588901; doi:10.1371/journal.pone.0285429)
Supplement: S5 Table — a. p-values for correlation analysis (Spearman). C57BL/6J model: adolescence (P25, P36, P50). b. Correlation coefficients (r) for correlation analysis (Spearman). C57BL/6J model: adolescence (P25, P36, P50). (ZIP) [file pone.0285429.s016.zip › S5b_Table.pdf]

	SP_percentage	Bur_120_1	Bur_night_1	Bur_120_2	Bur_night_2	Nesting_Sum	VWR	OF_distance	OF_immobility	OF_rearing	OF_jumps	OF_wall	OF_center	Irwin_Sum	Temperature	Fcm
**SP_percentage**	1.000	-0.040	-0.170	-0.060	-0.170	-0.140	-0.390	-0.080	0.080	-0.260	-0.120	0.010	-0.150	-0.150	-0.170	0.050
**Bur_120_1**	-0.040	1.000	0.280	0.130	0.300	0.010	0.090	0.010	0.230	0.210	0.020	-0.130	-0.130	-0.100	0.110	-0.380
**Bur_night_1**	-0.170	0.280	1.000	0.450	0.810	0.040	0.540	0.450	-0.030	0.300	0.180	-0.120	0.000	0.230	0.030	-0.500
**Bur_120_2**	-0.060	0.130	0.450	1.000	0.410	-0.080	0.150	0.310	-0.140	0.260	-0.080	-0.110	0.040	0.270	0.000	-0.180
**Bur_night_2**	-0.170	0.300	0.810	0.410	1.000	-0.040	0.430	0.370	0.020	0.260	0.240	-0.230	0.010	0.200	0.090	-0.390
**Nesting_Sum**	-0.140	0.010	0.040	-0.080	-0.040	1.000	0.370	0.180	-0.230	0.100	0.040	0.090	-0.010	0.150	0.320	-0.070
**VWR**	-0.390	0.090	0.540	0.150	0.430	0.370	1.000	0.430	-0.160	0.500	0.270	-0.080	0.090	0.100	0.290	-0.370
**OF_distance**	-0.080	0.010	0.450	0.310	0.370	0.180	0.430	1.000	-0.610	0.430	0.360	0.010	0.000	0.050	0.240	-0.170
**OF_immobility**	0.080	0.230	-0.030	-0.140	0.020	-0.230	-0.160	-0.610	1.000	0.050	-0.080	-0.140	-0.160	-0.280	-0.070	-0.210
**OF_rearing**	-0.260	0.210	0.300	0.260	0.260	0.100	0.500	0.430	0.050	1.000	0.300	-0.110	-0.060	-0.270	0.280	-0.230
**OF_jumps**	-0.120	0.020	0.180	-0.080	0.240	0.040	0.270	0.360	-0.080	0.300	1.000	0.210	-0.230	-0.090	0.070	-0.110
**OF_wall**	0.010	-0.130	-0.120	-0.110	-0.230	0.090	-0.080	0.010	-0.140	-0.110	0.210	1.000	-0.590	0.110	0.060	0.140
**OF_center**	-0.150	-0.130	0.000	0.040	0.010	-0.010	0.090	0.000	-0.160	-0.060	-0.230	-0.590	1.000	-0.010	-0.060	0.060
**Irwin._Sum**	-0.150	-0.100	0.230	0.270	0.200	0.150	0.100	0.050	-0.280	-0.270	-0.090	0.110	-0.010	1.000	-0.010	-0.060
**Temperature**	-0.170	0.110	0.030	0.000	0.090	0.320	0.290	0.240	-0.070	0.280	0.070	0.060	-0.060	-0.010	1.000	0.100
**Fcm**	0.050	-0.380	-0.500	-0.180	-0.390	-0.070	-0.370	-0.170	-0.210	-0.230	-0.110	0.140	0.060	-0.060	0.100	1.000

Table S5b. Correlation coefficients (r) for correlation analysis (Spearman). C57BL/6J model: adolescence (P25, P36, P50).
