# Supplementary material for: Evidence-based comparative severity assessment in young and adult mice
Source: PLoS One. 2023 Oct 20;18(10):e0285429. doi: 10.1371/journal.pone.0285429 (PMC10588901; doi:10.1371/journal.pone.0285429)
Supplement: S6 Table. a. p-values for correlation analysis (Spearman). C57BL/6J model: P120. b. Correlation coefficients (r) for correlation analysis (Spearman). C57BL/6J model: P120 — (ZIP) [file pone.0285429.s017.zip › S6a_Table.pdf]

	SP_percentage	Bur_120_1	Bur_night_1	Bur_120_2	Bur_night_2	Nesting_Sum	VWR	OF_distance	OF_immobility	OF_rearing	OF_jumps	OF_wall	OF_center	Irwin_Sum	Temperature	Fcm
**SP_percentage**	NA	0.281	0.536	0.982	0.671	0.366	0.000	0.552	0.058	0.810	0.114	0.560	0.313	0.502	0.100	0.109
**Bur_120_1**	0.281	NA	0.121	0.810	0.316	0.207	0.066	0.229	0.983	0.139	0.923	0.366	0.293	0.207	0.090	0.975
**Bur_night_1**	0.536	0.121	NA	0.002	0.000	0.845	0.454	0.308	0.054	0.011	0.059	0.008	0.046	0.054	0.831	0.754
**Bur_120_2**	0.982	0.810	0.002	NA	0.002	0.821	0.031	0.832	0.731	0.304	0.468	0.722	0.228	0.970	0.497	0.253
**Bur_night_2**	0.671	0.316	0.000	0.002	NA	0.074	0.176	0.721	0.007	0.066	0.072	0.009	0.057	0.230	0.679	0.295
**Nesting_Sum**	0.366	0.207	0.845	0.821	0.074	NA	0.194	0.988	0.397	0.809	0.022	0.560	0.739	0.504	0.000	0.437
**VWR**	0.000	0.066	0.454	0.031	0.176	0.194	NA	0.916	0.101	0.423	0.104	0.630	0.633	0.570	0.015	0.002
**OF_distance**	0.552	0.229	0.308	0.832	0.721	0.988	0.916	NA	0.043	0.003	0.611	0.634	0.678	0.366	0.584	0.764
**OF_immobility**	0.058	0.983	0.054	0.731	0.007	0.397	0.101	0.043	NA	0.620	0.000	0.154	0.542	0.307	0.805	0.284
**OF_rearing**	0.810	0.139	0.011	0.304	0.066	0.809	0.423	0.003	0.620	NA	0.749	0.040	0.425	0.156	0.392	0.355
**OF_jumps**	0.114	0.923	0.059	0.468	0.072	0.022	0.104	0.611	0.000	0.749	NA	0.897	0.207	0.971	0.420	0.077
**OF_wall**	0.560	0.366	0.008	0.722	0.009	0.560	0.630	0.634	0.154	0.040	0.897	NA	0.001	0.148	0.224	0.866
**OF_center**	0.313	0.293	0.046	0.228	0.057	0.739	0.633	0.678	0.542	0.425	0.207	0.001	NA	0.769	0.141	0.617
**Irwin._Sum**	0.502	0.207	0.054	0.970	0.230	0.504	0.570	0.366	0.307	0.156	0.971	0.148	0.769	NA	0.525	0.804
**Temperature**	0.100	0.090	0.831	0.497	0.679	0.000	0.015	0.584	0.805	0.392	0.420	0.224	0.141	0.525	NA	0.004
**Fcm**	0.109	0.975	0.754	0.253	0.295	0.437	0.002	0.764	0.284	0.355	0.077	0.866	0.617	0.804	0.004	NA

Table S6a. p-values for correlation analysis (Spearman). C57BL/6J model: P120.
