# Supplementary material for: Evidence-based comparative severity assessment in young and adult mice
Source: PLoS One. 2023 Oct 20;18(10):e0285429. doi: 10.1371/journal.pone.0285429 (PMC10588901; doi:10.1371/journal.pone.0285429)
Supplement: S6 Table. a. p-values for correlation analysis (Spearman). C57BL/6J model: P120. b. Correlation coefficients (r) for correlation analysis (Spearman). C57BL/6J model: P120 — (ZIP) [file pone.0285429.s017.zip › S6b_Table.pdf]

	SP_percentage	Bur_120_1	Bur_night_1	Bur_120_2	Bur_night_2	Nesting_Sum	VWR	OF_distance	OF_immobility	OF_rearing	OF_jumps	OF_wall	OF_center	Irwin_Sum	Temperature	Fcm
**SP_percentage**	1.000	0.170	-0.100	0.000	-0.070	-0.150	-0.800	-0.100	-0.300	-0.040	0.250	-0.100	0.160	0.110	-0.260	-0.260
**Bur_120_1**	0.170	1.000	0.250	-0.040	0.160	0.200	-0.290	0.190	0.000	0.240	-0.020	-0.150	0.170	0.200	0.270	-0.010
**Bur_night_1**	-0.100	0.250	1.000	0.470	0.780	0.030	-0.120	0.170	0.310	0.400	-0.300	0.410	-0.320	0.310	0.030	0.050
**Bur_120_2**	0.000	-0.040	0.470	1.000	0.470	-0.040	-0.340	0.030	0.060	0.170	-0.120	0.060	-0.200	0.010	-0.110	-0.190
**Bur_night_2**	-0.070	0.160	0.780	0.470	1.000	0.290	-0.220	0.060	0.420	0.290	-0.290	0.410	-0.300	0.190	-0.070	-0.170
**Nesting_Sum**	-0.150	0.200	0.030	-0.040	0.290	1.000	0.210	0.000	0.140	-0.040	-0.360	-0.090	0.050	-0.110	0.570	0.130
**VWR**	-0.800	-0.290	-0.120	-0.340	-0.220	0.210	1.000	0.020	0.260	-0.130	-0.260	0.080	-0.080	-0.090	0.380	0.470
**OF_distance**	-0.100	0.190	0.170	0.030	0.060	0.000	0.020	1.000	-0.320	0.460	0.080	0.080	-0.070	-0.150	0.090	0.050
**OF_immobility**	-0.300	0.000	0.310	0.060	0.420	0.140	0.260	-0.320	1.000	-0.080	-0.570	0.230	0.100	0.170	0.040	0.170
**OF_rearing**	-0.040	0.240	0.400	0.170	0.290	-0.040	-0.130	0.460	-0.080	1.000	-0.050	0.330	-0.130	0.230	-0.140	-0.150
**OF_jumps**	0.250	-0.020	-0.300	-0.120	-0.290	-0.360	-0.260	0.080	-0.570	-0.050	1.000	-0.020	-0.200	-0.010	-0.130	-0.280
**OF_wall**	-0.100	-0.150	0.410	0.060	0.410	-0.090	0.080	0.080	0.230	0.330	-0.020	1.000	-0.520	0.230	-0.200	0.030
**OF_center**	0.160	0.170	-0.320	-0.200	-0.300	0.050	-0.080	-0.070	0.100	-0.130	-0.200	-0.520	1.000	0.050	0.240	0.080
**Irwin._Sum**	0.110	0.200	0.310	0.010	0.190	-0.110	-0.090	-0.150	0.170	0.230	-0.010	0.230	0.050	1.000	0.100	-0.040
**Temperature**	-0.260	0.270	0.030	-0.110	-0.070	0.570	0.380	0.090	0.040	-0.140	-0.130	-0.200	0.240	0.100	1.000	0.450
**Fcm**	-0.260	-0.010	0.050	-0.190	-0.170	0.130	0.470	0.050	0.170	-0.150	-0.280	0.030	0.080	-0.040	0.450	1.000

Table S6b. Correlation coefficients (r) for correlation analysis (Spearman). C57BL/6J model: P120.
