# Supplementary material for: Evidence-based comparative severity assessment in young and adult mice
Source: PLoS One. 2023 Oct 20;18(10):e0285429. doi: 10.1371/journal.pone.0285429 (PMC10588901; doi:10.1371/journal.pone.0285429)
Supplement: S7 Table — a. p-values for correlation analysis (Spearman). Genetic models: early adolescence. b. Correlation coefficients (r) for correlation analysis (Spearman). Genetic models: early adolescence. (ZIP) [file pone.0285429.s018.zip › S7a_Table.pdf]

	Clinical_score	SP_ percentage	Nesting_Sum	Homecage_ feeding	Homecage_ drinking	OF_ distance	OF_ immobility	OF_ rearing	OF_jumps	OF_wall	OF_center	Irwin	Temperature	Fcm
**Clinical_score**	NA	0.379	0.143	0.031	0.046	0.567	0.679	0.727	0.852	0.260	0.889	0.021	0.829	0.162
**SP_percentage**	0.379	NA	0.033	0.119	0.004	0.505	0.636	0.039	0.359	0.000	0.001	0.691	0.371	0.015
**Nesting_Sum**	0.143	0.033	NA	0.036	0.017	0.000	0.000	0.571	0.208	0.018	0.019	0.651	0.000	0.003
**Homecage_feeding**	0.031	0.119	0.036	NA	0.000	0.502	0.709	0.084	0.103	0.707	0.828	0.101	0.034	0.855
**Homecage_drinking**	0.046	0.004	0.017	0.000	NA	0.785	0.741	0.171	0.194	0.018	0.048	0.369	0.773	0.104
**OF_distance**	0.567	0.505	0.000	0.502	0.785	NA	0.000	0.000	0.910	0.014	0.014	0.612	0.043	0.743
**OF_immobility**	0.679	0.636	0.000	0.709	0.741	0.000	NA	0.000	0.877	0.027	0.019	0.944	0.041	0.669
**OF_rearing**	0.727	0.039	0.571	0.084	0.171	0.000	0.000	NA	0.025	0.000	0.020	0.725	0.016	0.001
**OF_jumps**	0.852	0.359	0.208	0.103	0.194	0.910	0.877	0.025	NA	0.760	0.977	0.666	0.697	0.020
**OF_wall**	0.260	0.000	0.018	0.707	0.018	0.014	0.027	0.000	0.760	NA	0.000	0.288	0.185	0.001
**OF_center**	0.889	0.001	0.019	0.828	0.048	0.014	0.019	0.020	0.977	0.000	NA	0.537	0.033	0.006
**Irwin**	0.021	0.691	0.651	0.101	0.369	0.612	0.944	0.725	0.666	0.288	0.537	NA	0.251	0.837
**Temperature**	0.829	0.371	0.000	0.034	0.773	0.043	0.041	0.016	0.697	0.185	0.033	0.251	NA	0.768
**Fcm**	0.162	0.015	0.003	0.855	0.104	0.743	0.669	0.001	0.020	0.001	0.006	0.837	0.768	NA

Table S7a. p-values for correlation analysis (Spearman). Genetic models: early adolescence.
