# Supplementary material for: Evidence-based comparative severity assessment in young and adult mice
Source: PLoS One. 2023 Oct 20;18(10):e0285429. doi: 10.1371/journal.pone.0285429 (PMC10588901; doi:10.1371/journal.pone.0285429)
Supplement: S7 Table — a. p-values for correlation analysis (Spearman). Genetic models: early adolescence. b. Correlation coefficients (r) for correlation analysis (Spearman). Genetic models: early adolescence. (ZIP) [file pone.0285429.s018.zip › S7b_Table.pdf]

	Clinical_score	SP_ percentage	Nesting_Sum	Homecage_ feeding	Homecage_ drinking	OF_distance	OF_immobility	OF_rearing	OF_jumps	OF_wall	OF_center	Irwin	Temperature	Fcm
**Clinical_score**	1.000	-0.100	0.170	-0.250	-0.230	-0.070	0.050	-0.040	0.020	0.130	-0.020	0.260	-0.020	-0.160
**SP_percentage**	-0.100	1.000	-0.240	0.180	0.320	0.080	-0.050	-0.230	-0.110	-0.400	0.370	-0.050	-0.100	0.270
**Nesting_Sum**	0.170	-0.240	1.000	-0.240	-0.270	-0.480	0.460	-0.070	0.140	0.270	-0.260	-0.050	0.450	-0.330
**Homecage_feeding**	-0.250	0.180	-0.240	1.000	0.390	0.080	-0.040	-0.200	-0.190	-0.040	0.020	-0.190	0.240	0.020
**Homecage_drinking**	-0.230	0.320	-0.270	0.390	1.000	-0.030	0.040	-0.160	-0.150	-0.270	0.220	-0.100	-0.030	0.190
**OF_distance**	-0.070	0.080	-0.480	0.080	-0.030	1.000	-0.910	0.570	0.010	0.280	-0.280	0.060	-0.230	0.040
**OF_immobility**	0.050	-0.050	0.460	-0.040	0.040	-0.910	1.000	-0.540	0.020	-0.250	0.270	-0.010	0.230	-0.050
**OF_rearing**	-0.040	-0.230	-0.070	-0.200	-0.160	0.570	-0.540	1.000	0.250	0.440	-0.260	0.040	-0.270	-0.370
**OF_jumps**	0.020	-0.110	0.140	-0.190	-0.150	0.010	0.020	0.250	1.000	0.040	0.000	0.050	-0.040	-0.260
**OF_wall**	0.130	-0.400	0.270	-0.040	-0.270	0.280	-0.250	0.440	0.040	1.000	-0.770	0.120	0.150	-0.380
**OF_center**	-0.020	0.370	-0.260	0.020	0.220	-0.280	0.270	-0.260	0.000	-0.770	1.000	-0.070	-0.240	0.310
**Irwin**	0.260	-0.050	-0.050	-0.190	-0.100	0.060	-0.010	0.040	0.050	0.120	-0.070	1.000	-0.130	0.020
**Temperature**	-0.020	-0.100	0.450	0.240	-0.030	-0.230	0.230	-0.270	-0.040	0.150	-0.240	-0.130	1.000	0.030
**Fcm**	-0.160	0.270	-0.330	0.020	0.190	0.040	-0.050	-0.370	-0.260	-0.380	0.310	0.020	0.030	1.000

Table S7b. Correlation coefficients (r) for correlation analysis (Spearman). Genetic models: early adolescence.
