# Supplementary material for: Evidence-based comparative severity assessment in young and adult mice
Source: PLoS One. 2023 Oct 20;18(10):e0285429. doi: 10.1371/journal.pone.0285429 (PMC10588901; doi:10.1371/journal.pone.0285429)
Supplement: S8 Table — a. p-values for correlation analysis (Spearman). Genetic models: late adolescence. b. Correlation coefficients (r) for correlation analysis (Spearman). Genetic models: late adolescence. (ZIP) [file pone.0285429.s019.zip › S8a_Table.pdf]

	Clinical_score	SP_ percentage	Bur_night	Nesting_Sum	Homecage_ feeding	Heimk_ drinking	OF_distance	OF_ immobility	OF_rearing	OF_jumps	OF_wall	OF_center	Irwin	Temperature	Fcm
**Clinical_score**	NA	NA	NA	NA	NA	NA	NA	NA	NA	NA	NA	NA	NA	NA	NA
**SP_percentage**	NA	NA	0.000	0.000	0.000	0.015	0.905	0.860	0.104	0.000	0.000	0.000	0.302	0.022	0.286
**Bur_night**	NA	0.000	NA	0.157	0.000	0.000	0.000	0.000	0.000	0.000	0.059	0.332	0.063	0.000	0.245
**Nesting_Sum**	NA	0.000	0.157	NA	0.205	0.605	0.000	0.000	0.032	0.367	0.000	0.000	0.402	0.348	0.064
**Homecage_feeding**	NA	0.000	0.000	0.205	NA	0.000	0.284	0.450	0.003	0.000	0.064	0.284	0.035	0.142	0.056
**Heimk_ drinking**	NA	0.015	0.000	0.605	0.000	NA	0.000	0.003	0.000	0.000	0.590	0.856	0.007	0.069	0.711
**OF_distance**	NA	0.905	0.000	0.000	0.284	0.000	NA	0.000	0.000	0.015	0.740	0.695	0.000	0.039	0.289
**OF_immobility**	NA	0.860	0.000	0.000	0.450	0.003	0.000	NA	0.000	0.042	0.674	0.380	0.011	0.001	0.775
**OF_rearing**	NA	0.104	0.000	0.032	0.003	0.000	0.000	0.000	NA	0.001	0.145	0.355	0.001	0.044	0.434
**OF_jumps**	NA	0.000	0.000	0.367	0.000	0.000	0.015	0.042	0.001	NA	0.001	0.005	0.056	0.008	0.210
**OF_wall**	NA	0.000	0.059	0.000	0.064	0.590	0.740	0.674	0.145	0.001	NA	0.000	0.082	0.000	0.390
**OF_center**	NA	0.000	0.332	0.000	0.284	0.856	0.695	0.380	0.355	0.005	0.000	NA	0.142	0.000	0.142
**Irwin**	NA	0.302	0.063	0.402	0.035	0.007	0.000	0.011	0.001	0.056	0.082	0.142	NA	0.132	0.844
**Temperature**	NA	0.022	0.000	0.348	0.142	0.069	0.039	0.001	0.044	0.008	0.000	0.000	0.132	NA	0.041
**Fcm**	NA	0.286	0.245	0.064	0.056	0.711	0.289	0.775	0.434	0.210	0.390	0.142	0.844	0.041	NA

Table S8a. p-values for correlation analysis (Spearman). Genetic models: late adolescence.
