# Supplementary material for: Evidence-based comparative severity assessment in young and adult mice
Source: PLoS One. 2023 Oct 20;18(10):e0285429. doi: 10.1371/journal.pone.0285429 (PMC10588901; doi:10.1371/journal.pone.0285429)
Supplement: S8 Table — a. p-values for correlation analysis (Spearman). Genetic models: late adolescence. b. Correlation coefficients (r) for correlation analysis (Spearman). Genetic models: late adolescence. (ZIP) [file pone.0285429.s019.zip › S8b_Table.pdf]

	Clinical_score	SP_percentage	Bur_night	Nesting_Sum	Homecage_feeding	Heimk_drinking	OF_distance	OF_immobility	OF_rearing	OF_jumps	OF_wall	OF_center	Irwin	Temperature	Fcm
**Clinical_score**	1.000	NA	NA	NA	NA	NA	NA	NA	NA	NA	NA	NA	NA	NA	NA
**SP_percentage**	NA	1.000	0.720	-0.520	0.390	0.280	-0.010	-0.020	-0.190	-0.460	-0.570	0.450	-0.120	0.260	-0.120
**Bur_night**	NA	0.720	1.000	0.230	0.680	0.800	-0.710	0.700	-0.650	-0.630	-0.300	0.160	-0.300	0.540	0.190
**Nesting_Sum**	NA	-0.520	0.230	1.000	-0.140	0.060	-0.510	0.520	-0.240	0.100	0.490	-0.410	0.100	-0.110	0.210
**Homecage_feeding**	NA	0.390	0.680	-0.140	1.000	0.520	-0.120	0.090	-0.340	-0.490	-0.210	0.120	-0.240	0.170	0.220
**Heimk_ drinking**	NA	0.280	0.800	0.060	0.520	1.000	-0.440	0.330	-0.550	-0.480	-0.060	0.020	-0.300	0.210	0.040
**OF_distance**	NA	-0.010	-0.710	-0.510	-0.120	-0.440	1.000	-0.930	0.790	0.270	0.040	-0.050	0.400	-0.230	-0.120
**OF_immobility**	NA	-0.020	0.700	0.520	0.090	0.330	-0.930	1.000	-0.720	-0.230	-0.050	0.100	-0.290	0.360	0.030
**OF_rearing**	NA	-0.190	-0.650	-0.240	-0.340	-0.550	0.790	-0.720	1.000	0.380	0.170	-0.110	0.360	-0.230	-0.090
**OF_jumps**	NA	-0.460	-0.630	0.100	-0.490	-0.480	0.270	-0.230	0.380	1.000	0.370	-0.310	0.220	-0.300	-0.140
**OF_wall**	NA	-0.570	-0.300	0.490	-0.210	-0.060	0.040	-0.050	0.170	0.370	1.000	-0.890	0.200	-0.400	0.100
**OF_center**	NA	0.450	0.160	-0.410	0.120	0.020	-0.050	0.100	-0.110	-0.310	-0.890	1.000	-0.170	0.470	-0.170
**Irwin**	NA	-0.120	-0.300	0.100	-0.240	-0.300	0.400	-0.290	0.360	0.220	0.200	-0.170	1.000	-0.170	0.020
**Temperature**	NA	0.260	0.540	-0.110	0.170	0.210	-0.230	0.360	-0.230	-0.300	-0.400	0.470	-0.170	1.000	-0.230
**Fcm**	NA	-0.120	0.190	0.210	0.220	0.040	-0.120	0.030	-0.090	-0.140	0.100	-0.170	0.020	-0.230	1.000

Table S8b. Correlation coefficients (r) for correlation analysis (Spearman). Genetic models: late adolescence.
