# Supplementary material for: Evidence-based comparative severity assessment in young and adult mice
Source: PLoS One. 2023 Oct 20;18(10):e0285429. doi: 10.1371/journal.pone.0285429 (PMC10588901; doi:10.1371/journal.pone.0285429)
Supplement: S9 Table — Genetic models. (PDF) [file pone.0285429.s020.pdf]

Early adolescence			Late adolescence		
**Position**	**Parameter**	**Frequency**	**Position**	**Parameter**	**Frequency**
1	Fcm	21	1	Bur_night	8
	Heimk_drinking	6		Fcm	31
	Homecage_feeding	15		Heimk_drinking	2
	Irwin	2		Irwin	42
	OF_distance	1		OF_jumps	3
	OF_jumps	13		Temp	14
	SP_percentage	2	2	Bur_night	9
	Temp	40		Fcm	44
2	Fcm	16		Heimk_drinking	12
	Heimk_drinking	8		Homecage_feeding	4
	Homecage_feeding	39		Irwin	20
	Irwin	1		OF_jumps	3
	Nesting_sum	2		Temp	8
	OF_distance	1	3	Bur_night	23
	OF_jumps	5		Fcm	14
	Temp	28		Heimk_drinking	25
3	Fcm	21		Homecage_feeding	6
	Heimk_drinking	8		Irwin	6
	Homecage_feeding	38		Nesting_sum	1
	Irwin	3		OF_jumps	4
	Nesting_sum	4		OF_wall	13
	OF_distance	5		Temp	8
	OF_jumps	4	4	Bur_night	11
	OF_wall	4		Fcm	8
	Temp	13		Heimk_drinking	23
4	Fcm	7		Homecage_feeding	16
	Heimk_drinking	10		Irwin	3
	Homecage_feeding	5		Nesting_sum	9
	Irwin	2		OF_jumps	4
	Nesting_sum	27		OF_wall	18

Table S9. Top 30 parameters after 100 PCA runs of the training set. Genetic models.
