# Supplementary material for: Evidence-based comparative severity assessment in young and adult mice
Source: PLoS One. 2023 Oct 20;18(10):e0285429. doi: 10.1371/journal.pone.0285429 (PMC10588901; doi:10.1371/journal.pone.0285429)
Supplement: S10 Table — (PDF) [file pone.0285429.s021.pdf]

Group	Cluster	*Gria1* model: early adolescence n	%	*Scn1a* model early adolescence n	%	*Gria1* model late adolescence n	%	*Scn1a* model late adolescence n	%
**Wild-type**	4	31	1.95	119	7.51	42	2.64	26	1.64
	3	498	31.26	575	36.28	763	47.90	223	14.07
	2	666	41.81	521	32.87	782	49.09	520	32.81
	1	398	24.98	370	23.34	6	0.38	816	51.48
**Genetic deficiency**	4	85	5.92	566	35.69	60	4.18	662	41.74
	3	354	24.65	616	38.84	740	51.53	579	36.51
	2	542	37.74	321	20.24	574	39.97	320	20.18
	1	455	31.69	83	5.23	62	4.32	25	1.58

Table S10. Results from the *k*-means cluster allocation in the genetic models.
